# Fritsch–Buttenberg–Wiechell rearrangement of magnesium alkylidene carbenoids leading to the formation of alkynes

**DOI:** 10.3762/bjoc.17.94

**Published:** 2021-05-28

**Authors:** Tsutomu Kimura, Koto Sekiguchi, Akane Ando, Aki Imafuji

**Affiliations:** 1 Department of Chemistry, Graduate School of Science, Tokyo University of Science, 1-3 Kagurazaka, Shinjuku-ku, Tokyo 162-8601, Japan

**Keywords:** alkyne, 1-chlorovinyl *p*-tolyl sulfoxide, DFT calculation, Fritsch–Buttenberg–Wiechell rearrangement, magnesium alkylidene carbenoid

## Abstract

A series of 1-heteroatom-substituted vinyl *p*-tolyl sulfoxides were prepared and treated with organometallic reagents to evaluate which combination of sulfoxides and organometallic reagents yielded alkynes the most efficiently. The use of 1-chlorovinyl *p*-tolyl sulfoxide and isopropylmagnesium chloride was optimal for this purpose. A variety of 1-chlorovinyl *p*-tolyl sulfoxides were prepared from carbonyl compounds and chloromethyl *p*-tolyl sulfoxide and were converted into alkynes via the sulfoxide/magnesium exchange reaction and subsequent Fritsch–Buttenberg–Wiechell (FBW) rearrangement of the resulting magnesium alkylidene carbenoids. The mechanism of the FBW rearrangement of magnesium alkylidene carbenoids was studied by using ^13^C-labeled sulfoxides and by using DFT calculations.

## Introduction

**Scheme 1:**
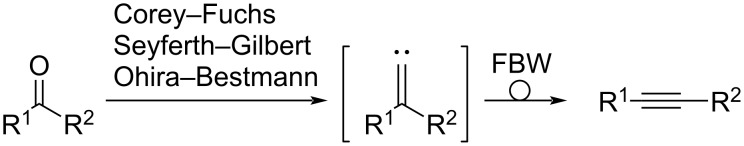
Synthesis of alkynes from carbonyl compounds through one-carbon homologation.

Alkynes are important compounds in organic synthesis [1]. Electrophilic additions of alkynes give functionalized compounds, and cycloadditions such as the Huisgen reaction afford cyclic compounds. Weakly acidic terminal alkynes can be deprotonated, and the resulting acetylides are used as carbon nucleophiles. Terminal alkynes are also used for the Sonogashira coupling reaction with organic halides. The dehydrohalogenation of *gem*-dihaloalkanes, *vic*-dihaloalkanes, and haloalkenes with strong bases is often used for the synthesis of alkynes [2]. An alternative strategy for the synthesis of alkynes is the conversion of carbonyl compounds through one-carbon homologation [3]. Much effort has been devoted to developing a procedure for the synthesis of alkynes from carbonyl compounds, and the Corey–Fuchs method, the Seyferth–Gilbert method, and the Ohira–Bestmann modification have been developed (Scheme 1) [4–7]. These methods consist of the Wittig or Horner–Wadsworth–Emmons (HWE) reaction of carbonyl compounds with organophosphorus reagents containing carbene sources and the 1,2-rearrangement of alkylidene carbenes and related species, referred to as Fritsch–Buttenberg–Wiechell (FBW) rearrangement [8–9]. While alkynes are synthesized from carbonyl compounds with these methods, the use of butyllithium for the generation of lithium alkylidene carbenoids limits the range of usable substrates in the Corey–Fuchs method, and the Ohira–Bestmann method cannot be applied to the synthesis of internal alkynes from ketones [3].

**Scheme 2:**
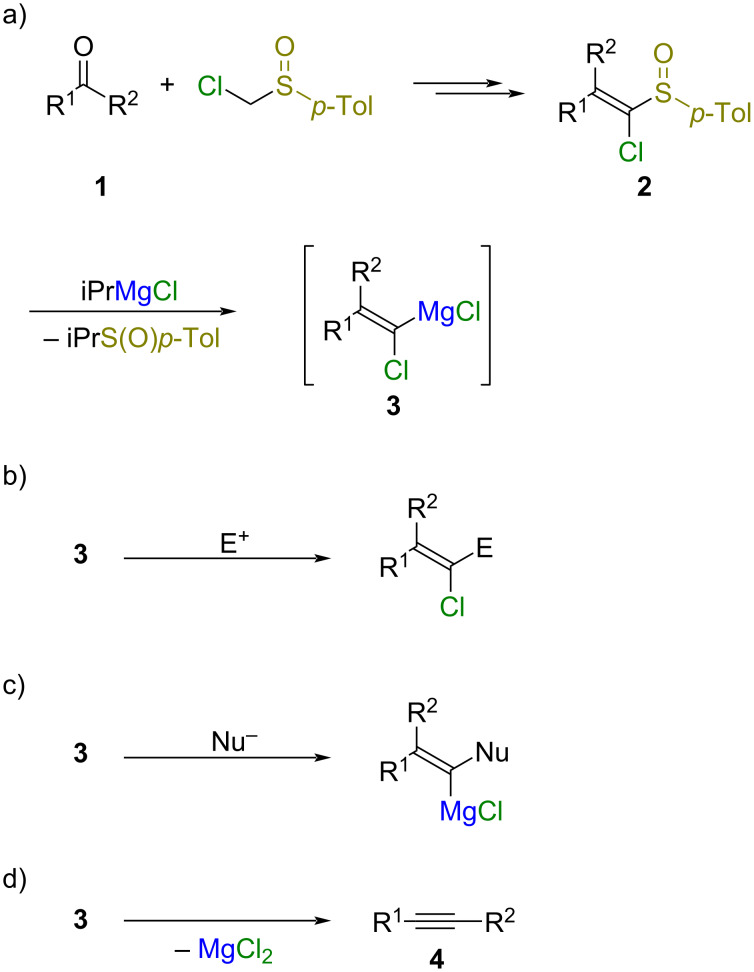
Reactions of magnesium alkylidene carbenoids **3**, generated from sulfoxides **2** and iPrMgCl.

Magnesium alkylidene carbenoids **3** are reactive intermediates in which both magnesium and chlorine atoms are attached to an alkene carbon atom (Scheme 2a) [10]. Magnesium alkylidene carbenoids **3** can be generated from isopropylmagnesium chloride and 1-chlorovinyl *p*-tolyl sulfoxides **2**, which are prepared from carbonyl compounds **1** and chloromethyl *p*-tolyl sulfoxide, via the sulfoxide/magnesium exchange reaction [11]. During the course of our study on the ambiphilic reactions of magnesium alkylidene carbenoids **3** (Scheme 2b and 2c) [12–15], we found that the FBW rearrangement of magnesium alkylidene carbenoids **3** occurred to give alkynes **4** when magnesium alkylidene carbenoids **3** were left without reactants (Scheme 2d). Herein, we report the synthesis of alkynes **4** from carbonyl compounds **1** with one-carbon homologation via the FBW rearrangement of magnesium alkylidene carbenoids **3** as a key step. A mechanistic study on the FBW rearrangement of magnesium alkylidene carbenoids **3** by using ^13^C-labeled sulfoxides and by using DFT calculations is also described.

## Results and Discussion

### Synthesis of 1-heteroatom-substituted vinyl *p*-tolyl sulfoxides

**Scheme 3:**
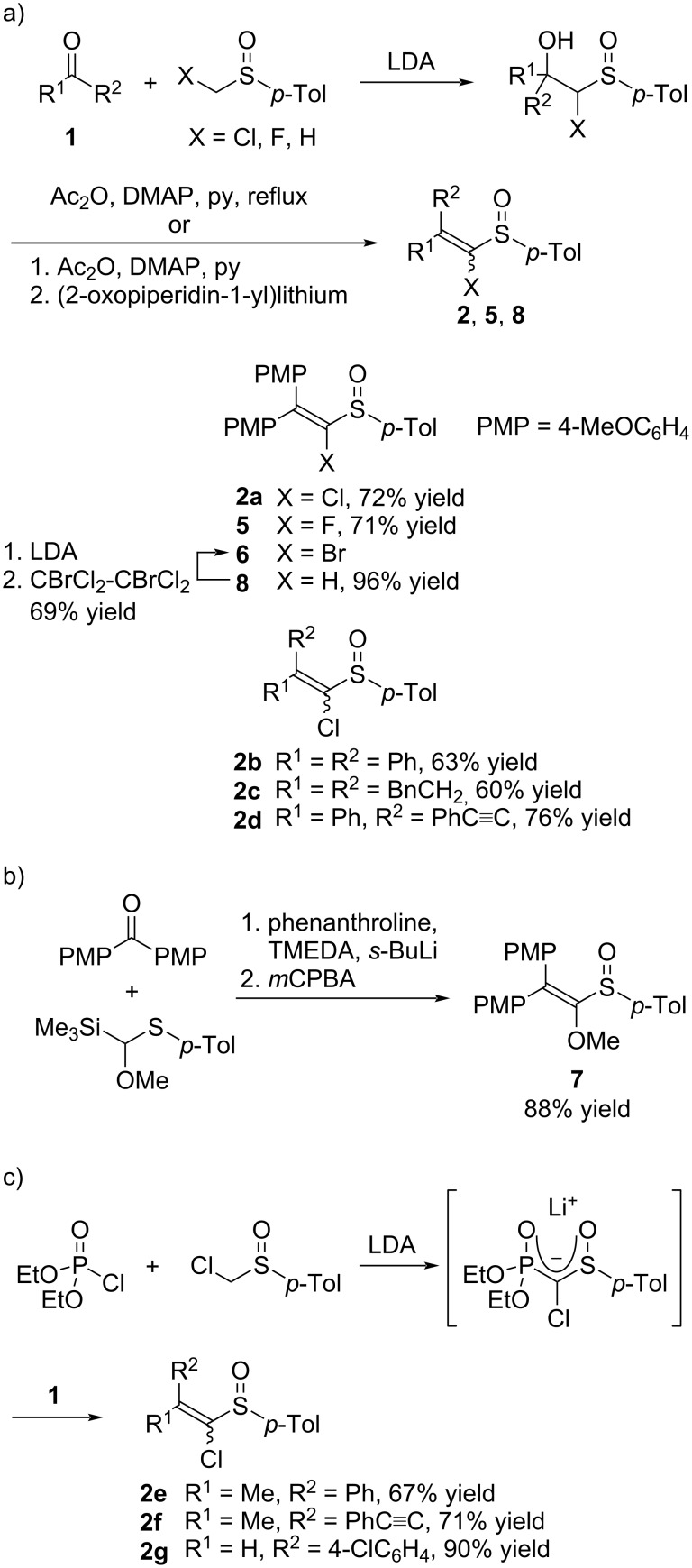
Synthesis of sulfoxides **2** and **5**–**8** from carbonyl compounds **1**.

As magnesium alkylidene carbenoid precursors, a series of 1-heteroatom-substituted vinyl *p*-tolyl sulfoxides **2**, **5**–**7** and 1-unsubstituted sulfoxide **8** were prepared from carbonyl compounds **1** (Scheme 3). The 2,2-bis(4-methoxyphenyl)-substituted sulfoxides **2a**, **5**, and **8** were prepared via the nucleophilic addition of [(*p*-tolylsulfinyl)methyl]lithiums to 4,4'-dimethoxybenzophenone, acylation of the hydroxy group in the adducts, and β-elimination (Scheme 3a) [16]. The 1-bromovinyl *p*-tolyl sulfoxide **6** was prepared by the deprotonation of sulfoxide **8** with LDA followed by electrophilic bromination with 1,2-dibromo-1,1,2,2-tetrachloroethane. 1-Methoxyvinyl *p*-tolyl sulfoxide **7** was prepared by a Peterson olefination of 4,4'-dimethoxybenzophenone with methoxy(trimethylsilyl)methyl *p*-tolyl sulfide, and oxidation of the resulting 1-methoxyvinyl *p*-tolyl sulfide with *m*CPBA (Scheme 3b) [17]. The 1-chlorovinyl *p*-tolyl sulfoxides **2e**–**g** were prepared through a one-pot procedure (Scheme 3c) [15]. The HWE reagent was generated in situ from chloromethyl *p*-tolyl sulfoxide, diethyl chlorophosphate, and LDA, and the reaction of the HWE reagent with carbonyl compounds gave sulfoxides **2e**–**g**. The 2,2-disubstituted sulfoxides **2b**–**d**, which could not be prepared by the one-pot HWE reaction, were prepared through a stepwise method (Scheme 3a). The 2,2-unsymmetrically substituted sulfoxides **2d**–**g** were obtained as mixtures of geometric isomers that were separated by column chromatography on silica gel.

### FBW rearrangement of magnesium alkylidene carbenoids

**Scheme 4:**
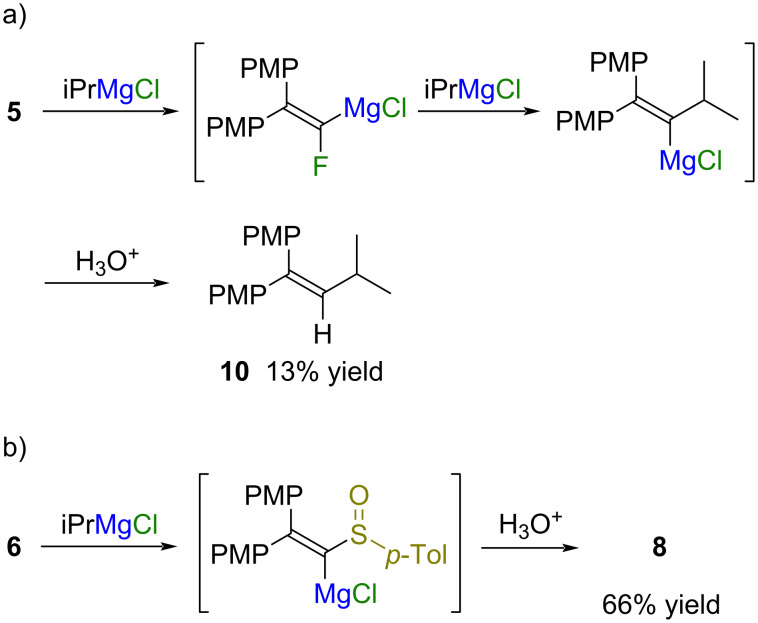
Reaction of sulfoxides **5** and **6** with isopropylmagnesium chloride.

Based on the results of DFT calculations, the degree of the vinylidene character of 1-heteroatom-substituted vinylmetals is dependent on the type of the metal and the heteroatom substituent [18–19]. The vinylidene character of 1-heteroatom-substituted vinylmetals is estimated to increase in the following order (1-methoxyvinyl)magnesium chloride, CH_2_=C(OMe)MgCl < (1-halovinyl)magnesium chlorides, CH_2_=CXMgCl (X = F, Cl, and Br) << (1-chlorovinyl)lithium, CH_2_=CClLi. If the 1-heteroatom-substituted vinylmetal displays vinylidene characteristics, the FBW rearrangement occurs to give the alkyne. If not, the vinylmetal is simply protonated to give a heteroatom-substituted alkene. A series of 1-heteroatom-substituted vinyl *p*-tolyl sulfoxides **2a**, **5**–**7**, and 1-unsubstituted sulfoxide **8** were treated with organometallic reagents with the aim of finding the optimal combination of the metal and heteroatom substituent for the FBW rearrangement (Table 1). The 2,2-bis(4-methoxyphenyl)-substituted 1-chlorovinyl *p*-tolyl sulfoxide **2a** was added to isopropylmagnesium chloride in toluene at −78 °C, and the mixture was warmed to 0 °C for 2 h (Table 1, entry 1). Excess isopropylmagnesium chloride (3.5 equivalents) was necessary for the complete consumption of the sulfoxide. As a result, isopropyl *p*-tolyl sulfoxide and alkyne **4a** were obtained in 97% yield and 99% yield, respectively. This result shows that both the sulfoxide/magnesium exchange reaction and the FBW rearrangement occurred with high efficiency. A similar reaction of sulfoxide **2a** with *sec*-butyllithium also gave alkyne **4a** in 97% yield (Table 1, entry 2). On the other hand, the sulfoxide/zinc exchange reaction of sulfoxide **2a** with isopropylzinc bromide did not proceed at all even under reflux conditions (Table 1, entry 3). In the case of the reaction of 1-fluorovinyl *p*-tolyl sulfoxide **5** with isopropylmagnesium chloride, the S_N_V reaction of (1-fluorovinyl)magnesium chloride with isopropylmagnesium chloride competed with the FBW rearrangement to give a mixture of alkyne **4a** and alkene **10** in 69% and 13% yield, respectively (Table 1, entry 4, Scheme 4a, and Scheme 2c) [20]. Both the sulfoxide/magnesium exchange reaction and bromine/magnesium exchange reaction occurred when 1-bromovinyl *p*-tolyl sulfoxide **6** was treated with isopropylmagnesium chloride, and debrominated sulfoxide **8** was obtained as a major product (Table 1, entry 5 and Scheme 4b). The reaction of the 1-methoxy-substituted sulfoxide **7** with isopropylmagnesium chloride gave the alkene **9d** as the major product along with a small amount of alkyne **4a** (Table 1, entry 6). Most of (1-methoxyvinyl)magnesium chloride, which is expected to have vinylidene characteristics to a lesser extent, remained without rearrangement [19]. The reaction of *p*-tolyl vinyl sulfoxide **8** having no leaving group with isopropylmagnesium chloride gave the desulfinylated product **9e** in 79% yield, and the starting sulfoxide **8** was recovered in 10% yield (Table 1, entry 7). Considering the reaction efficiency, accessibility of precursors, and functional group tolerance, the use of isopropylmagnesium chloride and 1-chlorovinyl *p*-tolyl sulfoxide **2a** was the optimal combination of organometallic reagent and carbenoid precursor.

**Table 1:** Reaction of sulfoxides **2a** and **5**–**8** with organometallic reagents.

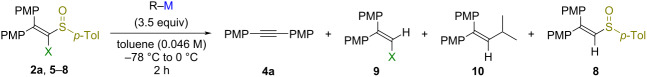								

entry	**2**	X	R–M	yield of **4a** (%)	**9**	yield of **9** (%)	yield of **10** (%)	yield of **8** (%)

1	**2a**	Cl	iPrMgCl	99	**9a**	0	0	0
2	**2a**	Cl	*s*-BuLi	97	**9a**	0	0	0
3^a^	**2a**	Cl	iPrZnBr	0	**9a**	0	0	0
4	**5**	F	iPrMgCl	69	**9b**	0	13	0
5	**6**	Br	iPrMgCl	32	**9c**	0	0	66
6	**7**	MeO	iPrMgCl	4	**9d**	95	0	0
7	**8**	H	iPrMgCl	0	**9e**	79	0	10

^a^The reaction was carried out under reflux conditions.

**Scheme 5:**
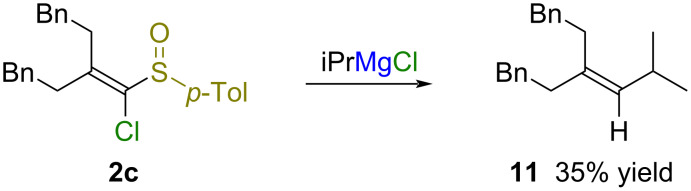
Reaction of sulfoxide **2c** with isopropylmagnesium chloride.

The substrate scope was explored with a variety of sulfoxides **2b**–**g** (Table 2). The 2,2-diphenyl-substituted sulfoxide **2b** was converted into alkyne **4b** by treatment with isopropylmagnesium chloride in 99% yield (Table 2, entry 1). In contrast, the treatment of 2,2-dialkyl-substituted sulfoxide **2c** with isopropylmagnesium chloride gave a complex mixture including alkene **11** with an isopropyl group, which arose from the S_N_V reaction, in 35% yield (Table 2, entry 2, and Scheme 5). To compare the difference in reactivity between geometric isomers, the reaction of 2,2-unsymmetrically substituted sulfoxides **2d**–**g** with isopropylmagnesium chloride was carried out (Table 2, entries 3–10). There was no significant difference in the reactivity between the geometric isomers of 2-alkynyl-2-phenyl-substituted sulfoxide **2d** (Table 2, entries 3 and 4). The reaction of a mixture comprising the geometric isomers *L*-**2d** and *M*-**2d** with isopropylmagnesium chloride gave alkyne **4d** in 83% yield. On the other hand, there was a difference in the reactivity between geometric isomers of 2-methyl-substituted sulfoxides **2e** and **2f** (Table 2, entries 5–8). The reaction of (*Z*)-sulfoxides (*Z*)-**2e** and (*Z*)-**2f**, in which the methyl group and chloro group were located *trans* to each other, tended to give alkynes **4** with low efficiency (Table 2, entries 6 and 8). The terminal alkyne **4g** was obtained from both geometric isomers of 2-monoaryl-substituted sulfoxide **2g** in good yields (Table 2, entries 9 and 10).

**Table 2:** Synthesis of alkynes **4b**–**g** from sulfoxides **2b**–**g**.

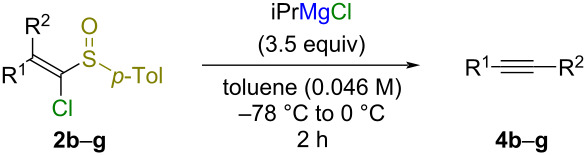					

entry	**2**	R^1^	R^2^	**4**	yield (%)

1	**2b**	Ph	Ph	**4b**	99
2^a^	**2c**	BnCH_2_	BnCH_2_	**4c**	0
3^b^	*L*-**2d**	Ph, PhC≡C		**4d**	86
4^c^	*M*-**2d**	Ph, PhC≡C		**4d**	88
5	(*E*)-**2e**	Me	Ph	**4e**	91
6	(*Z*)-**2e**	Ph	Me	**4e**	62
7	(*E*)-**2f**	Me	PhC≡C	**4f**	95
8	(*Z*)-**2f**	PhC≡C	Me	**4f**	63
9	(*E*)-**2g**	4-ClC_6_H_4_	H	**4g**	87
10	(*Z*)-**2g**	H	4-ClC_6_H_4_	**4g**	98

^a^5-Methyl-1-phenyl-3-(2-phenylethyl)hex-3-ene (**11**) was obtained in 35% yield; ^b^*L*-**2d**: less polar geometric isomer, *R*_f_ = 0.47 (hexane/EtOAc 3:1); ^c^*M*-**2d**: more polar geometric isomer, *R*_f_ = 0.44 (hexane/EtOAc 3:1).

### Reaction mechanism

**Scheme 6:**
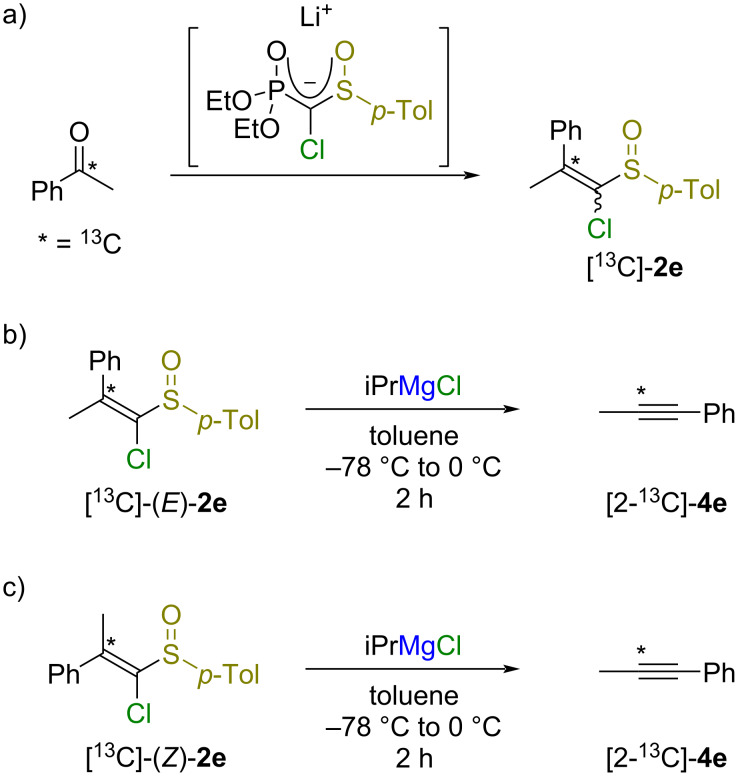
Reaction of ^13^C-labeled sulfoxides [^13^C]-(*E*)-**2e** and [^13^C]-(*Z*)-**2e** with iPrMgCl.

The substrate scope results suggest that aryl and alkynyl groups are migratable, whereas alkyl groups are not. The FBW rearrangement of magnesium alkylidene carbenoids seems to be an anionotropic rearrangement because the trend of migratory aptitude (aryl, alkynyl >> alkyl) follows the trend of the stability of carbanions [21–22]. The *trans*-relationship between the chloro group and the migrating substituent appears to be of importance for a successful 1,2-rearrangement [8]. Given the difference in reactivity between geometric isomers, the formation of alkynes via the 1,2-rearrangement of free alkylidene carbenes is unlikely. To gain insight into the reaction mechanism, ^13^C-labeled sulfoxides [^13^C]-(*E*)-**2e** and [^13^C]-(*Z*)-**2e** were prepared from acetophenone-α-^13^C and HWE reagent (Scheme 6a), and each geometric isomer was subjected to the reaction with isopropylmagnesium chloride [23–26]. Alkyne **4e**, having a ^13^C-labeled carbon atom at the 2-position of the propy-1-ynyl group, was exclusively formed in both cases (Scheme 6b and c) [27]. These results demonstrated that the C–Ph bond was cleaved and that the C–Me bond was maintained during the reaction.

**Scheme 7:**
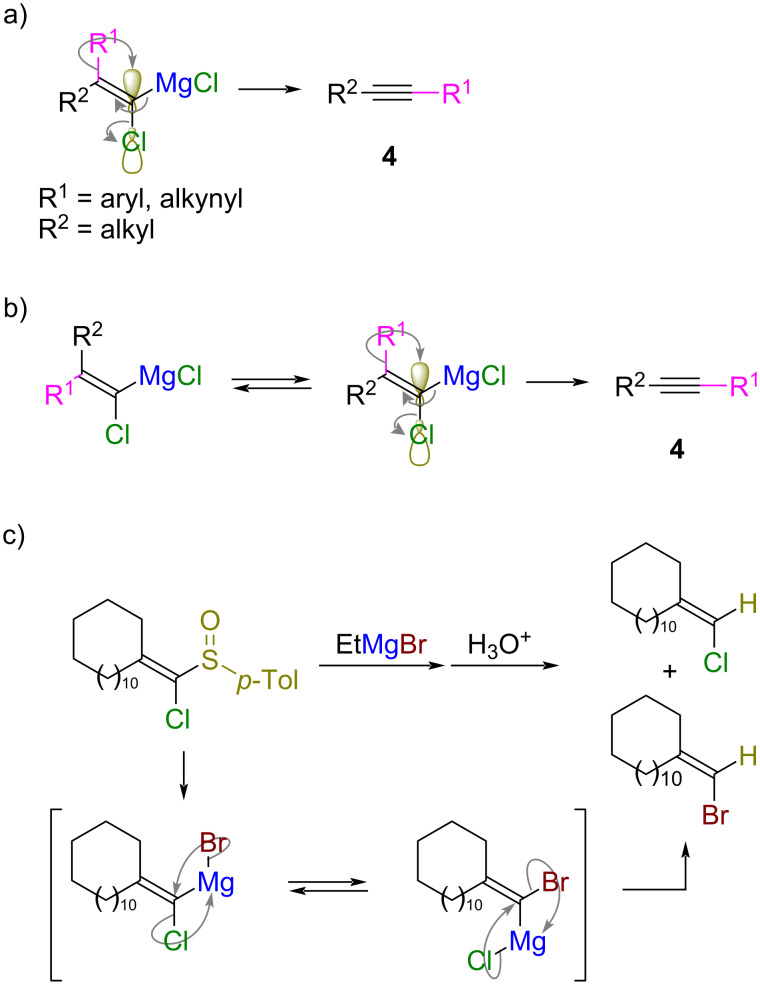
A plausible reaction mechanism for the formation of alkynes **4**. a) 1,2-Rearrangement readily takes place with substrates with *trans*-oriented Cl-substituent to the migratable group R^1^. b) Formation of product **4** from geometric cis isomer is possible due to the equilibrium between magnesium alkylidene carbenoids. c) Halogen exchange between a 1-chlorovinyl *p*-tolyl sulfoxide and ethylmagnesium bromide [16].

Based on these results, we propose the reaction mechanism as outlined in Scheme 7. When substituent R^1^ with a high migratory aptitude, such as an aryl group and alkynyl group, is located *trans* to the chloro group, the 1,2-rearrangement smoothly occurs to give alkynes **4** because substituent R^1^ can readily approach the C–Cl antibonding orbital (Scheme 7a). On the other hand, when substituent R^1^ is located *cis* to the chloro group and a nonmigratable alkyl group R^2^ occupies the *trans* position, the 1,2-rearrangement does not occur directly. Geometric isomers of magnesium alkylidene carbenoids are in equilibrium [16], and the 1,2-rearrangement occurs giving the alkynes **4** when the substituent R^1^ is located *trans* to the chloro group (Scheme 7b). An exchange of halogen atoms was observed in the reaction of a 1-chlorovinyl *p*-tolyl sulfoxide with ethylmagnesium bromide (Scheme 7c) [16]. The nucleophilic substitution of the halide on the magnesium atom with the carbenoid carbon atom having a halogen substituent is a plausible mechanism for the isomerization.

**Figure 1:**
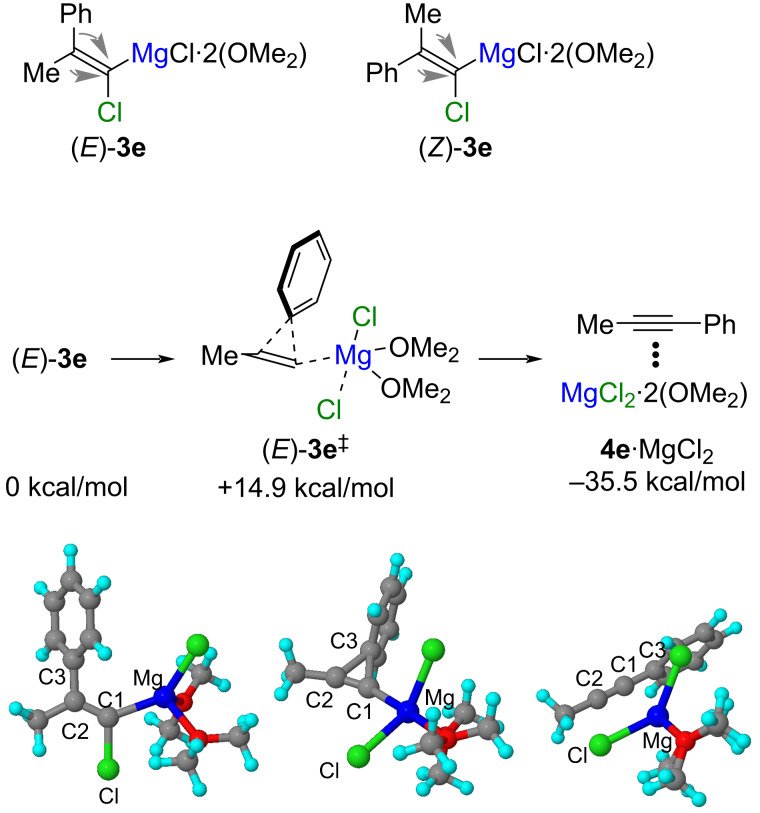
Optimized geometries of reactant (*E*)-**3e**, transition state (*E*)-**3e^‡^**, and product **4e**·MgCl_2_ for the FBW rearrangement of magnesium alkylidene carbenoid.

The FBW rearrangement of magnesium alkylidene carbenoids was studied by using DFT calculations (Figure 1) [28–32]. The calculations were performed using the 6-311G++(d,p) basis set at the B3LYP level of theory [33–36]. Bis(dimethyl ether)-solvated (1-chloro-2-phenylprop-1-enyl)magnesium chlorides (*E*)-**3e** and (*Z*)-**3e** were used as model compounds. The 1,2-shift of both the phenyl group and methyl group to the carbenoid carbon atom was examined for each geometric isomer to locate the transition state [37]. While appropriate transition state structures were not found for the 1,2-shift of the methyl group in (*E*)-**3e** and the 1,2-shift of substituents in magnesium alkylidene carbenoid (*Z*)-**3e**, transition state structure (*E*)-**3e^‡^** was found for the 1,2-shift of phenyl group in magnesium alkylidene carbenoid (*E*)-**3e**. The geometry around the carbenoid carbon atom in magnesium alkylidene carbenoids (*E*)-**3e** (C–Cl: 1.86 Å, C=C–Cl: 115°, C=C–Mg: 135°) significantly deviated from that of the standard sp^2^-hybridized carbon atom, suggesting that magnesium alkylidene carbenoids (*E*)-**3e** have partial vinylidene characteristics [19]. In the transition state structure (*E*)-**3e^‡^**, the ipso carbon atom (C3) of the phenyl group was located at the middle between the C=C bond (C2–C3: 1.72 Å, C1–C3: 1.70 Å), and the C1–Cl bond (C1···Cl: 3.22 Å) was cleaved. The activation energy for this reaction was estimated to be 14.9 kcal/mol. The IRC calculation revealed that the chlorine atom gradually dissociated from the carbenoid carbon atom as the phenyl group approached the carbenoid carbon atom [38–39]. Then, three carbon atoms (C1, C2, and C3) were arranged linearly to give alkyne **4e**.

## Conclusion

We studied the FBW rearrangement of magnesium alkylidene carbenoids. The use of isopropylmagnesium chloride and 1-chlorovinyl *p*-tolyl sulfoxides was found to be the optimal combination for the generation of magnesium alkylidene carbenoids and the subsequent FBW rearrangement. Because 1-chlorovinyl *p*-tolyl sulfoxides can be readily prepared from carbonyl compounds and chloromethyl *p*-tolyl sulfoxides, the FBW rearrangement of magnesium alkylidene carbenoids generated from 1-chlorovinyl *p*-tolyl sulfoxides provides a convenient route to the synthesis of alkynes from carbonyl compounds through one-carbon homologation. The *trans* relationship between the substituent with a high migratory aptitude and the chloro group was of importance for the successful 1,2-rearrangement.

## Experimental

All reactions involving air- or water-sensitive compounds were conducted under an argon atmosphere. Argon gas was dried by passage through P_2_O_5_. Anhydrous THF and toluene were purchased and used as supplied. Silica gel 60N containing 0.5% fluorescence reagent 254 and a quartz column were used for column chromatography, and the products that absorbed UV light were detected by UV irradiation. The melting points were measured using a Yanaco MP-S3 apparatus and are uncorrected. IR spectra were recorded on a Perkin–Elmer Frontier FTIR in the ATR mode. NMR spectra were recorded in CDCl_3_ solutions using a JEOL JNM-LA 300, JEOL JNM-LA 500, Bruker AVANCE DPX 300, and Bruker AVANCE DPX 400 spectrometers. Mass spectrometry (MS) experiments were conducted at 70 eV by direct injection with a HITACHI M-80B mass spectrometer. Fast atom bombardment (FAB) mass spectra were obtained with a mixture of *m*-nitrobenzyl alcohol and glycerol as the matrix. Chloromethyl *p*-tolyl sulfoxide was prepared according to the procedure described in the literature [40]. Alkyne **4a** is a known compound, and the characterization data were in agreement with those reported in the literature [41].

### Typical procedure for the synthesis of sulfoxides **2**

A solution of chloromethyl *p*-tolyl sulfoxide (4.16 g, 22.0 mmol) in THF (20 mL) was added dropwise to a solution of LDA (2.0 mol/L solution in THF/heptane/ethylbenzene, 12.0 mL, 24 mmol) in THF (30 mL) at −78 °C, and the mixture was stirred at −78 °C for 10 min. Then, a solution of 4,4'-dimethoxybenzophenone (4.85 g, 20.0 mmol) in THF (40 mL) was added dropwise to the mixture at −78 °C, and the reaction mixture was warmed to −20 °C over a period of 2 h. The reaction was quenched with sat. aq. NH_4_Cl (20 mL), and the mixture was extracted with CHCl_3_ (3 × 20 mL). The combined organic layers were washed with water (50 mL), dried over Na_2_SO_4_, and concentrated under reduced pressure. The residue was purified by column chromatography on silica gel (hexane/EtOAc 1:1) to give the alcohol [6.90 g, 16.7 mmol, 83%, *R*_f_ = 0.45 (hexane/EtOAc 1:1)] as a single diastereomer; yellow solid; mp 102.5–104.0 °C; IR (ATR) 3558, 3004, 2965, 2932, 2905, 2837, 1606, 1585, 1512, 1461, 1307, 1250, 1178, 1156, 1085, 1055, 1028, 838, 801, 754 cm^−1^; ^1^H NMR (301 MHz, CDCl_3_) δ 2.42 (s, 3H), 3.76 (s, 3H), 3.82 (s, 3H), 4.19 (s, 1H), 5.23 (s, 1H), 6.83 (d, *J* = 8.9 Hz, 2H), 6.94 (d, *J* = 8.9 Hz, 2H), 7.29–7.42 (m, 6H), 7.55 (d, *J* = 8.9 Hz, 2H); ^13^C NMR (126 MHz, CDCl_3_) δ 21.8, 55.2, 55.3, 80.8, 85.3, 113.6, 114.1, 124.6, 126.9, 127.4, 129.9, 135.2, 136.5, 138.0, 142.4, 158.9, 159.2; FABMS (*m/z*, %): 453 ([M + Na]^+^, 3), 431 ([M + H]^+^, 3), 413 (6), 274 (100), 227 (28); HRMS–FAB^+^ (*m*/*z*): [M + H]^+^ calcd for C_23_H_24_^35^ClO_4_S, 431.1084; found, 431.1085. A mixture of the alcohol (1.66 g, 3.84 mmol), acetic anhydride (7.5 mL), pyridine (16.0 mL), and DMAP (93 mg, 0.76 mmol) was stirred under reflux for 24 h. The reaction mixture was concentrated under reduced pressure, and the residue was purified by column chromatography on silica gel (hexane/EtOAc) to give sulfoxide **2a** [1.38 g, 3.34 mmol, 87%, *R*_f_ = 0.50 (hexane/EtOAc 1:1)]. Colorless solid; mp 140.2–141.0 °C; IR (ATR) 3063, 3032, 3024, 2955, 2932, 2908, 2837, 1603, 1577, 1506, 1462, 1306, 1246, 1172, 1082, 1049, 1031, 829 cm^−1^; ^1^H NMR (399 MHz, CDCl_3_) δ 2.42 (s, 3H), 3.79 (s, 3H), 3.85 (s, 3H), 6.84 (d, *J* = 8.8 Hz, 2H), 6.95 (d, *J* = 8.8 Hz, 2H), 7.23 (d, *J* = 8.8 Hz, 2H), 7.27–7.33 (m, 4H), 7.51 (d, *J* = 8.2 Hz, 2H); ^13^C NMR (100 MHz, CDCl_3_) δ 21.5, 55.3, 55.4, 113.4, 114.0, 125.0, 129.7, 130.9, 131.0, 131.5, 131.7, 135.0, 139.3, 141.5, 149.4, 160.1, 160.4; FABMS (*m/z*, %): 413 ([M + H]^+^, 100), 364 (20), 238 (22); HRMS–FAB^+^ (*m*/*z*): [M + H]^+^ calcd for C_23_H_22_^35^ClO_3_S, 413.0978; found, 413.0980.

### Typical procedure for the reaction of sulfoxides **2** with iPrMgCl

A solution of sulfoxide **2a** (207 mg, 0.501 mmol) in toluene (10 mL) was added dropwise to a solution of iPrMgCl (2.0 mol/L solution in Et_2_O, 0.875 mL, 1.75 mmol) in toluene (15 mL) at −78 °C, and the reaction mixture was warmed to 0 °C over 2 h. The reaction was quenched with sat. aq. NH_4_Cl (8 mL) and extracted with CHCl_3_ (3 × 5 mL). The combined organic layers were washed with water (30 mL), dried over Na_2_SO_4_, filtered, and concentrated under reduced pressure. The residue was purified by column chromatography on silica gel (hexane/EtOAc 5:1) to give **4a** [118 mg, 0.495 mmol, 99%, *R*_f_ = 0.4 (hexane/EtOAc 3:1)]. Colorless solid; mp 139.2–140.1 °C; IR (ATR) 2969, 2938, 2841, 1607, 1511, 1457, 1444, 1287, 1244, 1172, 1106, 1025, 834, 820 cm^−1^; ^1^H NMR (301 MHz, CDCl_3_) δ 3.83 (s, 6H), 6.87 (d, *J* = 8.8 Hz, 4H), 7.45 (d, *J* = 8.8 Hz, 4H); ^13^C NMR (126 MHz, CDCl_3_) δ 55.3, 88.0, 114.0, 115.7, 132.9, 159.4; EIMS (*m/z*, %): 238 ([M]^+^, 100), 223 (55); HRMS–EI (*m*/*z*): [M]^+^ calcd for C_16_H_14_O_2_, 238.0994; found, 238.0997.

## Supporting Information

File 1 Characterization data, copies of NMR spectra, and computational details.
