# Supplementary material for: Fritsch–Buttenberg–Wiechell rearrangement of magnesium alkylidene carbenoids leading to the formation of alkynes
Source: Beilstein J Org Chem. 2021 May 28;17:1352–9. doi: 10.3762/bjoc.17.94 (PMC8182682; doi:10.3762/bjoc.17.94)
Supplement: File 1 — Characterization data, copies of NMR spectra, and computational details. [file Beilstein_J_Org_Chem-17-1352-s001.pdf]

BEILSTEIN JOURNAL OF ORGANIC CHEMISTRY

Supporting Information

for

Fritsch–Buttenberg–Wiechell rearrangement of magnesium alkylidene carbenoids leading to the formation of alkynes

Tsutomu Kimura, Koto Sekiguchi, Akane Ando and Aki Imafuji

Beilstein J. Org. Chem. **2021,** *17,* 1352–1359. doi:10.3762/bjoc.17.94

Characterization data, copies of NMR spectra, and computational details

License and Terms: This is a supporting information file under the terms of the Creative Commons Attribution License (https://creativecommons.org/licenses/by/4.0). Please note that the reuse, redistribution and reproduction in particular requires that the author(s) and source are credited and that individual graphics may be subject to special legal provisions.
The license is subject to the *Beilstein Journal of Organic Chemistry* terms and conditions: (https://www.beilstein-journals.org/bjoc/terms)

Table of contents

Page

Characterization data of sulfoxides, alkynes, and alkenes S2

^{1}H and ^{13}C NMR spectra of sulfoxides, alkynes, and alkenes S8

Cartesian coordinates of model compounds (*E*)-**3e**, (*Z*)-**3e**, (*E*)-**3e**‡, **4e**·$MgCl_2$ S37

Selected geometric parameters of model compounds.. S40

IRC calculations S40

Experimental

Sulfoxides **2b** [1], (*E*)-**2e** and (*Z*)-**2e** [1], (*E*)-**2f** and (*Z*)-**2f** [2], and (*E*)-**2g** and (*Z*)-**2g** [3], alkynes **4b** [4], **4d** [5], **4e** [6], **4f** [7], and **4g** [8], and alkenes **9d** [9] and **9e** [10] are known compounds. Characterization data were in agreement with those reported in the literature.

4,4'-[2-Fluoro-2-(*p*-tolylsulfinyl)ethene-1,1-diyl]bis(methoxybenzene) (5). R_f = 0.45 (hexane/EtOAc, 1:1); colorless solid; mp 110.3–111.1 ºC; IR (ATR) 2964, 2927, 2841, 1605, 1507, 1250, 1175, 1106, 1032 cm^{-1}; ^{1}H NMR (500 MHz, $CDCl_3$) δ 2.43 (s, 3H), 3.79 (s, 3H), 3.88 (s, 3H), 6.82 (d, *J* = 8.6 Hz, 2H), 6.99 (d, *J* = 8.6 Hz, 2H), 7.24 (d, *J* = 8.6 Hz, 2H), 7.30 (d, *J* = 8.6 Hz, 2H), 7.34 (d, *J* = 8.0 Hz, 2H), 7.61 (d, *J* = 8.0 Hz, 2H); ^{13}C NMR (126 MHz, $CDCl_3$) δ 21.5, 55.3, 55.4, 113.7, 114.1, 125.3, 127.1 (d, *J* = 4.8 Hz), 127.4 (d, *J* = 2.4 Hz), 130.0, 131.0 (d, *J* = 6.0 Hz), 131.7 (d, *J* = 6.0 Hz), 132.3 (d, *J* = 2.4 Hz), 137.4, 141.9, 154.4 (d, *J* = 323.9 Hz), 160.2, 160.3; MS (FAB$^+$) *m/z* (%) 397 ([M+H]$^+$, 100), 380 (13), 348 (17), 273 (20); HRMS (FAB$^+$) calcd for $C_{23}H_{22}FO_3S$ ([M+H]$^+$): 397.1274, found: 397.1275.

4,4'-[2-Bromo-2-(*p*-tolylsulfinyl)ethene-1,1-diyl]bis(methoxybenzene) (6). A solution of sulfoxide **8** (189 mg, 0.499 mmol) in THF (1 mL) was added dropwise to a solution of LDA (1.3 mmol) in THF (4.5 mL) over a period of 4 min at −78 °C, and the mixture was stirred at −78 °C for 10 min. A solution of 1,2-dibromo-1,1,2,2-tetrachloroethane (814 mg, 2.50 mmol) in THF (2.5 mL) was added to the mixture at −78 °C, and the reaction mixture was warmed to 0 °C over a period of 30 min. The reaction mixture was stirred at 0 °C for 1 h, and the reaction was quenched with aq sat NH_4Cl (1 mL). The mixture was extracted with $CHCl_3$ (10 mL) and water (10 mL), and the aqueous layer was extracted with $CHCl_3$ (2 × 3 mL). The combined organic layer was dried over Na_2SO_4, and concentrated under reduced pressure. The residue was purified by column chromatography on silica gel (hexane/EtOAc 2:1 to 1:1) to give sulfoxide **6** [158 mg, 0.345 mmol, 69%, R_f = 0.60 (hexane/EtOAc 1:1)]. Yellow solid; mp 143.5–144.4 ºC; IR (ATR) 3024, 2955, 2904, 2838, 1604, 1503, 1460, 1302, 1250, 1172, 1083, 1058, 1025, 808 cm^{-1}; 1H NMR (301 MHz, $CDCl_3$) δ 2.42 (s, 3H), 3.81 (s, 3H), 3.85 (s, 3H), 6.84 (d, *J* = 8.6 Hz, 2H), 6.94 (d, *J* = 8.6 Hz, 2H), 7.20–7.33 (m, 6H), 7.48 (d, *J* = 8.3 Hz, 2H); ^{13}C NMR (100 MHz, $CDCl_3$) δ 21.5, 55.3, 55.4, 113.5, 114.0, 125.0, 129.7, 130.1, 131.3, 131.5, 131.8, 132.8, 140.2, 141.4, 152.9, 160.1, 160.4; MS (FAB$^+$) *m/z* (%) 459 (100), 457 ([M+H]$^+$, 98), 238 (68); HRMS (FAB$^+$) calcd for $C_{23}H_{22}{}^{79}BrO_3S$ ([M+H]$^+$): 457.0473, found: 457.0476.

4,4'-[2-Methoxy-2-(*p*-tolylsulfinyl)ethene-1,1-diyl]bis(methoxybenzene) (7). A 1.0 mol/L solution of *sec*-BuLi in cyclohexane and hexane (1.3 mL, 1.3 mmol) was added to a solution of 1,10-phenanthroline (5.1 mg, 0.028 mmol), TMEDA (125 mg, 1.08 mmol), and methoxy(*p*-tolylthio)(trimethylsilyl)methane (244 mg, 1.01 mmol) in THF (2.5 mL) at −78 °C, and the mixture was stirred at −78 °C for 2.5 h. Then, 4,4'-dimethoxybenzophenone (268 mg, 1.11 mmol) was added to the mixture at −78 °C, and the reaction mixture was stirred at 26 °C for 22 h. The reaction was quenched with sat. aq. NH_4Cl (5 mL) and extracted with $CHCl_3$ (15 mL) and water (5 mL). The aqueous layer was extracted with $CHCl_3$ (3 × 15 mL), and the combined organic layer was washed with brine (5 mL), dried over $MgSO_4$, filtered, and concentrated under reduced pressure. The residue was purified by column chromatography on silica gel (hexane/EtOAc 20:1) to give 1-methoxyvinyl *p*-tolyl sulfide (374 mg, 0.952 mmol, 94%). *m*CPBA (containing water, >65% purity, 192 mg, approximately 1.1 mmol) was added to a solution of 1-methoxyvinyl *p*-tolyl sulfide (374 mg, 0.952 mmol) in $CHCl_3$ (4.8 mL) portionwise at 0 °C over a period of 2 h. The reaction was quenched with sat. aq. Na_2SO_3 (15 mL) and extracted with toluene (15 mL). The organic layer was washed with aq NaOH (5%, 2 × 15 mL), and the combined aqueous layer was extracted with toluene (3 × 15 mL). The combined organic layer was dried over $MgSO_4$, filtered, and concentrated under reduced pressure. The residue was purified by column chromatography on silica gel (hexane/EtOAc 1:1) to give **7** [366 mg, 0.896 mmol, 94%, R_f = 0.30 (hexane/EtOAc 1:1)] as a yellow oil. IR (ATR) 2934, 2835, 1604, 1508, 1461, 1246, 1208, 1173, 1105, 1029, 834, 808 cm^{-1}; ^1H NMR (399 MHz, $CDCl_3$) δ 2.40 (s, 3H), 3.63 (s, 3H), 3.79 (s, 3H), 3.86 (s, 3H), 6.80 (d, *J* = 8.9 Hz, 2H), 6.96 (d, *J* = 8.9 Hz, 2H), 7.22–7.34 (m, 6H), 7.51 (d, *J* = 8.2 Hz, 2H); ^{13}C NMR (126 MHz, $CDCl_3$) δ 21.4, 55.2, 55.3, 63.4, 113.6, 113.9, 124.8, 129.6, 129.8, 130.1, 131.5, 132.2, 135.3, 139.2, 140.8, 155.9, 159.6, 159.8; MS (FAB^+) *m/z* (%) , 431 ($[M+Na]^+$, 25), 409 ($[M+H]^+$, 100), 285 (70), 254 (42), 154 (71), 136 (49); HRMS (FAB^+) calcd for $C_{24}H_{25}O_4S$ ($[M+H]^+$): 409.1474, found: 409.1472.

4,4'-[2-(*p*-Tolylsulfinyl)ethene-1,1-diyl]bis(methoxybenzene) (8). R_f = 0.30 (hexane/EtOAc 1:1); colorless oil; IR (ATR) 2963, 2936, 2838, 1604, 1584, 1507, 1460, 1444, 1300, 1287, 1248, 1173, 1027, 835, 799, 750 cm^{-1}; ^1H NMR (500 MHz, $CDCl_3$) δ 2.42 (s, 3H), 3.81 (s, 3H), 3.88 (s, 3H), 6.64 (s, 1H), 6.83 (d, *J* = 9.0 Hz, 2H), 6.98 (d, *J* = 9.0 Hz, 2H), 7.21 (d, *J* = 9.0 Hz, 2H), 7.30–7.33 (m, 4H), 7.57 (d, *J* = 8.0 Hz, 2H); ^{13}C NMR (126 MHz, $CDCl_3$) δ 21.4, 55.4, 113.7, 113.8, 124.6, 129.6, 130.0, 130.1, 131.2, 131.8, 131.9, 141.0, 142.4, 152.1, 160.3, 160.9; MS (FAB^+) *m/z* (%) 379 ($[M+H]^+$, 100), 362 (17), 330 (23); HRMS (FAB^+) calcd for $C_{23}H_{23}O_3S$ ($[M+H]^+$): 379.1368, found: 379.1369.

[2-Chloro-2-(*p*-tolylsulfinyl)ethene-1,1-diyl]dibenzene (2b). Colorless solid; mp 149.9–150.7 °C; IR (ATR) 3054, 1595, 1489, 1443, 1081, 1053, 1033, 843, 808, 767, 758, 701, 623 cm^{-1}; ^{1}H NMR (500 MHz, $CDCl_3$) δ 2.42 (s, 3H), 7.26–7.28 (m, 2H), 7.32–7.35 (m, 5H), 7.37–7.39 (m, 2H), 7.42–7.47 (m, 3H), 7.51 (d, J = 8.2 Hz, 2H); ^{13}C NMR (126 MHz, $CDCl_3$) δ 21.5, 124.9, 128.3, 128.7, 129.1, 129.2, 129.4, 129.8, 130.0, 137.5, 138.3, 138.4, 138.8, 141.8, 149.5; MS (FAB^+) *m/z* (%) 353 ($[M+H]^+$, 100); HRMS (FAB^+) calcd for $C_{21}H_{18}{}^{35}ClOS$ ($[M+H]^+$): 353.0767, found: 353.0767.

{3-[Chloro(*p*-tolylsulfinyl)methylene]pentane-1,5-diyl}dibenzene (2c). R_f = 0.38 (hexane/EtOAc 1:1); colorless solid; mp 65.9–66.8 °C; IR (ATR) 3025, 2929, 1599, 1494, 1453, 1086, 1054, 804, 746, 694 cm^{-1}; ^{1}H NMR (500 MHz, $CDCl_3$) δ 2.41 (s, 3H), 2.63–2.99 (m, 8H), 7.16–7.35 (m, 14H); ^{13}C NMR (126 MHz, $CDCl_3$) δ 21.5, 32.9, 34.9, 35.3, 35.9, 124.7, 126.4, 126.6, 128.3, 128.4, 128.6, 128.8, 129.7, 135.5, 138.2, 140.0, 140.4, 141.5, 150.2; MS (FAB^+) *m/z* (%) 409 ($[M+H]^+$, 100), 91 (20); HRMS (FAB^+) calcd for $C_{25}H_{26}{}^{35}ClOS$ ($[M+H]^+$): 409.1393, found: 409.1390.

[4-Chloro-4-(*p*-tolylsulfinyl)but-3-en-1-yne-1,3-diyl]dibenzene (2d). *L*-**2d** [R_f = 0.47 (hexane/EtOAc 3:1)]: colorless solid; mp 78.3–79.2 °C; IR (ATR) 3054, 3022, 2921, 2195, 1488, 1442, 1086, 1061, 1027, 1016, 925, 807, 754, 732, 688 cm^{-1}; ^{1}H NMR (500 MHz, $CDCl_3$) δ 2.41 (s, 3H), 7.33 (d, J = 8.3 Hz, 2H), 7.37–7.43 (m, 6H), 7.55–7.57 (m, 2H) , 7.62–7.65 (m, 2H), 7.74 (d, J = 8.3 Hz, 2H); ^{13}C NMR (126 MHz, $CDCl_3$) δ 21.5, 85.7, 101.0, 121.8, 124.3, 127.9, 128.3, 128.6, 128.9, 129.5, 129.6, 130.0, 131.7, 134.9, 139.1, 142.1, 145.6; MS (FAB^+) *m/z* (%) 377 ($[M+H]^+$, 100), 254 (24), 154 (26), 137 (19), 93 (31); HRMS (FAB^+) calcd for $C_{23}H_{18}{}^{35}ClOS$ ($[M+H]^+$): 377.0767, found: 377.0767. *M*-**2d** [R_f = 0.44 (hexane/EtOAc, 3:1)]: colorless solid; mp 113.8-114.4 °C; IR (ATR) 3055, 3003, 2195, 1488, 1442, 1085, 1057, 905, 807, 752, 688, 641 cm^{-1}; ^{1}H NMR (399 MHz, $CDCl_3$) δ 2.40 (s, 3H), 7.29–7.38 (m, 5H), 7.44–7.51 (m, 7H), 7.57–7.60 (m, 2H); ^{13}C NMR (100 MHz, $CDCl_3$) δ 21.5, 86.8, 102.3, 121.8, 124.9, 128.4, 128.8, 129.4, 129.6, 129.7, 129.9, 132.0, 132.6, 134.9, 138.4, 142.1, 144.7; MS (FAB^+) *m/z* (%) 377 ($[M+H]^+$, 100), 202 (17), 154 (18), 136 (15), 93 (19); HRMS (FAB^+) calcd for $C_{23}H_{18}{}^{35}ClOS$ ($[M+H]^+$): 377.0767, found: 377.0766.

(*E*)-1-[(1-Chloro-2-phenylprop-1-en-1-yl)sulfinyl]-4-methylbenzene [(*E*)-2e]. (*E*)-**2e**: Colorless solid; mp 79.7–80.4 °C; IR (ATR) 3054, 3000, 2920, 1594, 1489, 1432, 1083, 1050, 1031, 1015, 900, 813, 761, 702 cm^{-1}; ^{1}H NMR (500 MHz, $CDCl_3$) δ 2.32 (s, 3H), 2.40 (s, 3H), 7.29 (d, J = 8.2 Hz, 2H), 7.34–7.36 (m, 2H), 7.39–7.48 (m, 5H); ^{13}C NMR (126 MHz, $CDCl_3$) δ 21.5, 24.2, 125.0, 128.1,

128.80, 128.81, 129.7, 136.7, 138.7, 138.9, 141.7, 147.8; MS (FAB$^+$) *m/z* (%) 291 ([M+H]$^+$, 100); HRMS (FAB$^+$) calcd for $C_{16}H_{16}{}^{35}ClOS$ ([M+H]$^+$): 291.0610, found: 291.0609. [^{13}C]-(*E*)-**2e**: ^{1}H NMR (500 MHz, $CDCl_3$) δ 2.32 (d, *J* = 6.3 Hz, 3H), 2.40 (s, 3H), 7.29 (d, *J* = 8.6 Hz, 2H), 7.34–7.37 (m, 2H), 7.40–7.47 (m, 5H); ^{13}C NMR (126 MHz, $CDCl_3$) δ 21.4, 24.1 (d, *J* = 42.0 Hz), 124.9, 128.1, 128.7, 128.8, 129.7, 136.6 (d, *J* = 81.6 Hz), 138.6 (d, *J* = 2.4 Hz), 138.8 (d, *J* = 52.8 Hz), 141.6, 147.7 (^{13}C-labeled, *J* = 42.0, 52.8, 81.6 Hz).

(*Z*)-1-[(1-Chloro-2-phenylprop-1-en-1-yl)sulfinyl]-4-methylbenzene [(*Z*)-2e]. (*Z*)-**2e**: Colorless solid; mp 88.3–89.2 °C; IR (ATR) 3060, 2956, 1490, 1435, 1087, 1054, 893 807, 704 cm^{-1}; ^{1}H NMR (301 MHz, $CDCl_3$) δ 2.43 (s, 3H), 2.61 (s, 3H), 7.23–7.41 (m, 7H), 7.57 (d, *J* = 8.3 Hz, 2H); ^{13}C NMR (76 MHz, $CDCl_3$) δ 21.5, 22.7, 124.5, 127.3, 128.4, 128.6, 129.9, 134.9, 138.6, 139.6, 141.8, 145.2; MS (FAB$^+$) *m/z* (%) 291 ([M+H]$^+$, 100); HRMS (FAB$^+$) calcd for $C_{16}H_{16}{}^{35}ClOS$ ([M+H]$^+$): 291.0610, found: 291.0609. [^{13}C]-(*Z*)-**2e**: ^{1}H NMR (500 MHz, $CDCl_3$) δ 2.42 (s, 3H), 2.61 (d, *J* = 6.9 Hz, 3H), 7.24–7.26 (m, 2H), 7.31–7.38 (m, 5H), 7.57 (d, *J* = 8.6 Hz, 2H); ^{13}C NMR (126 MHz, $CDCl_3$) δ 21.5, 22.6 (d, *J* = 42.0 Hz), 124.5, 127.2, 128.4 (d, *J* = 3.6 Hz), 128.5, 129.9, 134.8 (d, *J* = 82.2 Hz), 138.5, 139.5 (d, *J* = 52.8 Hz), 141.8, 145.2 (^{13}C-labeled, *J* = 42.0, 52.8, 82.2 Hz).

(*E*)-1-[(1-Chloro-2-methyl-4-phenylbut-1-en-3-yn-1-yl)sulfinyl]-4-methylbenzene [(*E*)-2f]. Yellow solid; mp 94.0–95.0 °C; IR (ATR) 3061, 2921, 2186, 1594, 1574, 1492, 1446, 1083, 1063, 925, 815, 759, 687 cm^{-1}; ^{1}H NMR (500 MHz, $CDCl_3$) δ 2.18 (s, 3H), 2.41 (s, 3H), 7.31 (d, *J* = 8.0 Hz, 2H), 7.37–7.43 (m, 3H), 7.53–7.55 (m, 2H), 7.63 (d, *J* = 8.0 Hz, 2H); ^{13}C NMR (126 MHz, $CDCl_3$) δ 21.4, 21.5, 85.7, 99.8, 121.9, 124.3, 125.9, 128.6, 129.5, 129.9, 131.7, 139.0, 141.9, 145.1; MS (FAB$^+$): *m/z* (%) 315 [(M+H)$^+$, 100], 192 (26); HRMS (FAB$^+$) calcd for $C_{18}H_{16}{}^{35}ClOS$ ([M+H]$^+$): 315.0610, found: 315.0608.

(*Z*)-1-[(1-Chloro-2-methyl-4-phenylbut-1-en-3-yn-1-yl)sulfinyl]-4-methylbenzene [(*Z*)-2f]. Colorless solid; mp 109.0–110.0 °C; IR (ATR) 3019, 2923, 2232, 2187, 1568, 1489, 1442, 1084, 1053, 1027, 1014, 896, 813, 756, 687 cm^{-1}; ^{1}H NMR (399 MHz, $CDCl_3$) δ 2.41 (s, 3H), 2.49 (s, 3H), 7.31–7.39 (m, 5H), 7.46–7.49 (m, 2H), 7.53 (d, *J* = 8.2 Hz, 2H); ^{13}C NMR (100 MHz, $CDCl_3$) δ 20.5, 21.5, 87.0, 100.0, 121.8, 124.5, 127.3, 128.5, 129.5, 130.0, 132.0, 138.2, 142.0, 142.1; MS (FAB$^+$): *m/z* (%) 315 [(M+H)$^+$, 100]; HRMS (FAB$^+$) calcd for $C_{18}H_{16}{}^{35}ClOS$ ([M+H]$^+$): 315.0610, found: 315.0609.

1,1-Bis(4-methoxyphenyl)-3-methyl-1-butene (10). R_f = 0.25 (hexane/EtOAc 30:1); yellow oil; IR (ATR) 2958, 2932, 2835, 1606, 1509, 1463, 1283, 1240, 1172, 1034, 827, 756 cm^{-1}; ^{1}H NMR (399 MHz, $CDCl_3$) δ 1.00 (d, J = 6.6 Hz, 6H), 2.45 (d of sepet, J = 10.0, 6.6 Hz, 1H), 3.78 (s, 3H), 3.83 (s, 3H), 5.75 (d, J = 10.0 Hz, 1H), 6.79 (d, J = 8.8 Hz, 2H), 6.90 (d, J = 8.8 Hz, 2H), 7.09 (d, J = 8.8 Hz, 2H), 7.14 (d, J = 8.8 Hz, 2H); ^{13}C NMR (126 MHz, $CDCl_3$) δ 23.4, 28.7, 55.2, 55.3, 113.4, 113.5, 128.3, 130.8, 133.1, 135.6, 135.9, 138.1, 158.4, 158.6; MS (EI$^+$) *m*/*z* (%) 282 (M$^+$, 100), 267 (30), 251 (21); HRMS (EI$^+$) calcd for $C_{19}H_{22}O_2$: 282.1620, found: 282.1621.

1,2-Diphenylethyne (4b). Colorless solid; mp 57.2–57.8 °C; IR (ATR) 3064, 1600, 1493, 1443, 1070, 1026, 918, 754, 688 cm^{-1}; ^{1}H NMR (400 MHz, $CDCl_3$) δ 7.32–7.37 (m, 6H), 7.52–7.54 (m, 4H); ^{13}C NMR (126 MHz, $CDCl_3$) δ 89.4, 123.3, 128.3, 128.4, 131.6; MS (EI): *m*/*z* (%) 178 (M$^+$, 100); HRMS (EI) calcd for $C_{14}H_{10}$: 178.0783, found: 178.0783.

5-Methyl-1-phenyl-3-(2-phenylethyl)hex-3-ene (11). R_f = 0.57 (hexane/EtOAc 30:1); colorless oil; IR (ATR) 3063, 3029, 2954, 2932, 2864, 1603, 1496, 1453, 744, 696 cm^{-1}; ^{1}H NMR (500 MHz, $CDCl_3$) δ 0.85 (d, J = 6.6 Hz, 6H), 2.27–2.30 (m, 2H), 2.33–2.37 (m, 4H), 2.46 (d of sepet, J = 9.5, 6.6 Hz, 1H), 2.66–2.73 (m, 4H), 4.96 (d, J = 9.5 Hz, 1H), 7.17–7.19 (m, 6H), 7.25–7.30 (m, 4H); ^{13}C NMR (100 MHz, $CDCl_3$) δ 23.4, 27.0, 32.6, 35.0, 35.3, 38.6, 125.7, 125.8, 128.2, 128.3, 128.4, 128.5, 134.1, 135.2, 142.4, 142.5; MS (EI$^+$) *m*/*z* (%) 91 (M$^+$, 100), 278 (58); HRMS (EI$^+$) calcd for $C_{21}H_{26}$: 278.2035, found: 278.2034.

1,4-Diphenylbuta-1,3-diyne (4d). iPrMgCl (2.5 equiv) and *t*-BuMgCl (2.5 equiv) were used. Yellow solid; mp 83.1–83.7 °C; IR (ATR) 3049, 2925, 2852, 2150, 1593, 1570, 1485, 1439, 1178, 1157, 1068, 1025, 916, 752, 683 cm^{-1}; ^{1}H NMR (400 MHz, $CDCl_3$) δ 7.31–7.39 (m, 6H), 7.52–7.55 (m, 4H); ^{13}C NMR (126 MHz, $CDCl_3$) δ 73.9, 81.6, 121.8, 128.5, 129.2, 132.5; MS (EI): *m*/*z* (%) 202 (M$^+$, 100); HRMS (EI) calcd for $C_{16}H_{10}$: 202.0783, found: 202.0783.

Prop-1-yn-1-ylbenzene (4e). 4e: Yellow oil; IR (ATR) 2919, 2855, 1726, 1599, 1490, 1441, 1276, 754, 690 cm^{-1}; ^{1}H NMR (301 MHz, $CDCl_3$) δ 2.05 (s, 3H), 7.26–7.30 (m, 3H), 7.37–7.40 (m, 2H); ^{13}C NMR (126 MHz, $CDCl_3$) δ 4.4, 79.7, 85.8, 124.0, 127.5, 128.2, 131.5; MS (EI): *m*/*z* (%) 116 (M$^+$, 100); HRMS (EI) calcd for C_9H_8: 116.0626, found: 116.0626. [2-^{13}C]-**4e**: ^{1}H NMR (500 MHz, $CDCl_3$) δ 2.05 (d, J = 10.9 Hz, 3H), 7.24–7.29 (m, 3H), 7.38–7.39 (m, 2H); ^{13}C NMR (126 MHz, $CDCl_3$) δ 4.3 (d, J = 68.4 Hz), 79.6 (d, J = 180.5 Hz), 85.8 (^{13}C-labeled, J = 68.4, 180.5 Hz), 124.1

(d, J = 13.2 Hz), 127.5, 128.2, 131.5 (d, J = 2.4 Hz). Position of ^{13}C-labelled carbon was confirmed by hydogenation of 2-^{13}C-1-propyn-1-ylbenzene [2-^{13}C]-**4e** in the presence of Pd/C. 2-^{13}C-propylbenzene: ^{1}H NMR (500 MHz, $CDCl_3$) δ 0.94 (dt, J = 4.2, 7.4 Hz, 3H), 1.65 (d of sextet, J = 126.6, 7.4 Hz, 2H), 2.58 (dt, J = 4.2, 7.4 Hz, 2H), 7.15–7.18 (m, 3H), 7.24–7.28 (m, 2H); ^{13}C NMR (126 MHz, $CDCl_3$) δ 13.9 (d, J = 33.6 Hz), 24.6 (^{13}C-labeled, J = 33.6 Hz), 38.1 (J = 33.6 Hz), 125.6, 128.2, 128.5, 142.7.

Penta-1,3-diyn-1-ylbenzene (4f). iPrMgCl (2.5 equiv) and *t*-BuMgCl (2.5 equiv) were used. Colorless oil; IR (ATR) 3057, 2914, 2250, 1595, 1490, 1443, 1375, 1070, 1025, 916, 755, 689 cm^{-1}; ^{1}H NMR (399 MHz, $CDCl_3$) δ 2.02 (s, 3H), 7.27–7.35 (m, 3H), 7.46–7.48 (m, 2H); ^{13}C NMR (126 MHz, $CDCl_3$) δ 4.6, 64.3, 74.2, 74.4, 80.4, 122.1, 128.4, 128.8, 132.5; MS (EI): *m*/*z* (%) 140 (M^+, 100); HRMS (EI) calcd for $C_{11}H_8$: 140.0626, found: 140.0625.

1-Chloro-4-ethynylbenzene (4g). Yellow solid; mp 43.2–43.8 °C; IR (ATR) 3263, 1591, 1487, 1397, 1088, 1014, 823 cm^{-1}; ^{1}H NMR (400 MHz, $CDCl_3$) δ 3.11 (s, 1H), 7.30 (d, J = 8.8 Hz, 2H), 7.42 (d, J = 8.8 Hz, 2H); ^{13}C NMR (126 MHz, $CDCl_3$) δ 78.2, 82.5, 120.6, 128.7, 133.4, 134.9; MS (EI): *m*/*z* (%) 136 (M^+, 100), 101 (19); HRMS (EI) calcd for $C_8H_5{}^{35}Cl$: 136.0080, found: 136.0080.

^{1}H NMR

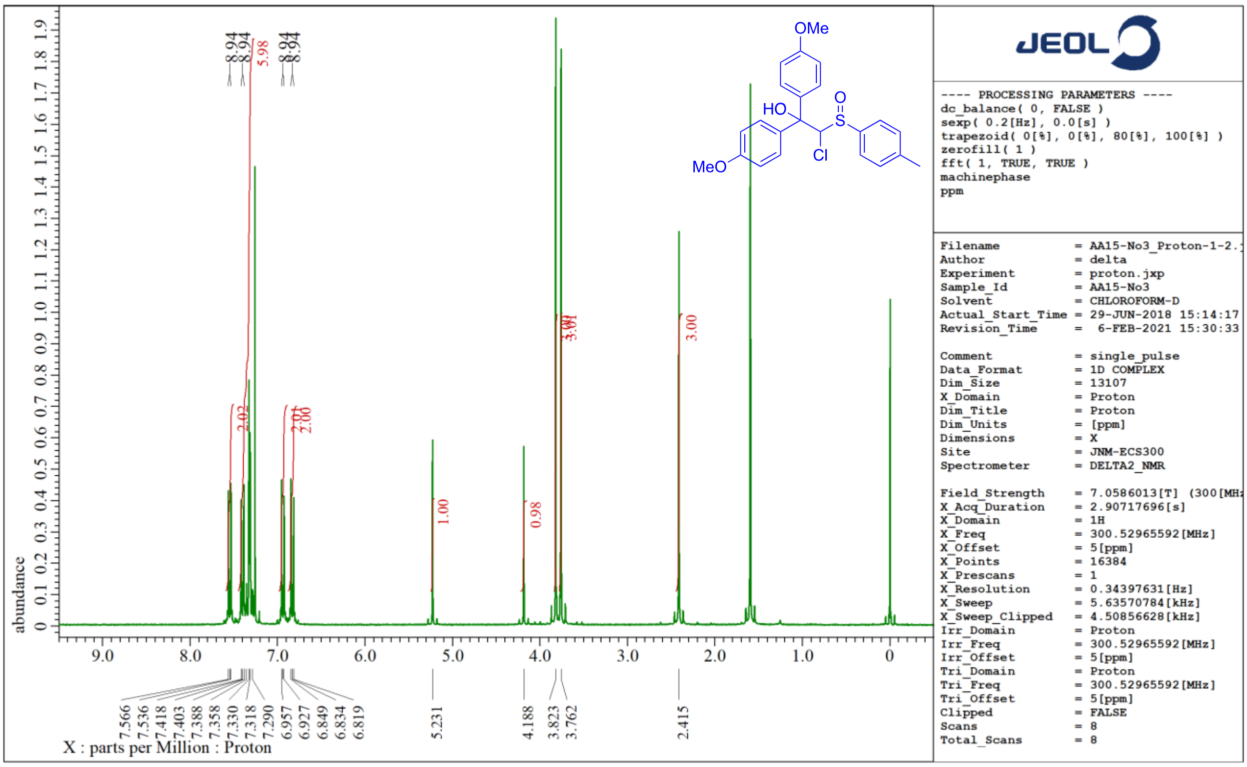

^{13}C NMR

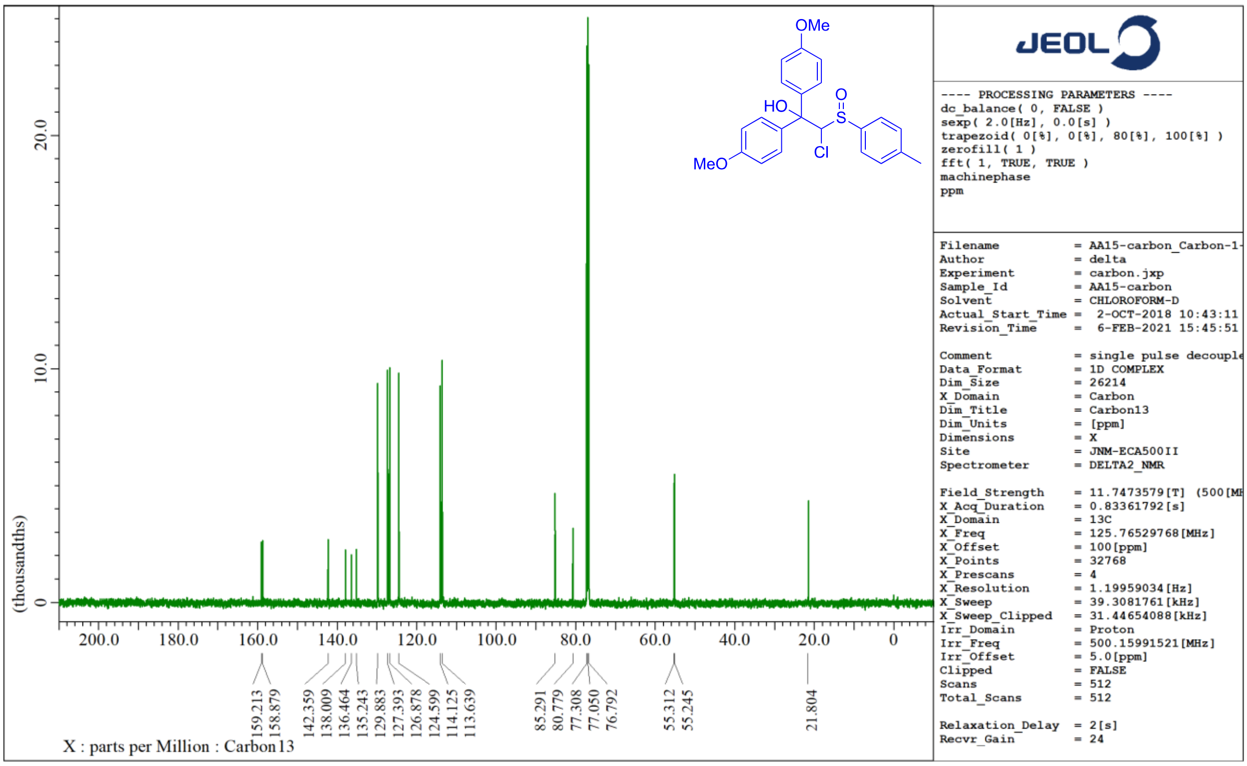

^{1}H NMR of **2a**

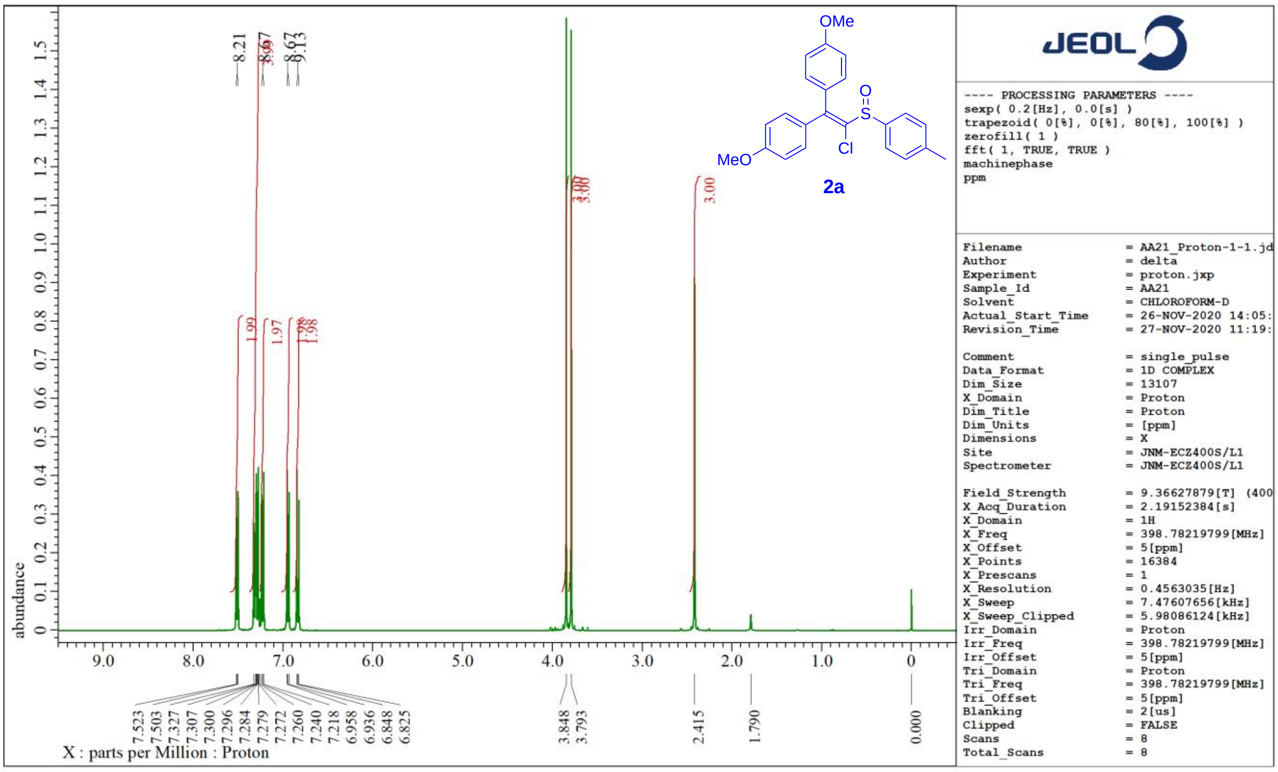

^{13}C NMR of **2a**

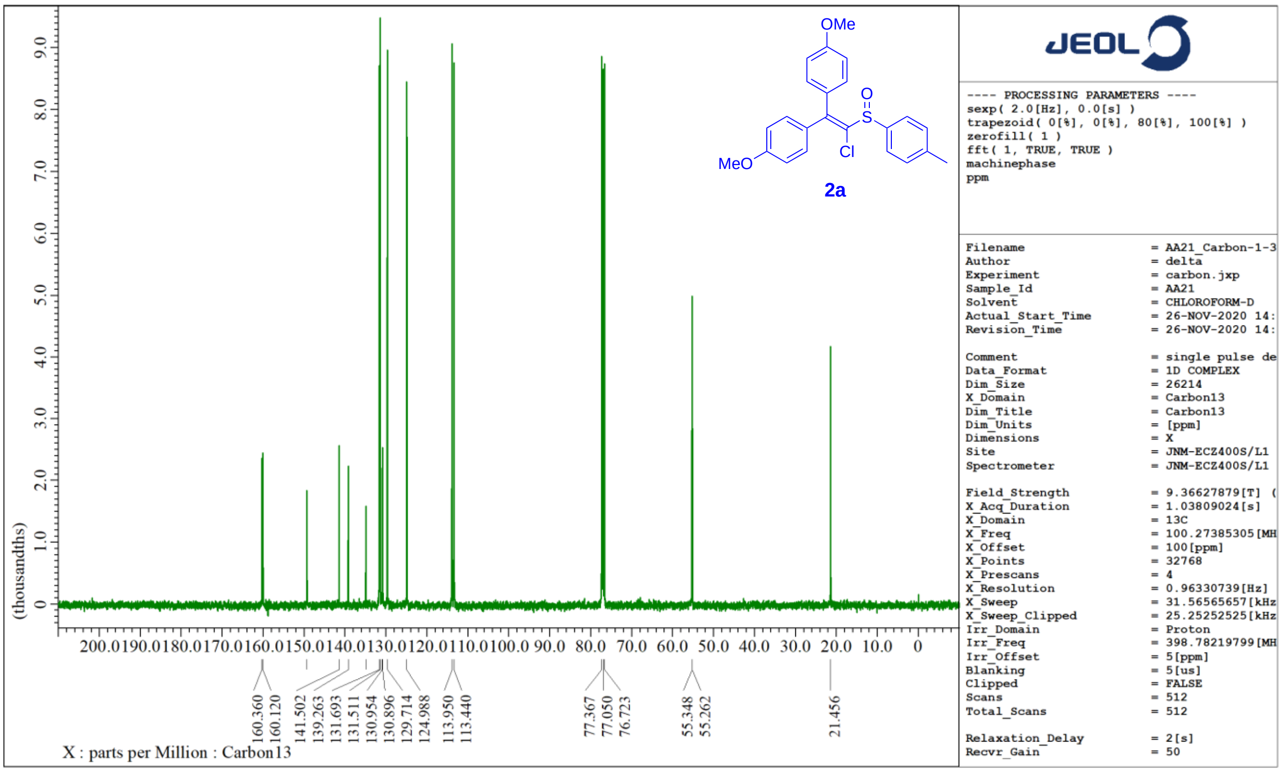

^{1}H NMR of **5**

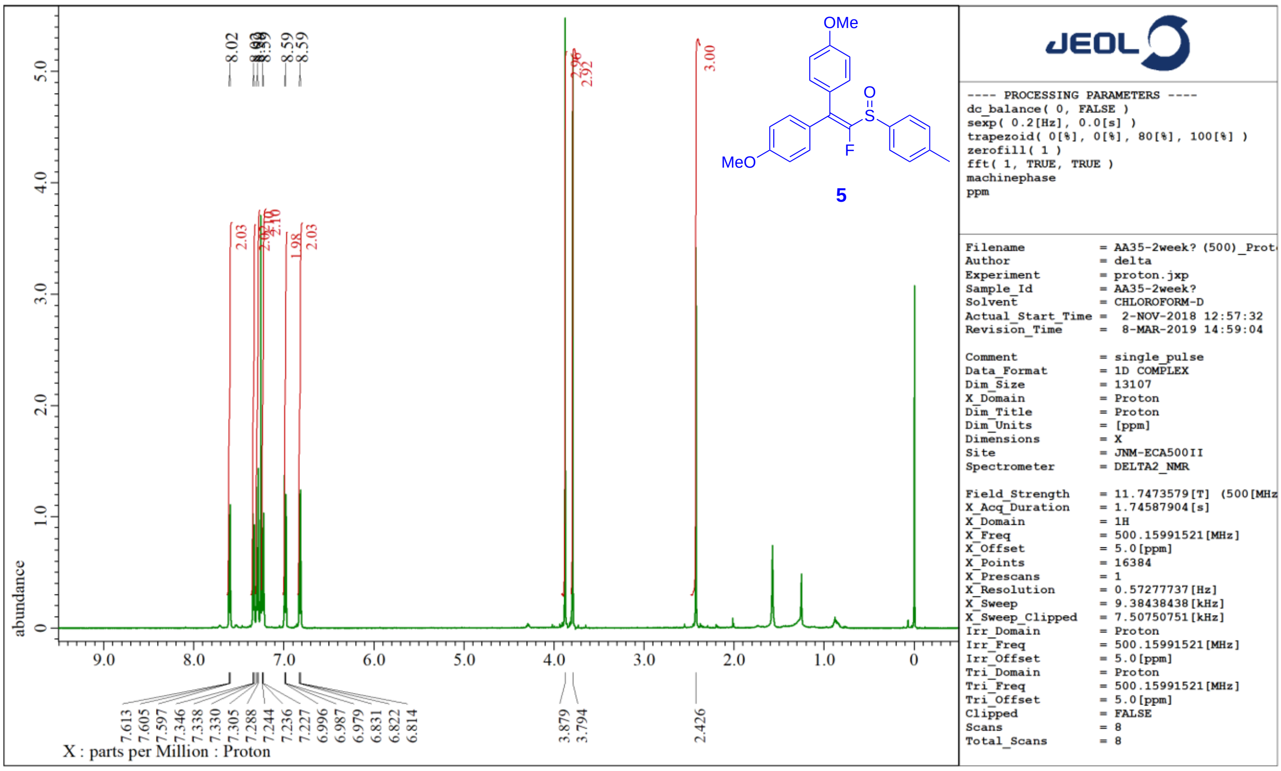

^{13}C NMR of **5**

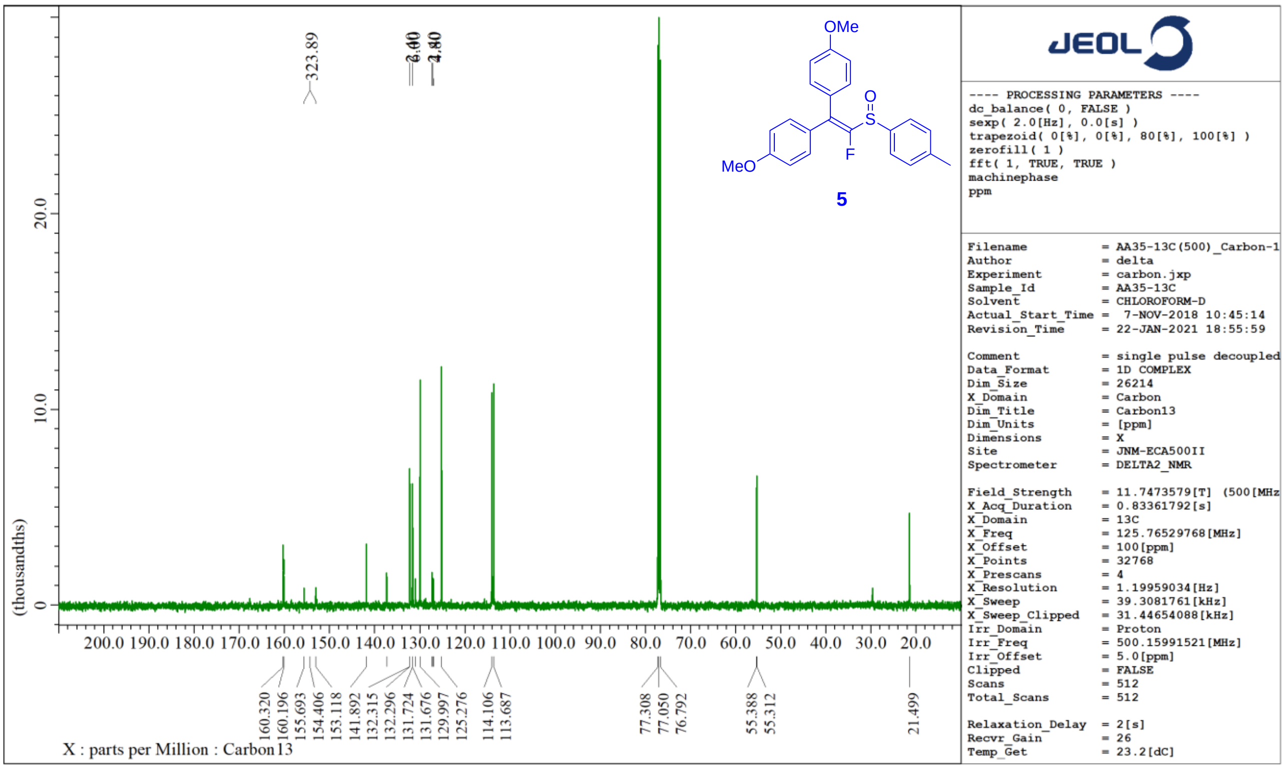

^{1}H NMR of **6**

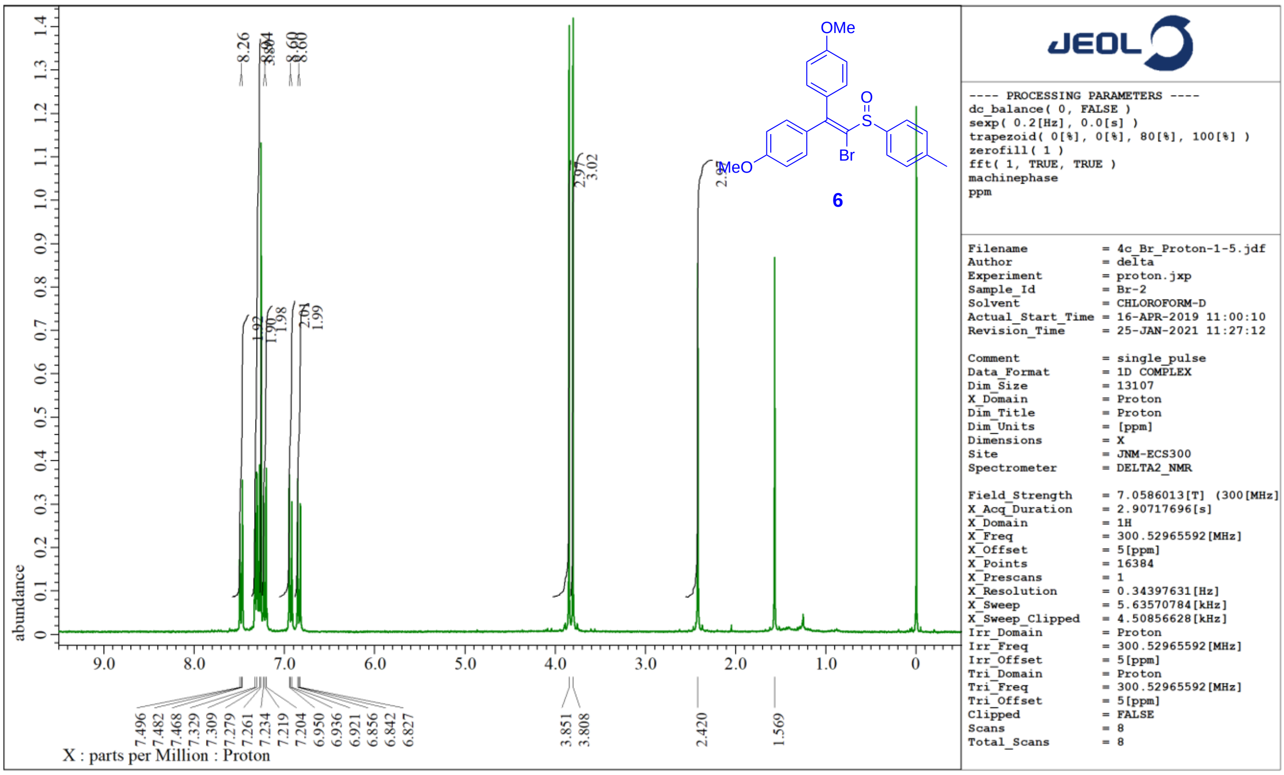

^{13}C NMR of **6**

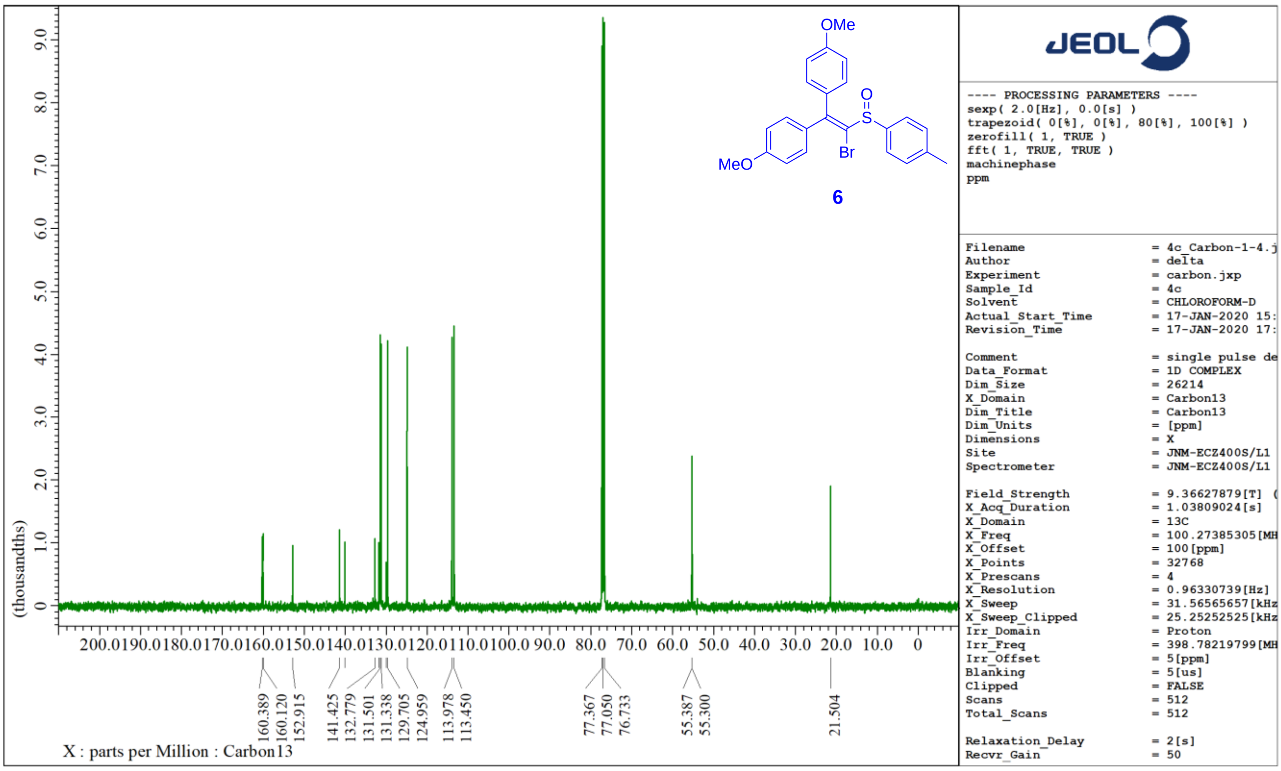

^{1}H NMR of **7**

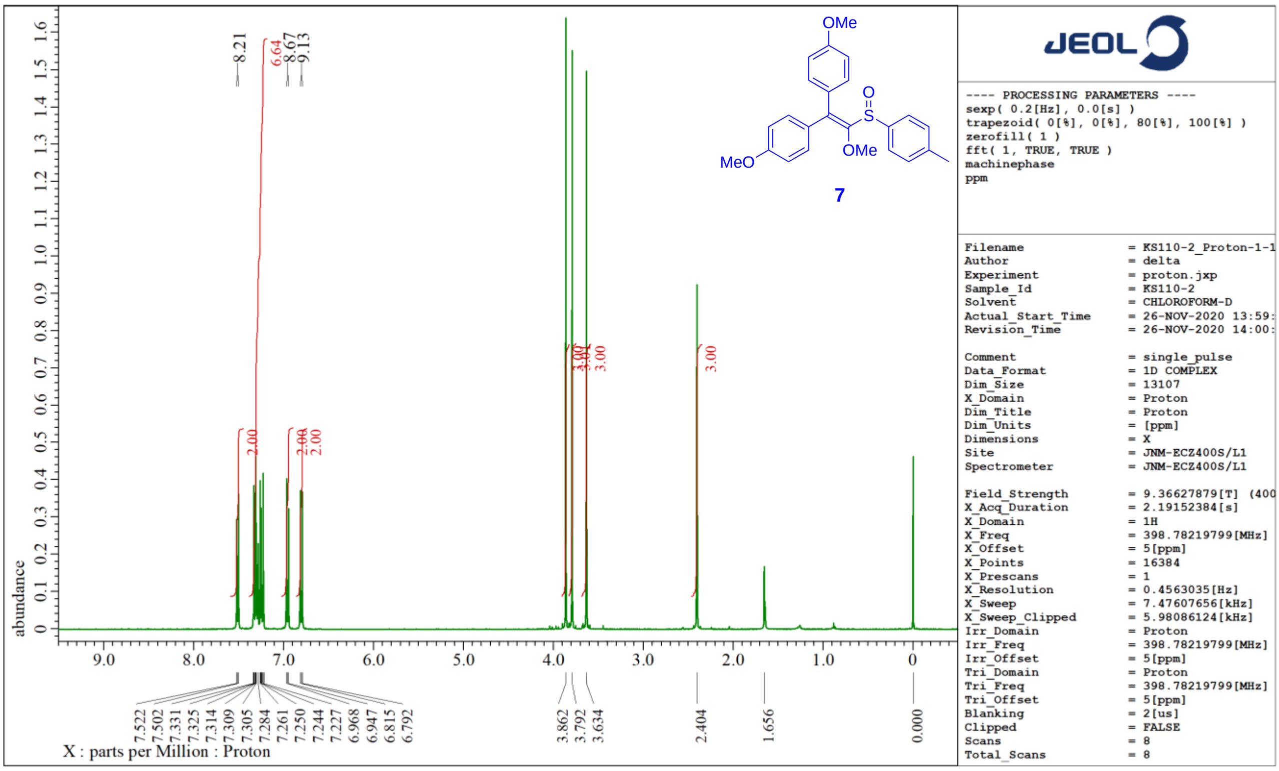

^{13}C NMR of **7**

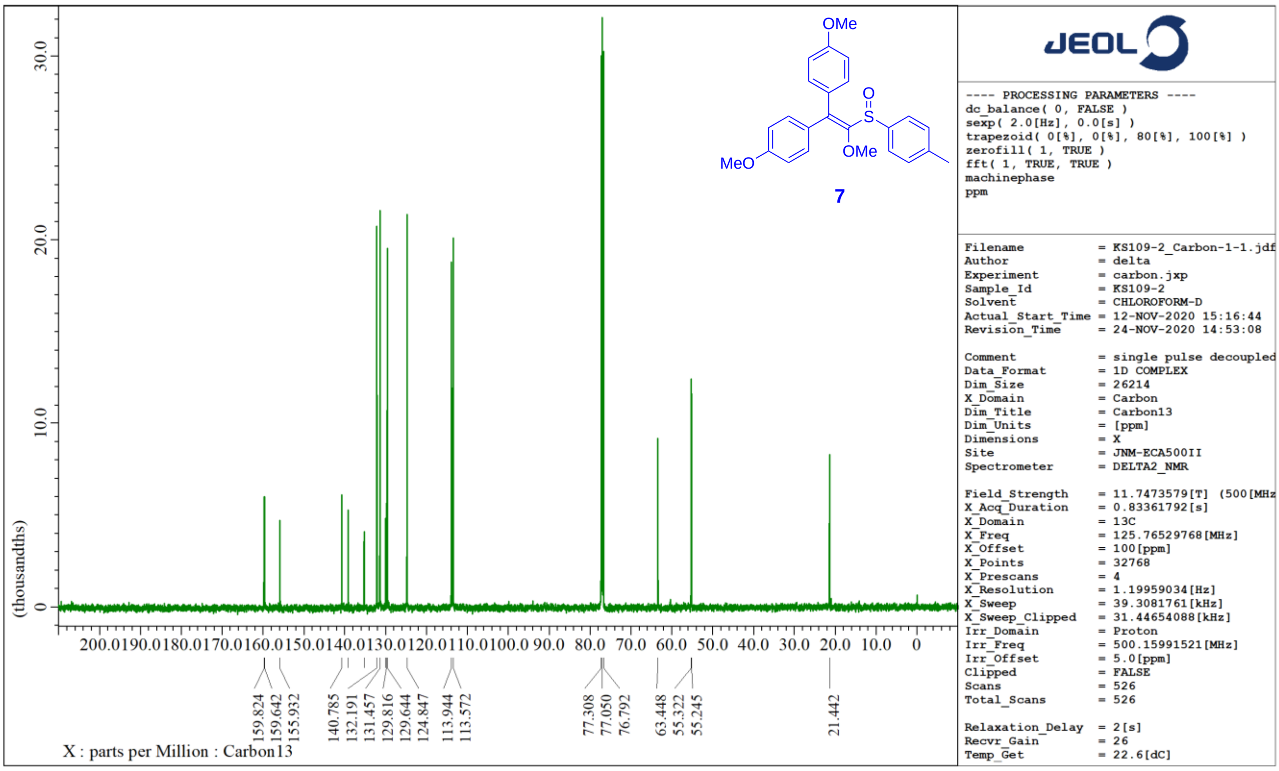

^{1}H NMR of **8**

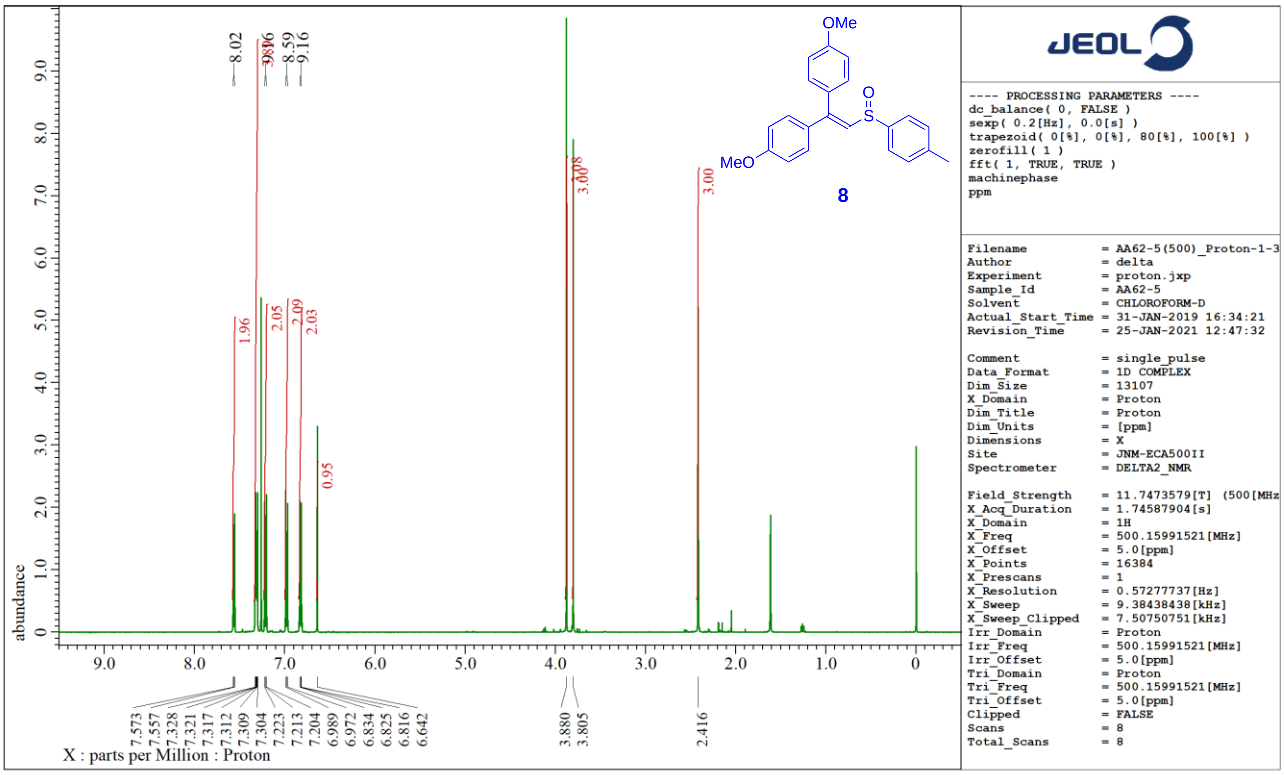

^{13}C NMR of **8**

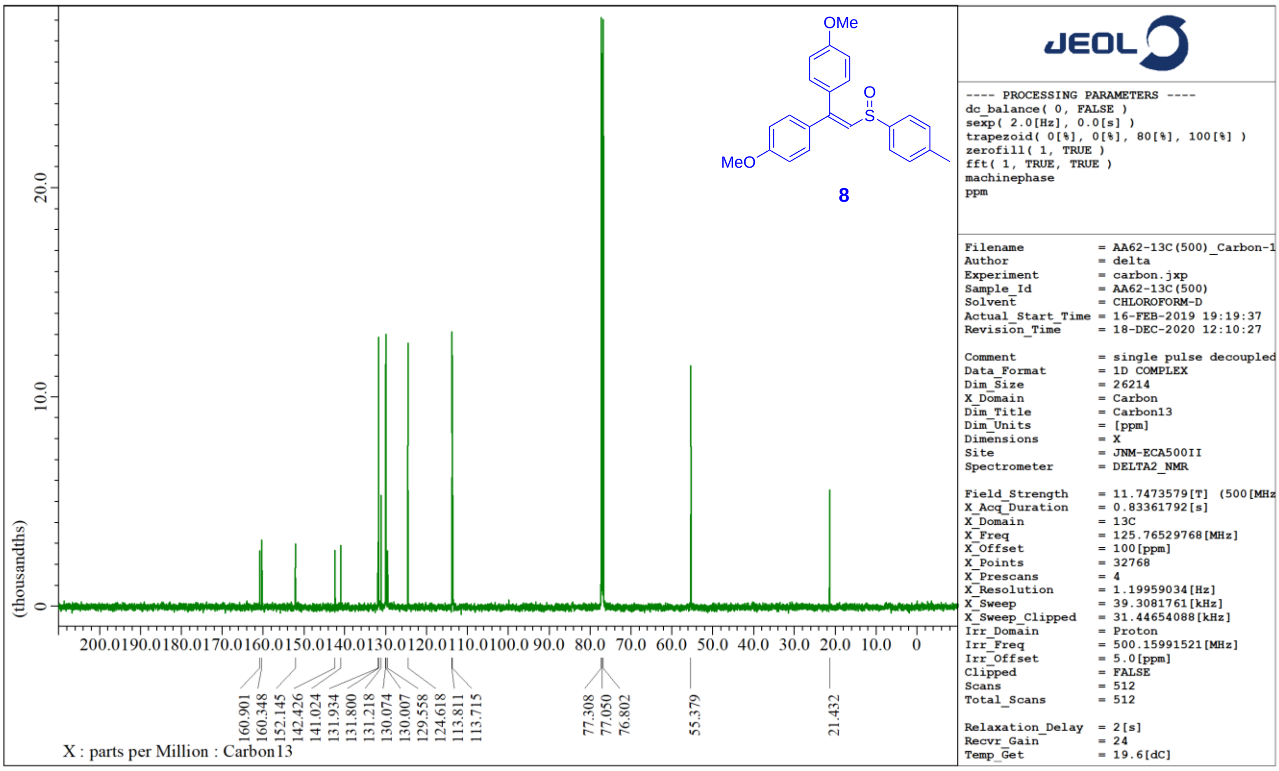

^{1}H NMR of **2b**

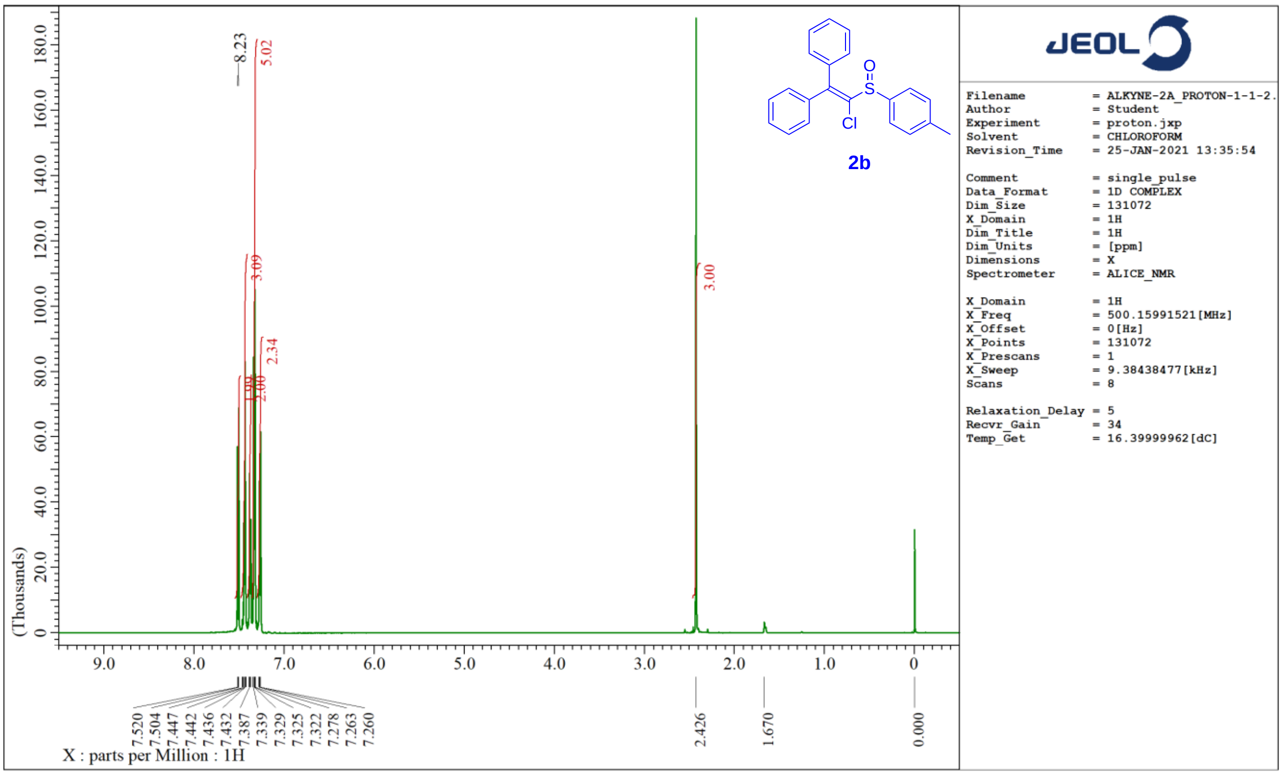

^{13}C NMR of **2b**

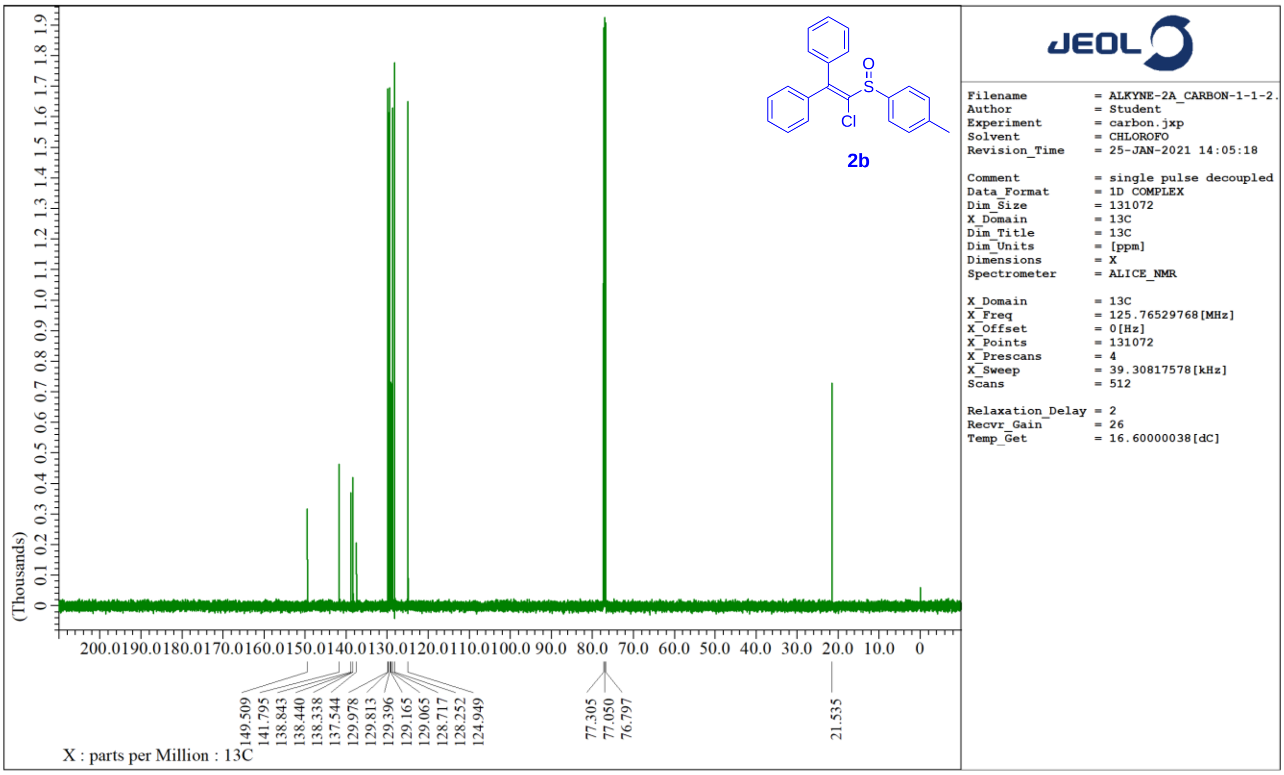

^{1}H NMR of **2c**

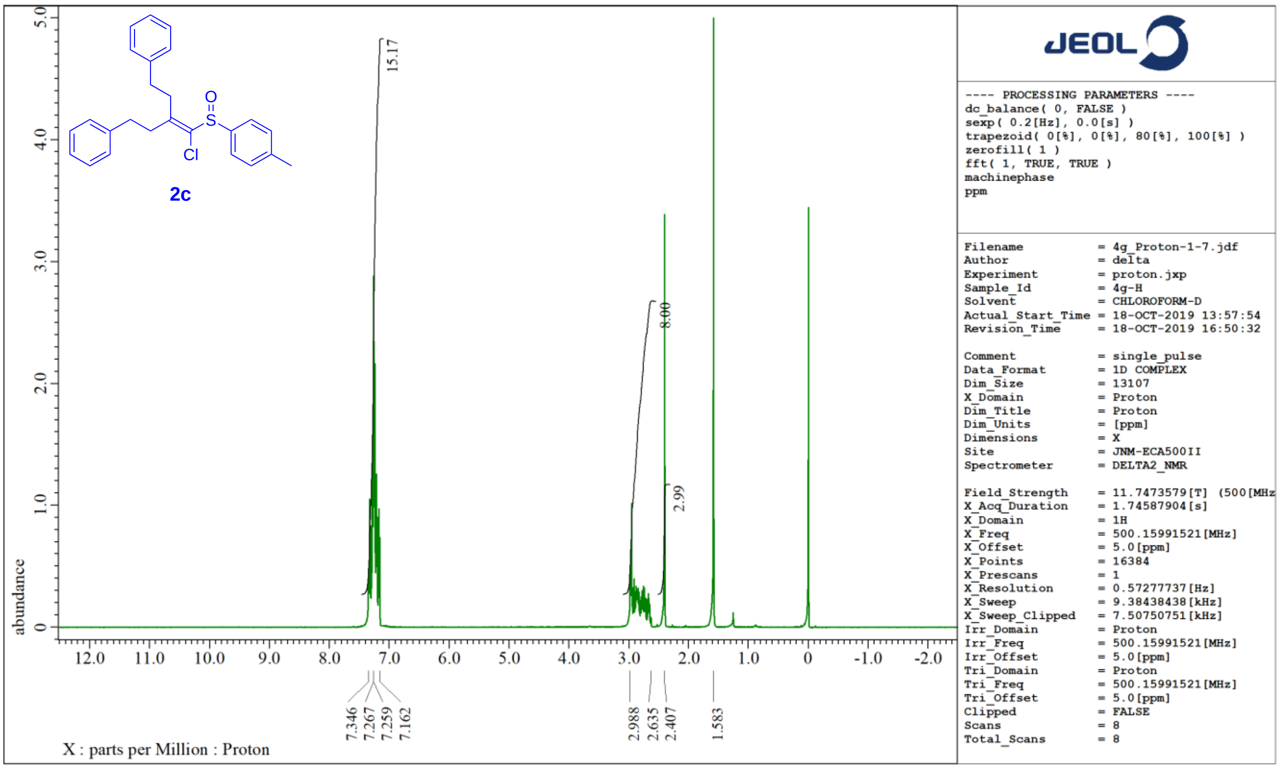

^{13}C NMR of **2c**

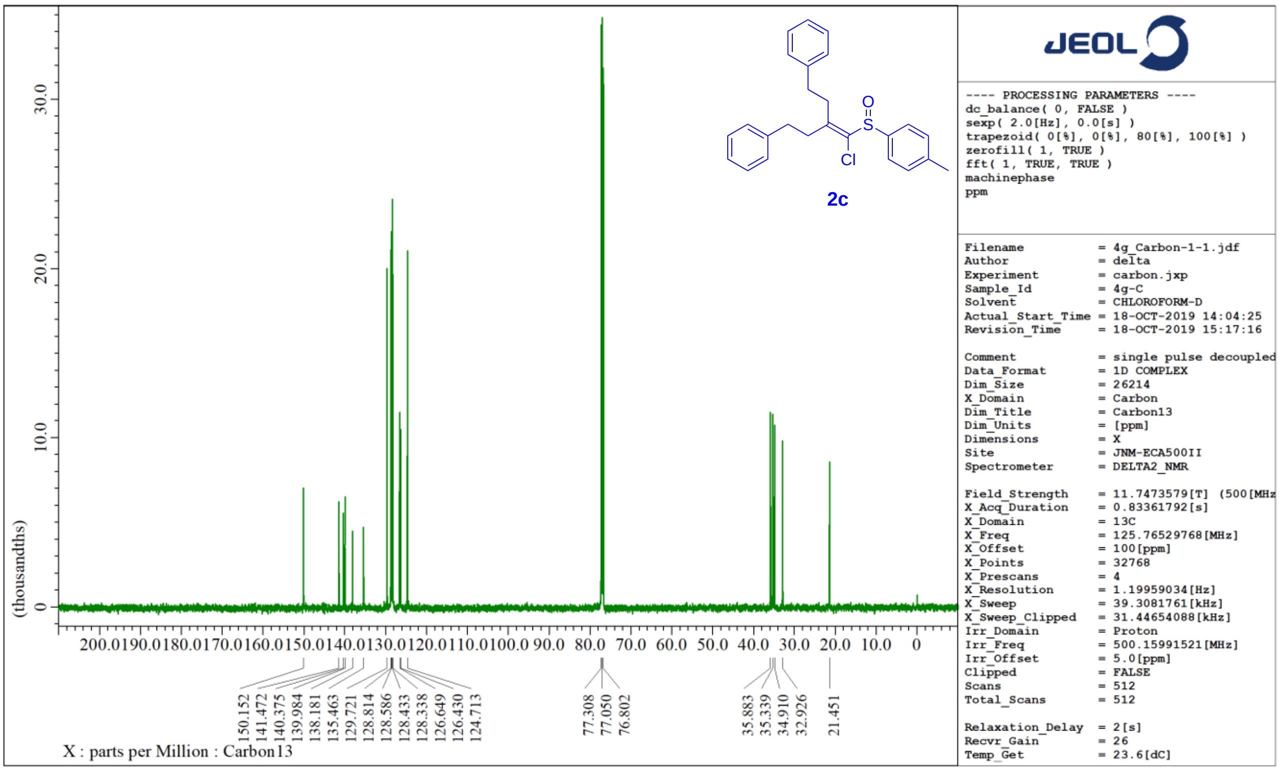

^{1}H NMR of *L*-**2d**

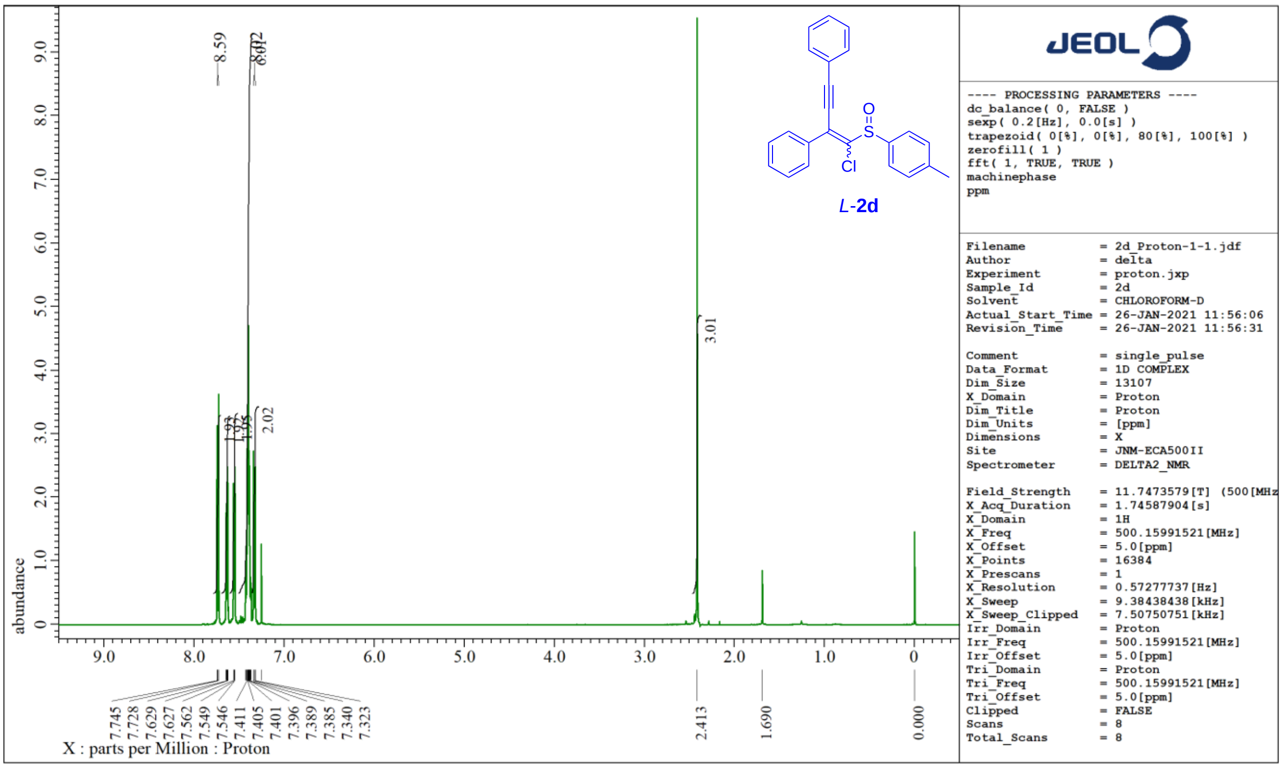

^{13}C NMR of *L*-**2d**

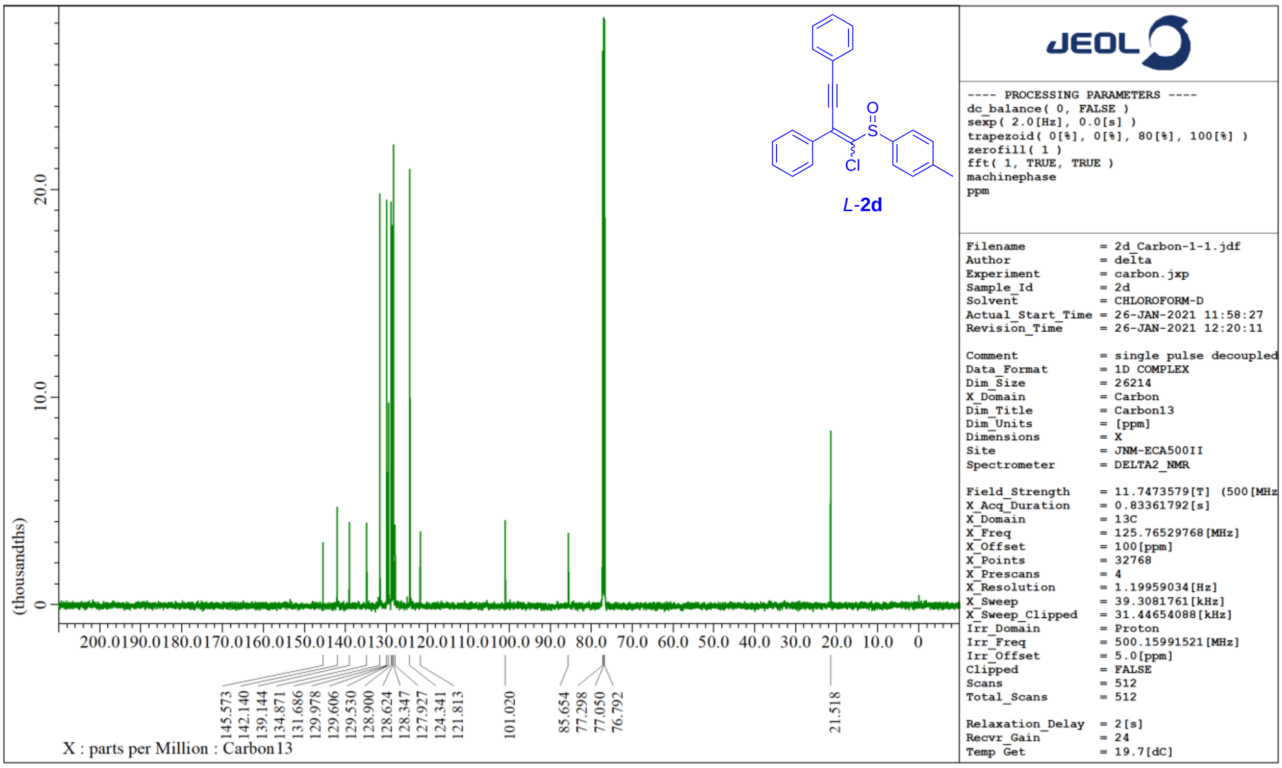

^{1}H NMR of *P*-**2d**

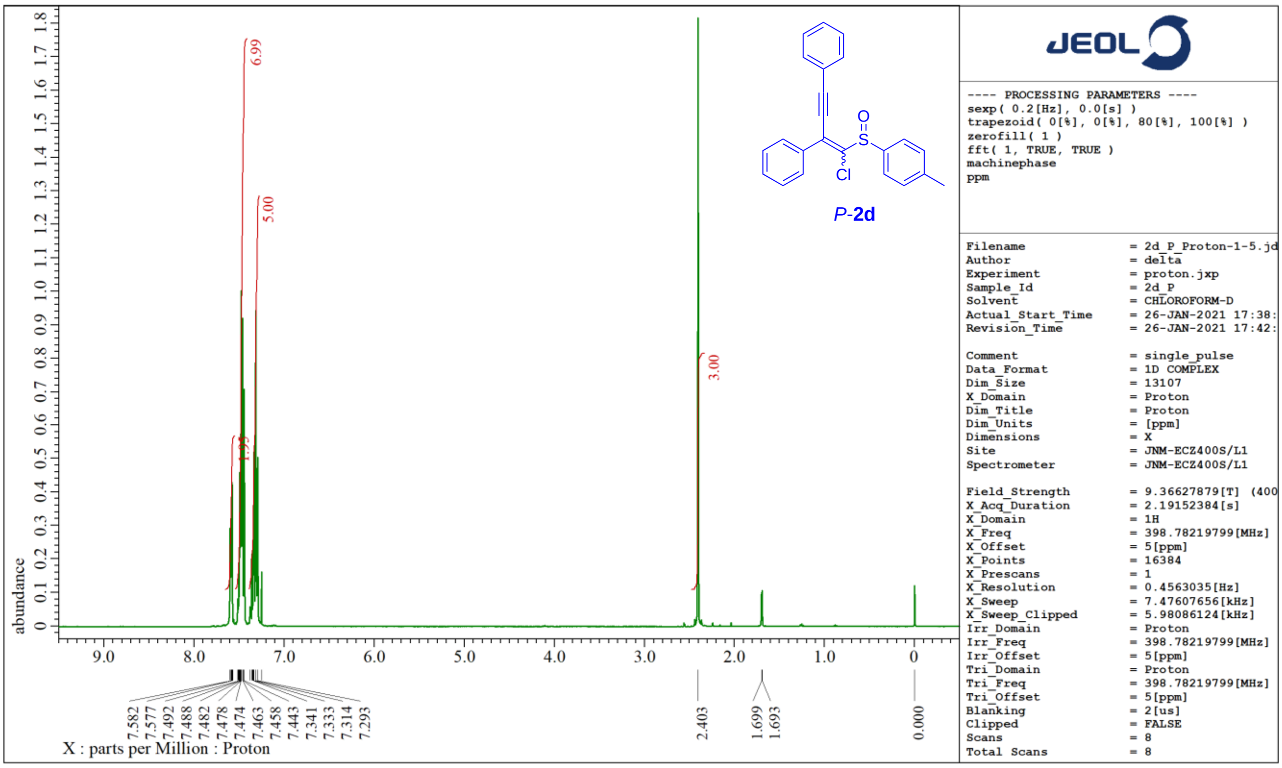

^{13}C NMR of *P*-**2d**

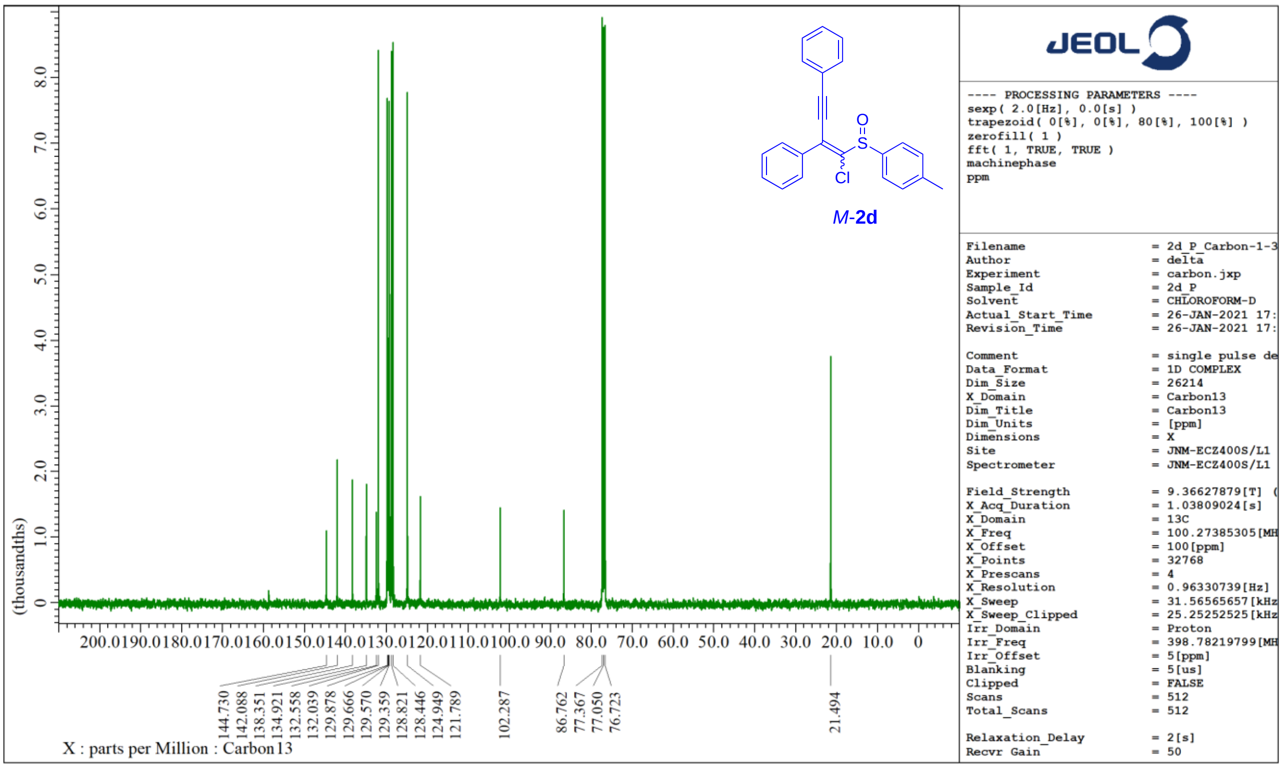

^{1}H NMR of (*E*)-**2e**

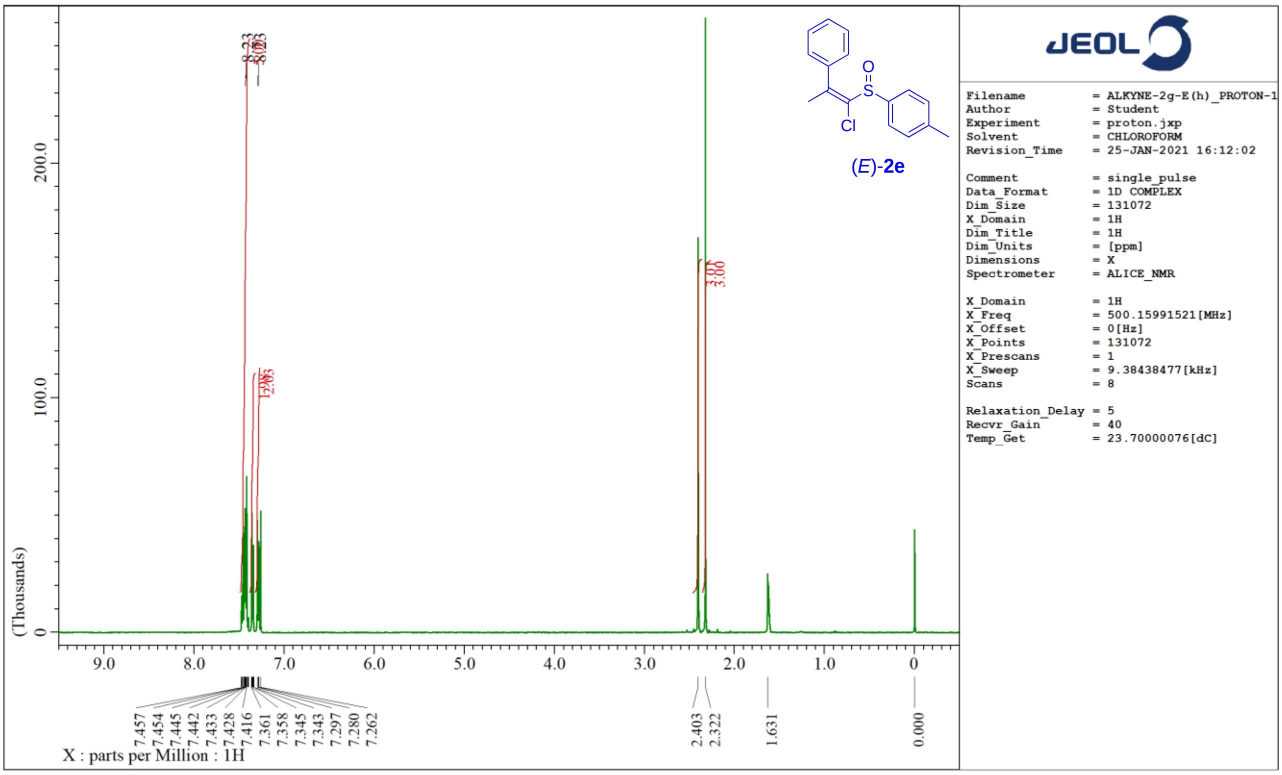

^{13}C NMR of (*E*)-**2e**

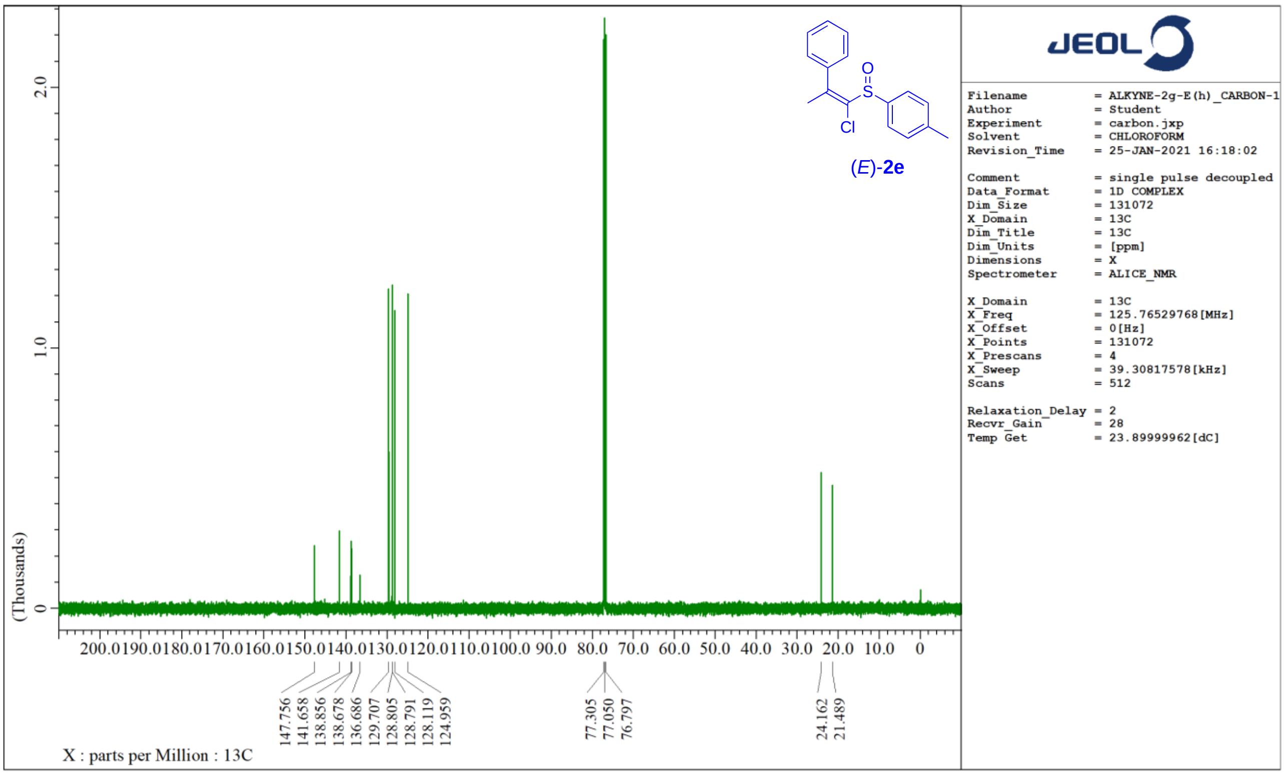

^{1}H NMR of [^{13}C]-(*E*)-**2e**

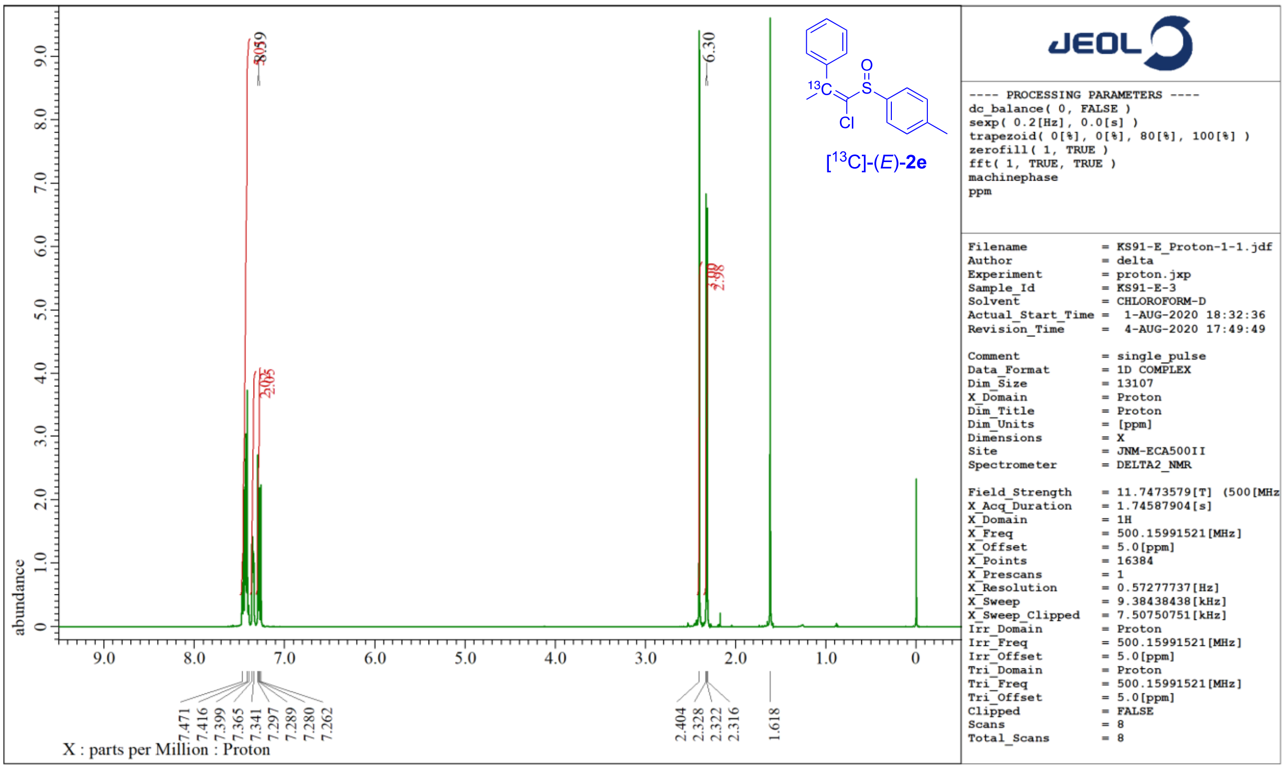

^{13}C NMR of [^{13}C]-(*E*)-**2e**

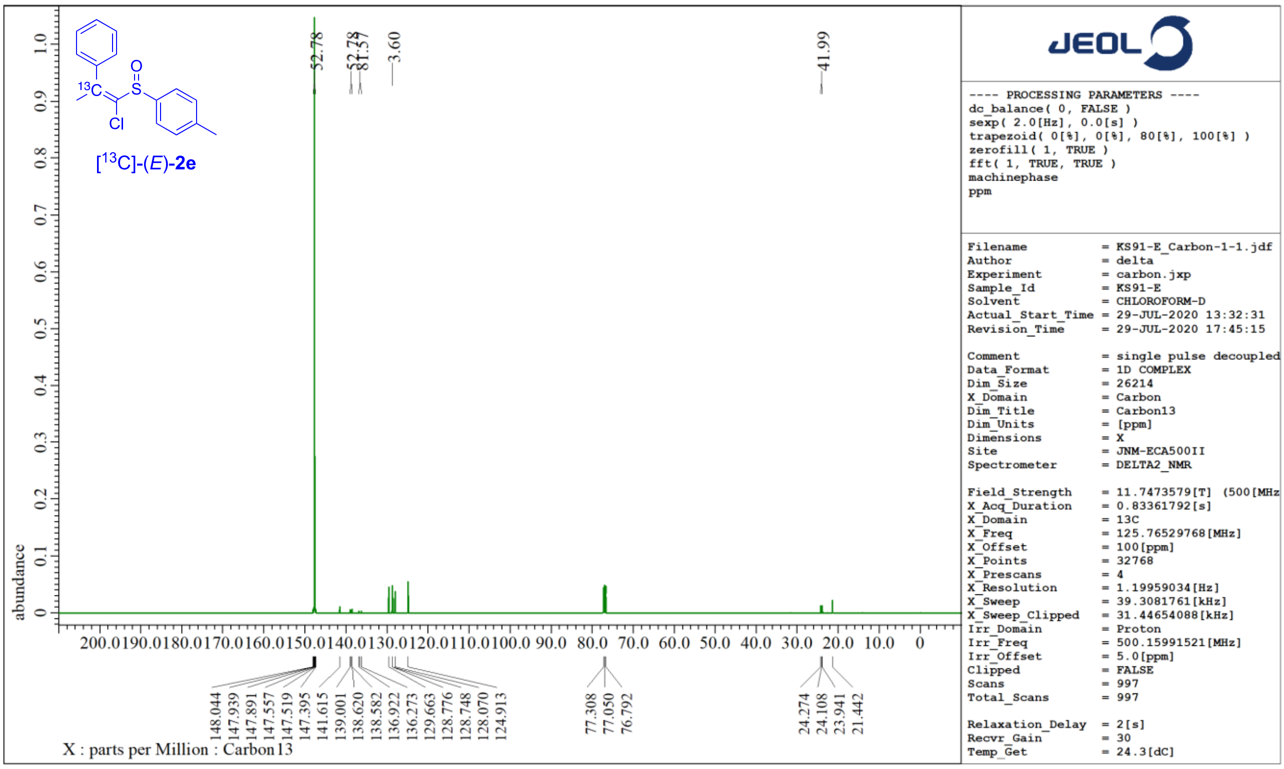

^{1}H NMR of (*Z*)-**2e**

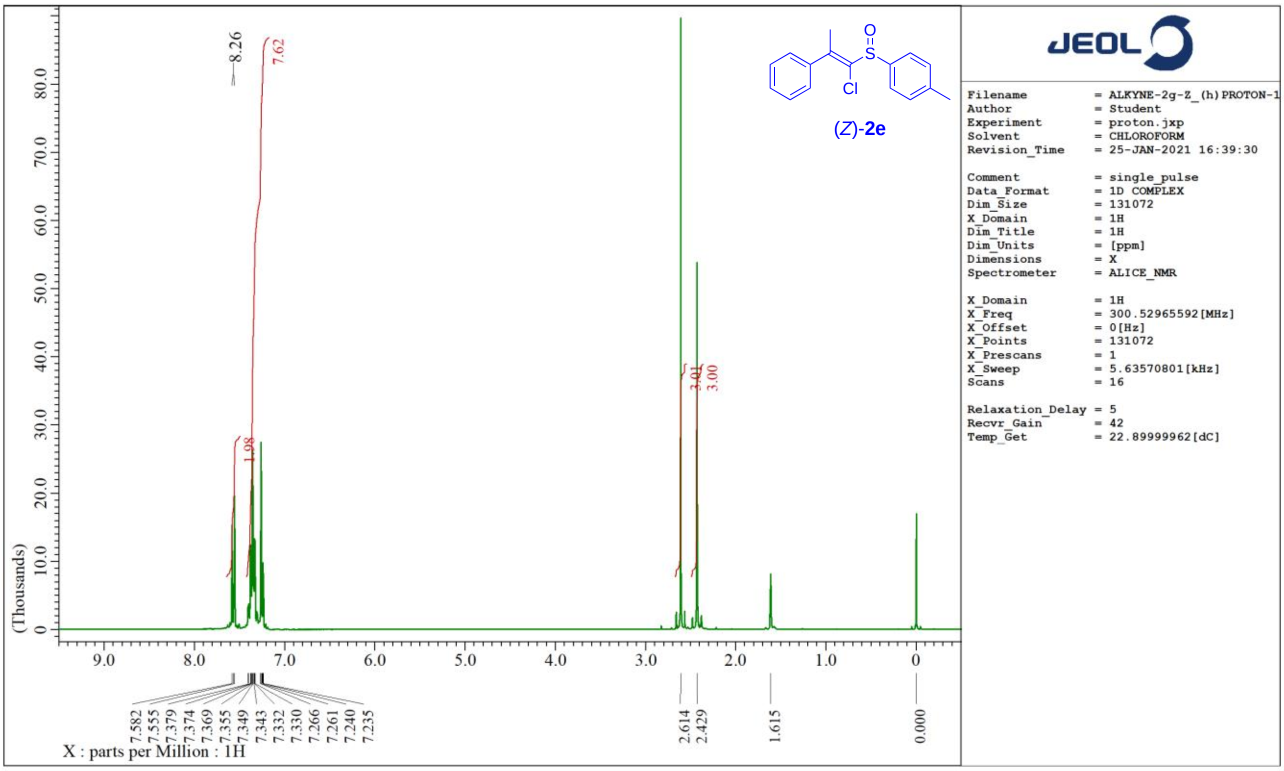

^{13}C NMR of (*Z*)-**2e**

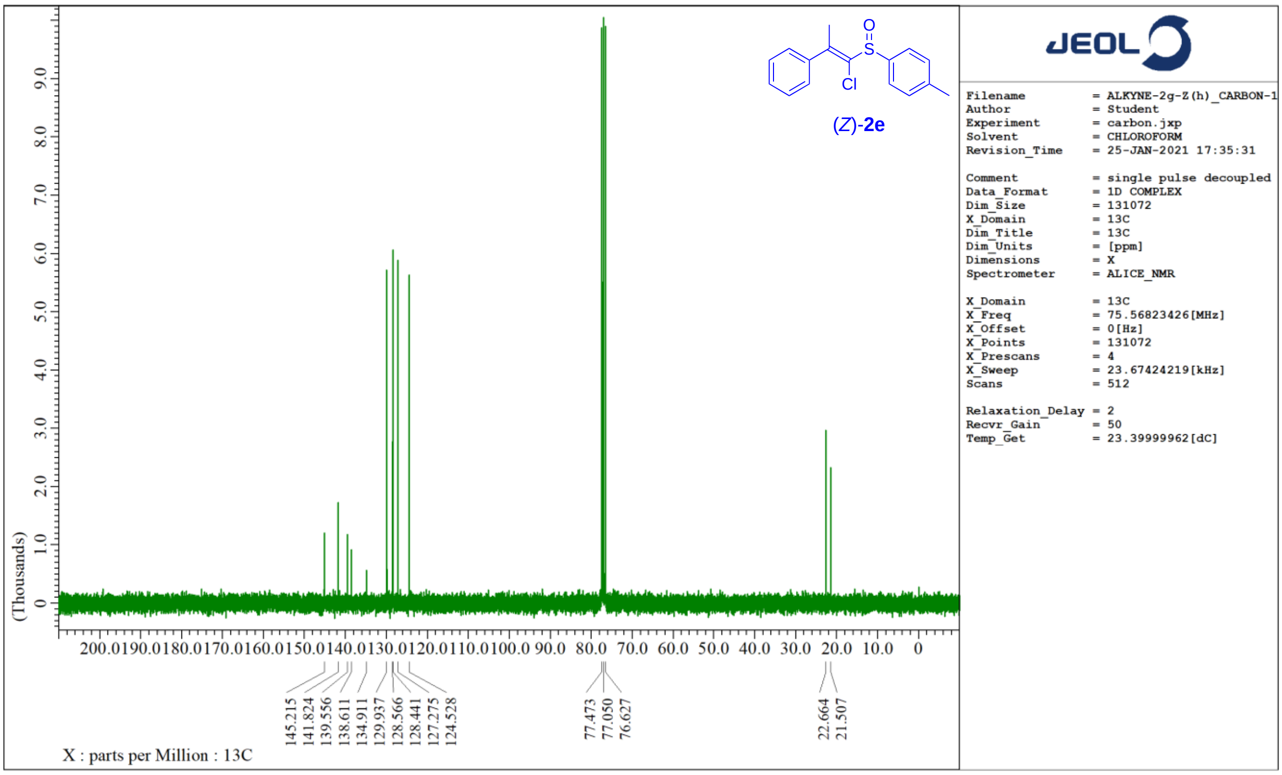

^{1}H NMR of [^{13}C]-(*Z*)-**2e**

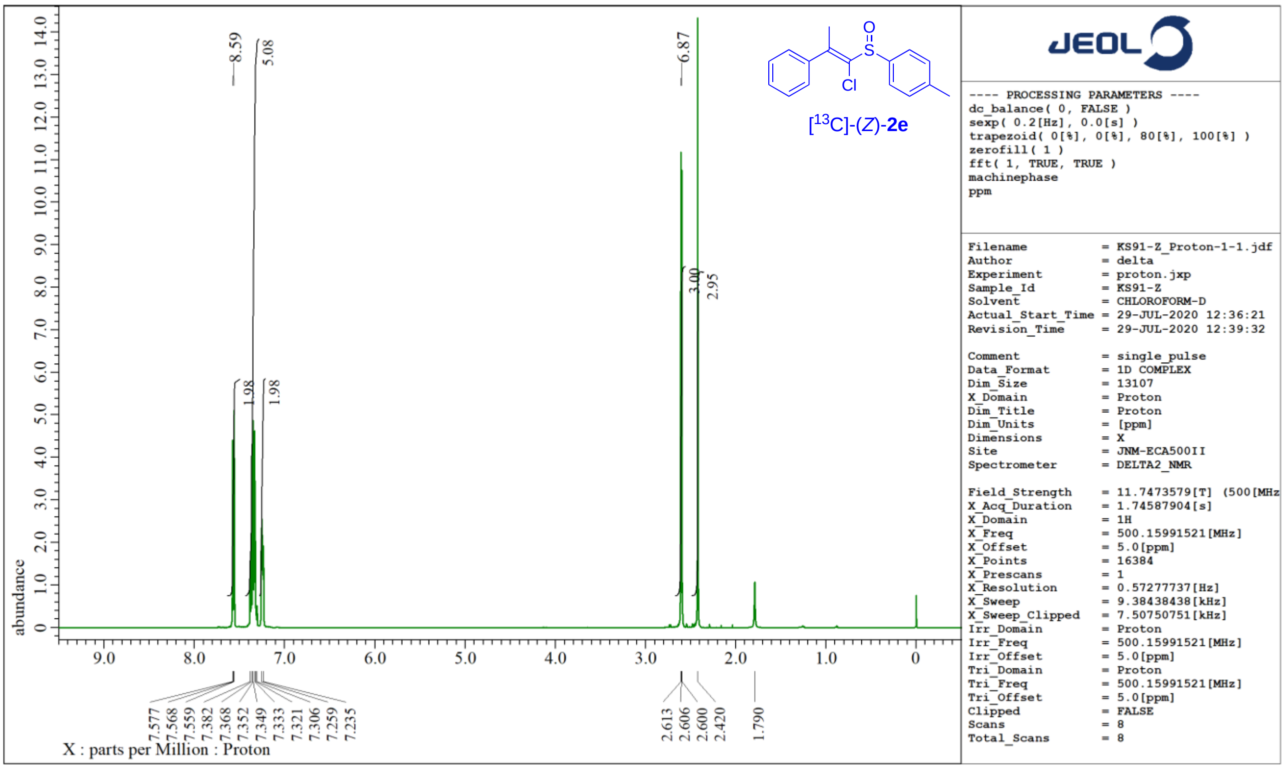

^{13}C NMR of [^{13}C]-(*Z*)-**2e**

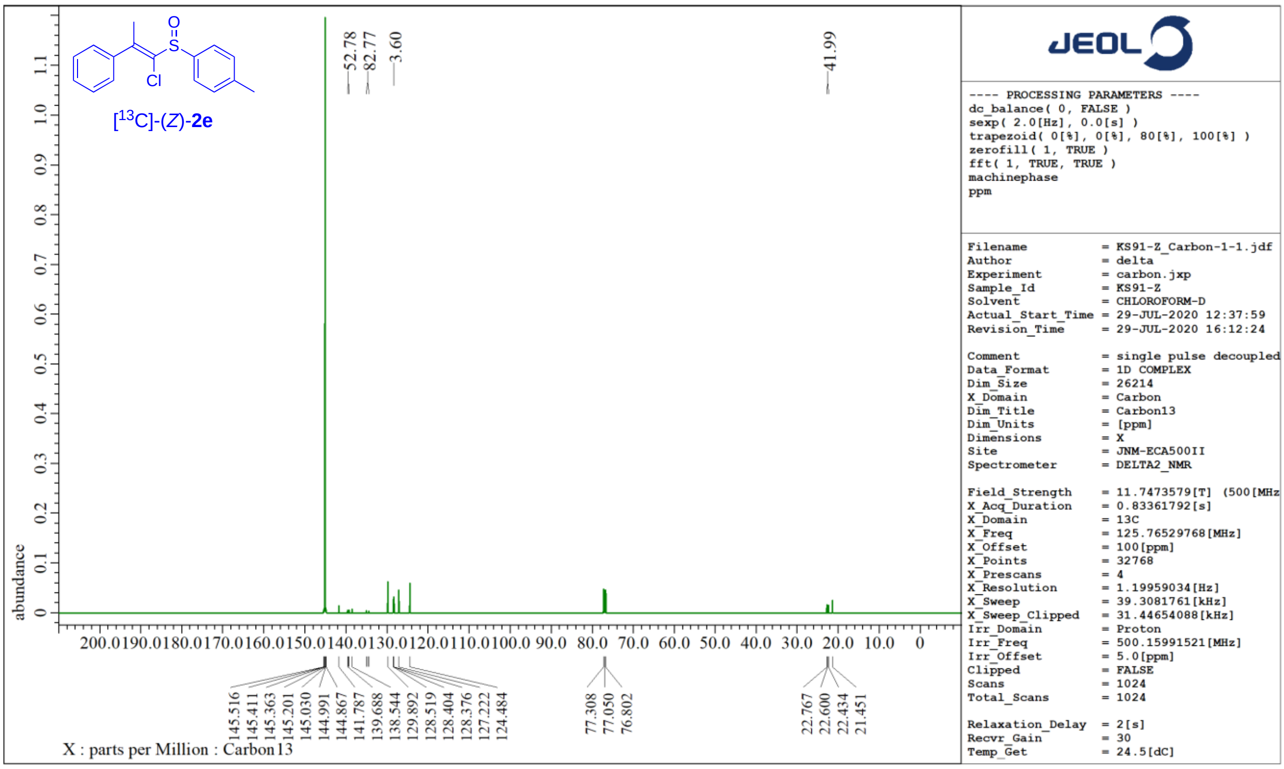

^{1}H NMR of (*E*)-**2f**

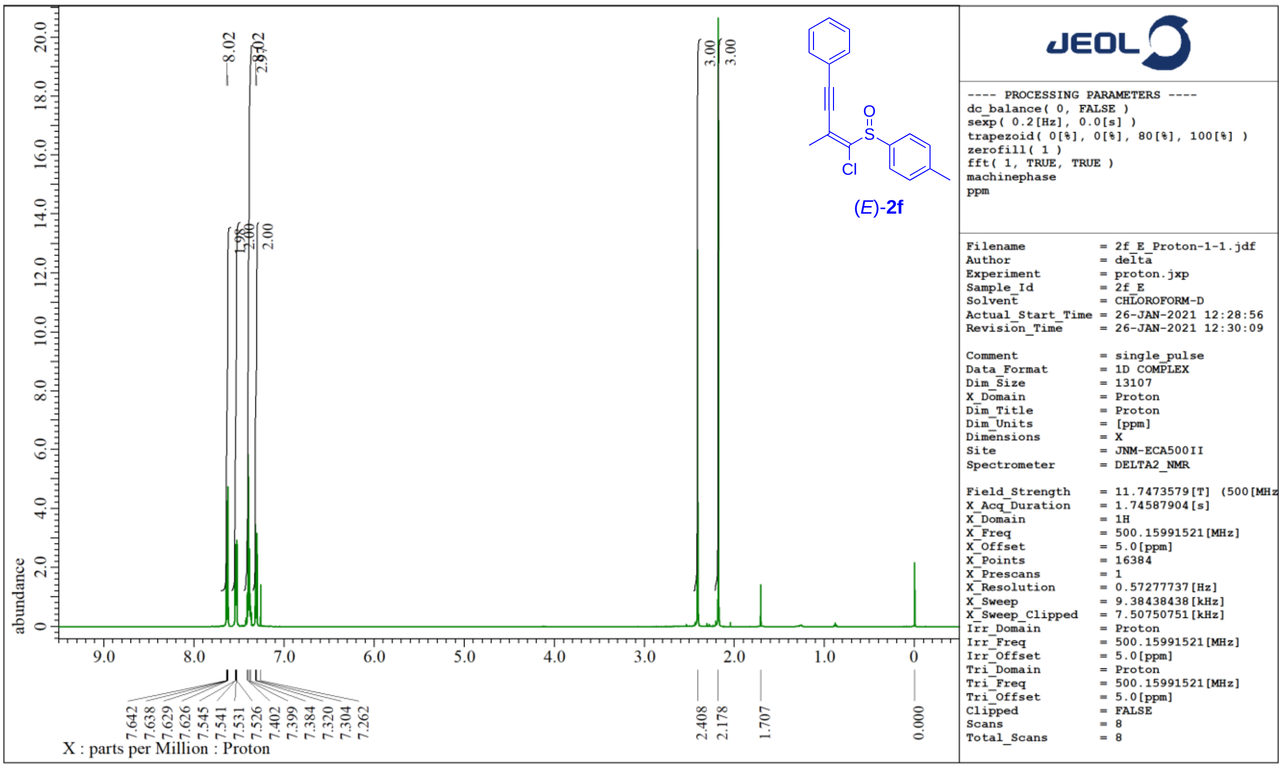

^{13}C NMR of (*E*)-**2f**

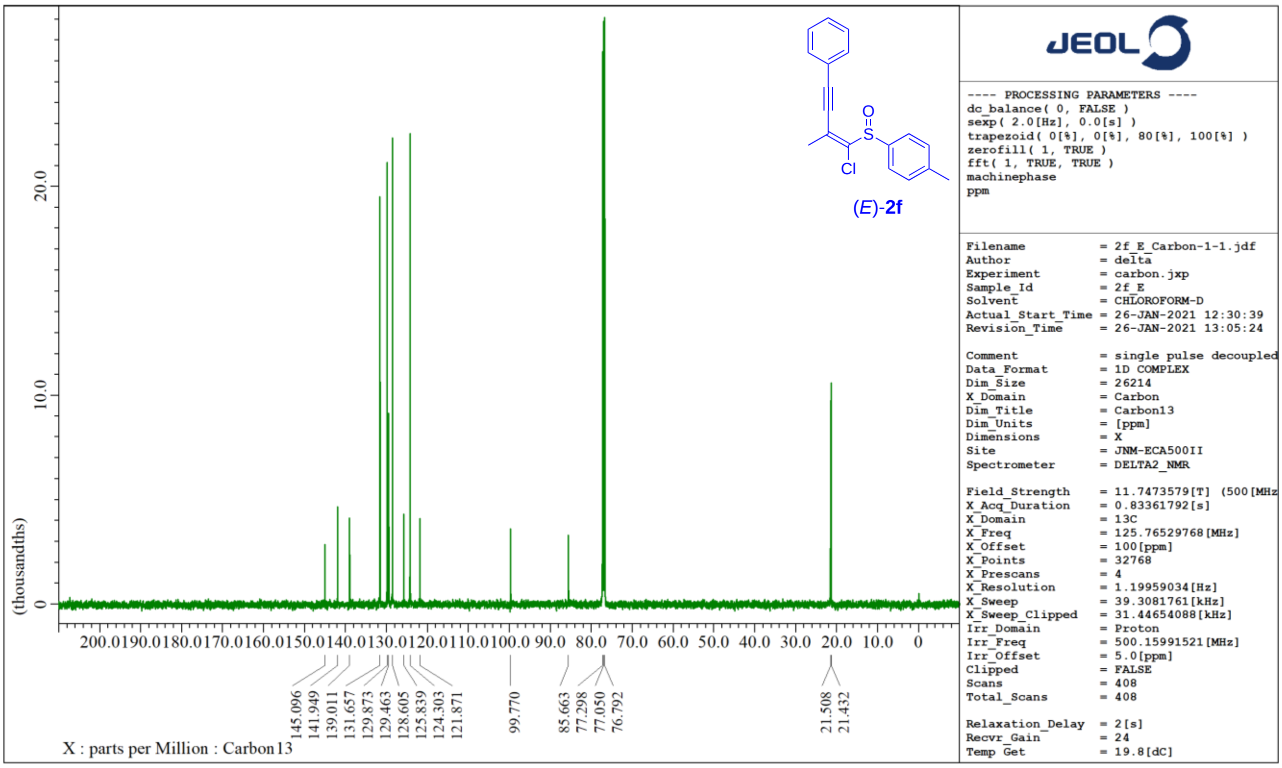

^{1}H NMR of (*Z*)-**2f**

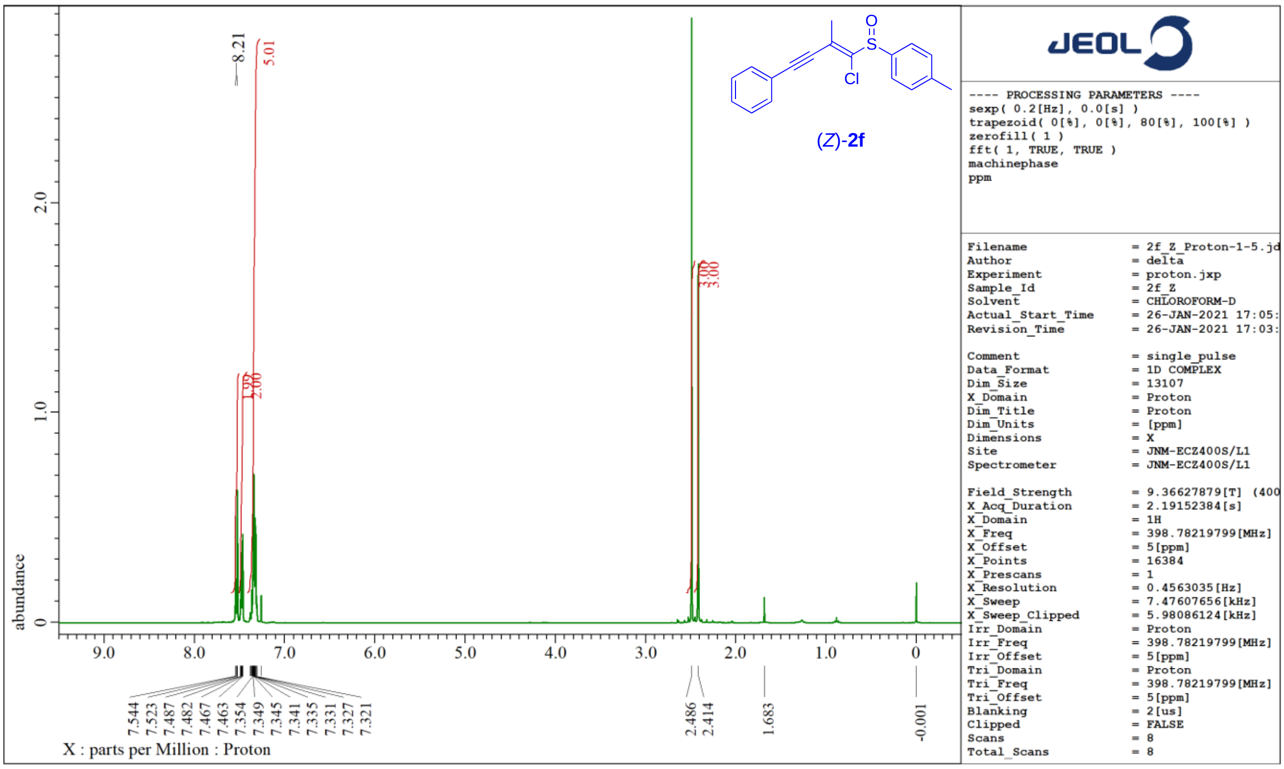

^{13}C NMR of (*Z*)-**2f**

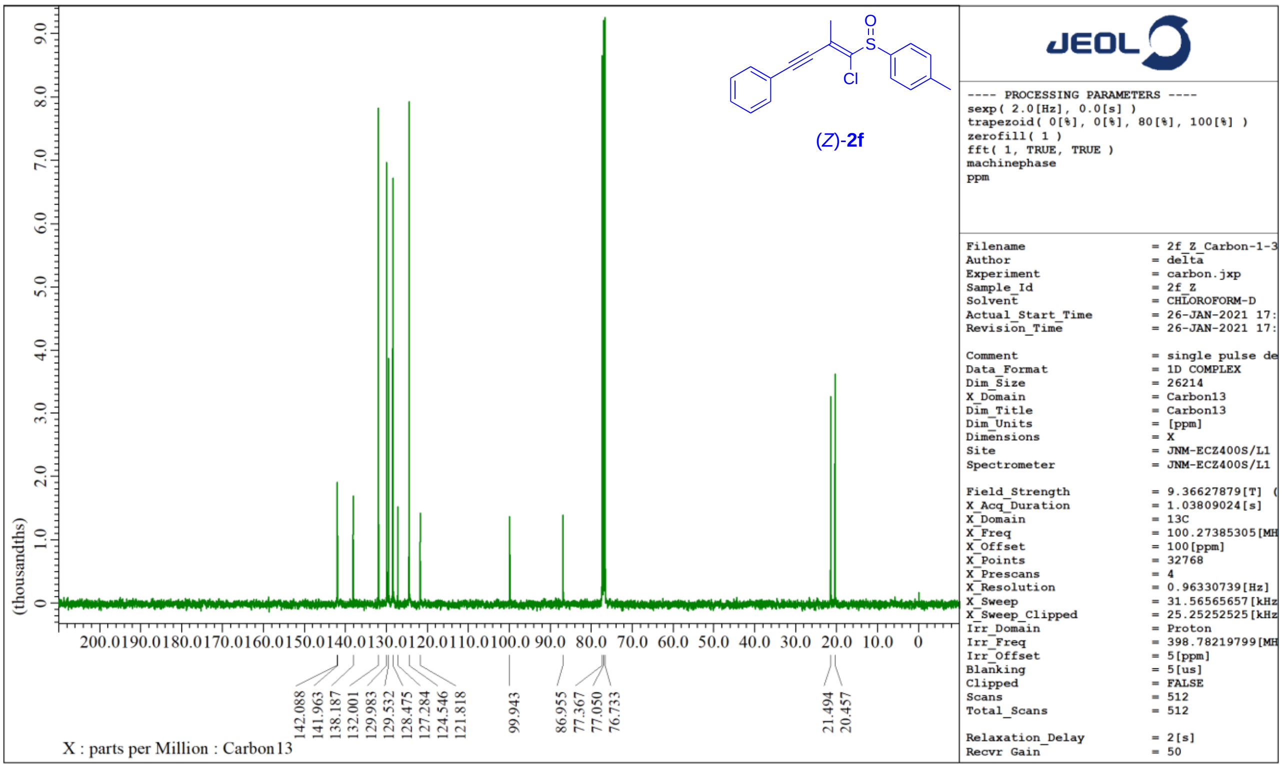

^{1}H NMR of (*E*)-**2g**

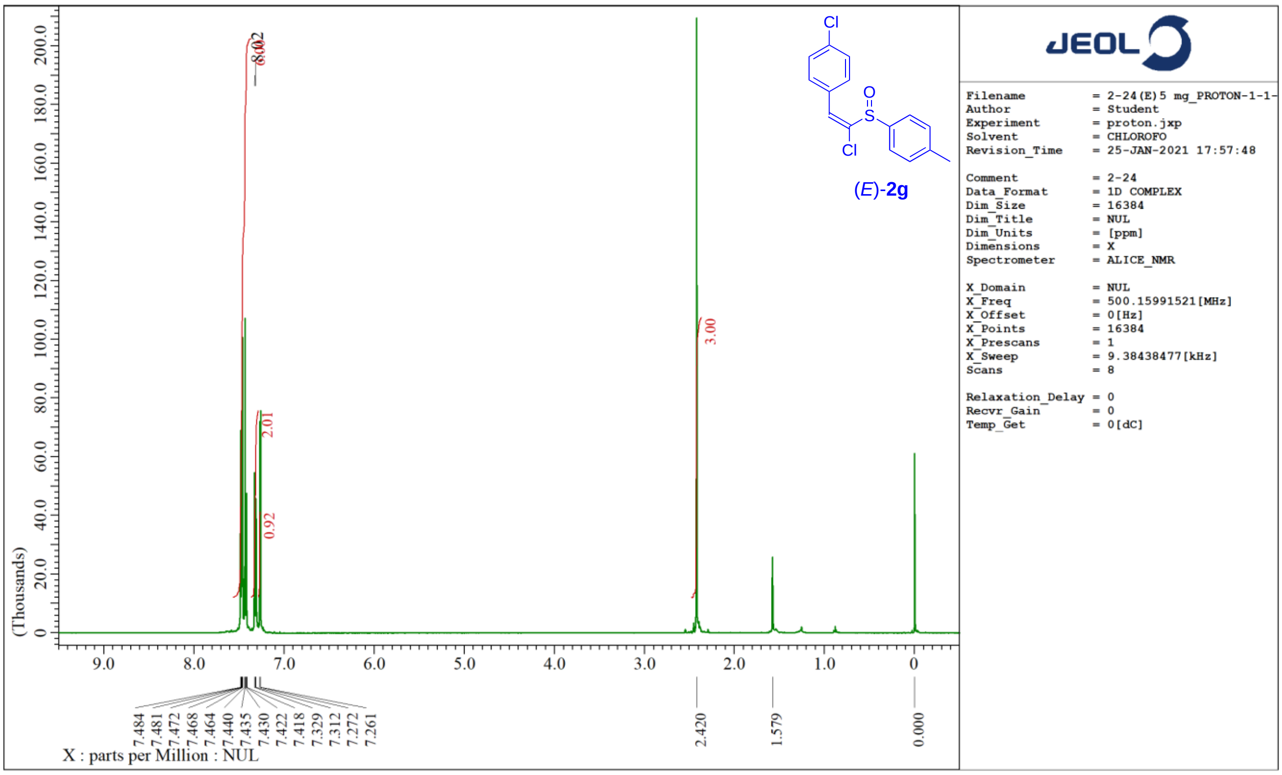

^{13}C NMR of (*E*)-**2g**

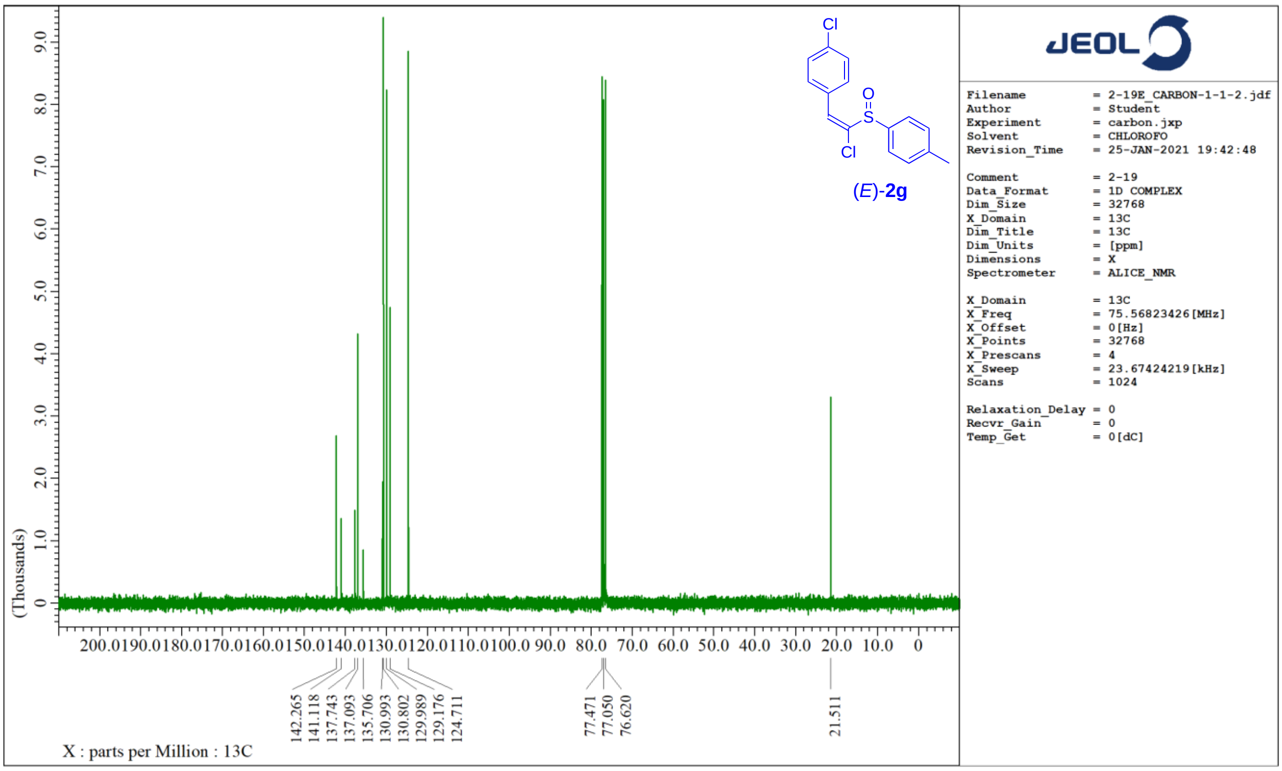

^{1}H NMR of (*Z*)-**2g**

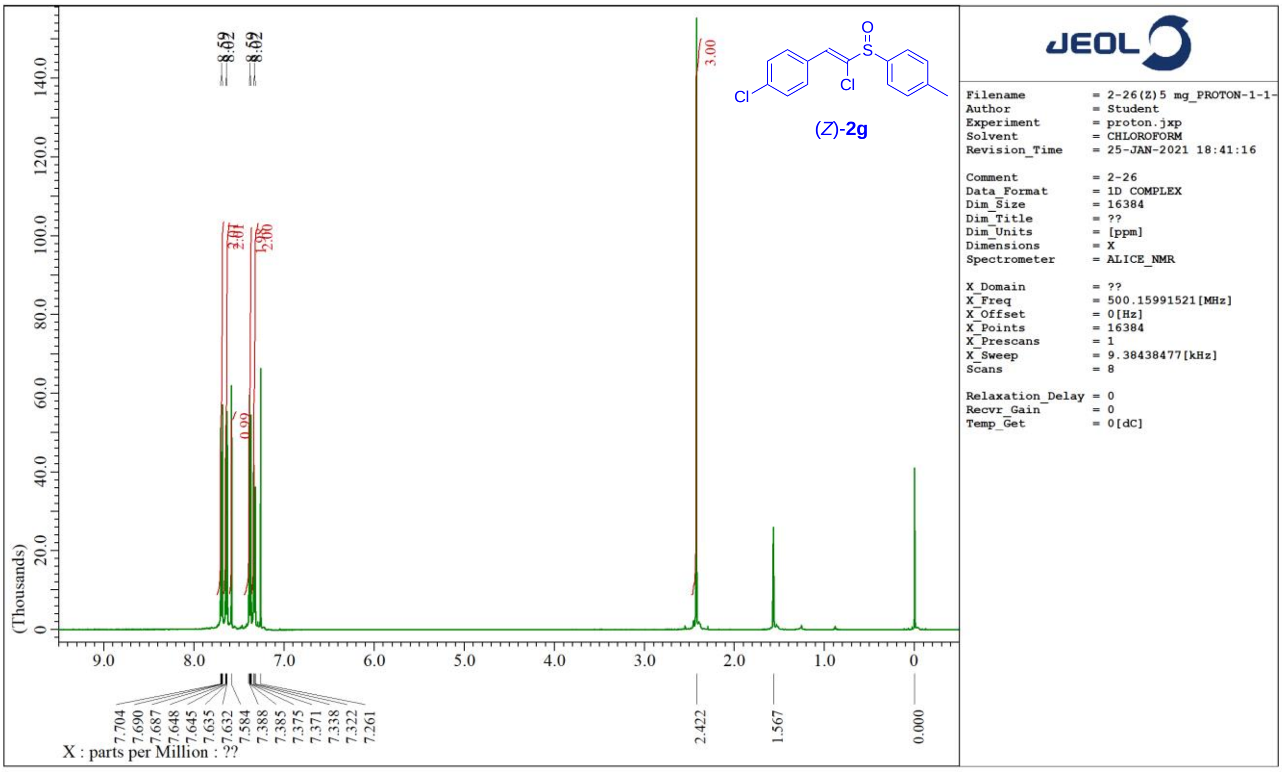

^{13}C NMR of (*Z*)-**2g**

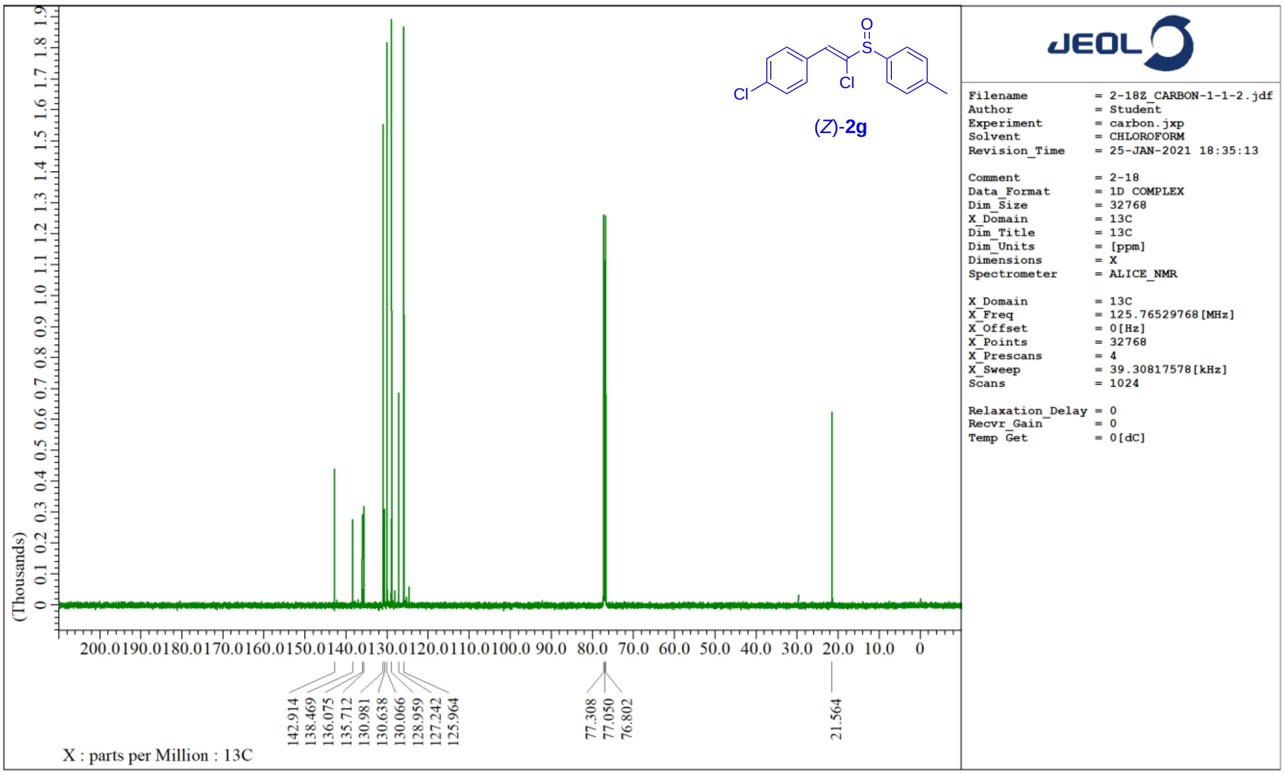

^{1}H NMR of **4a**

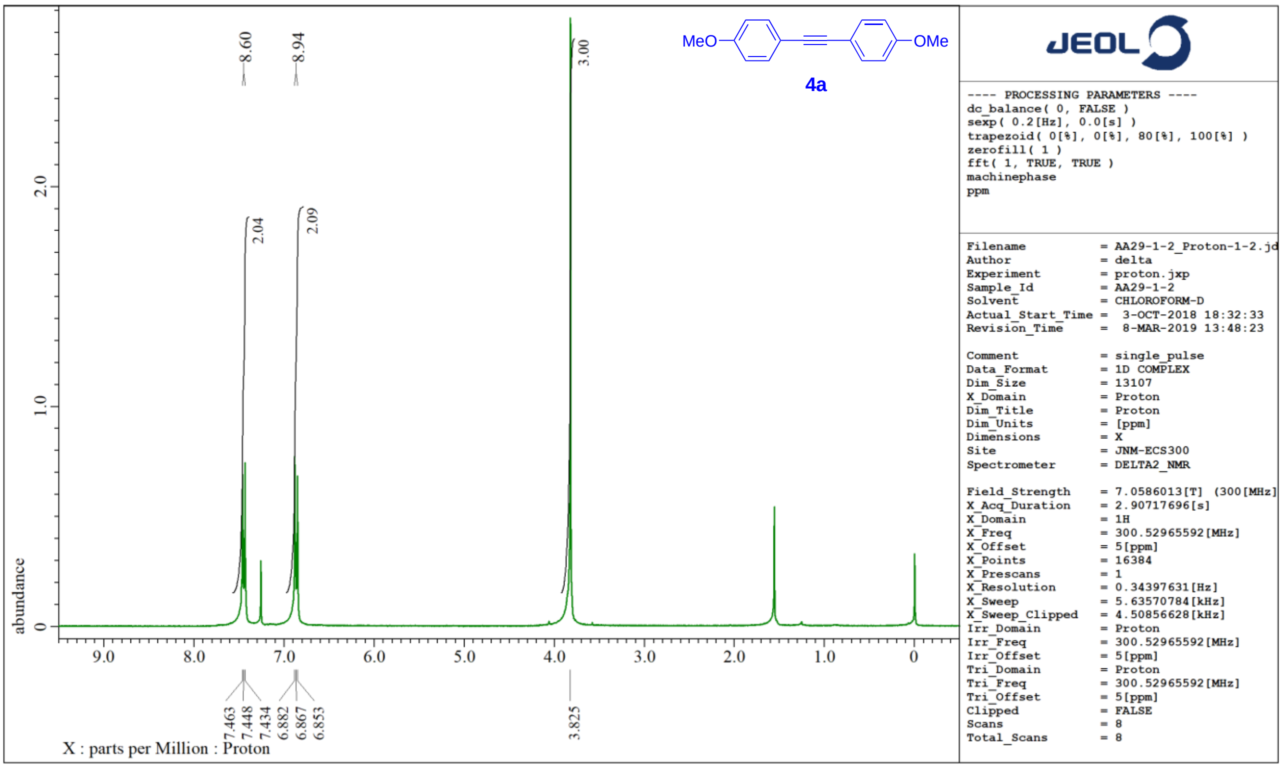

^{13}C NMR of **4a**

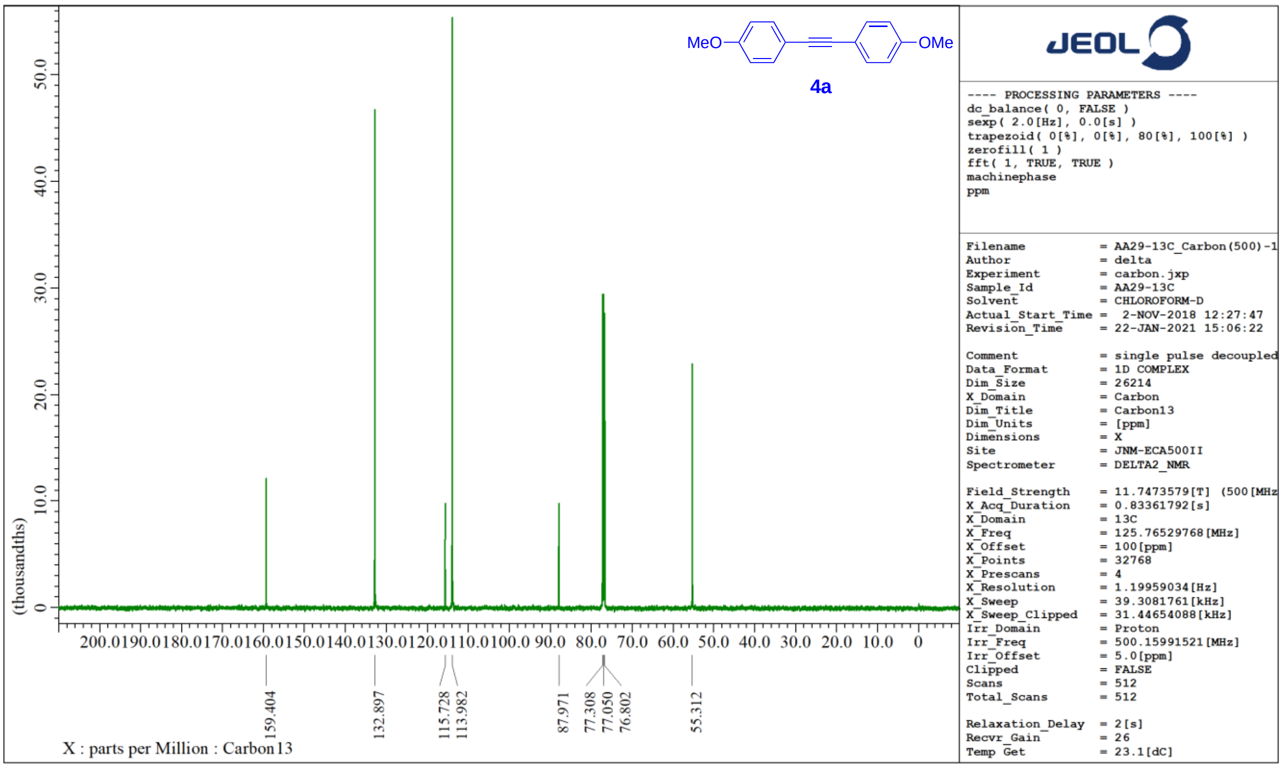

^{1}H NMR of **9d**

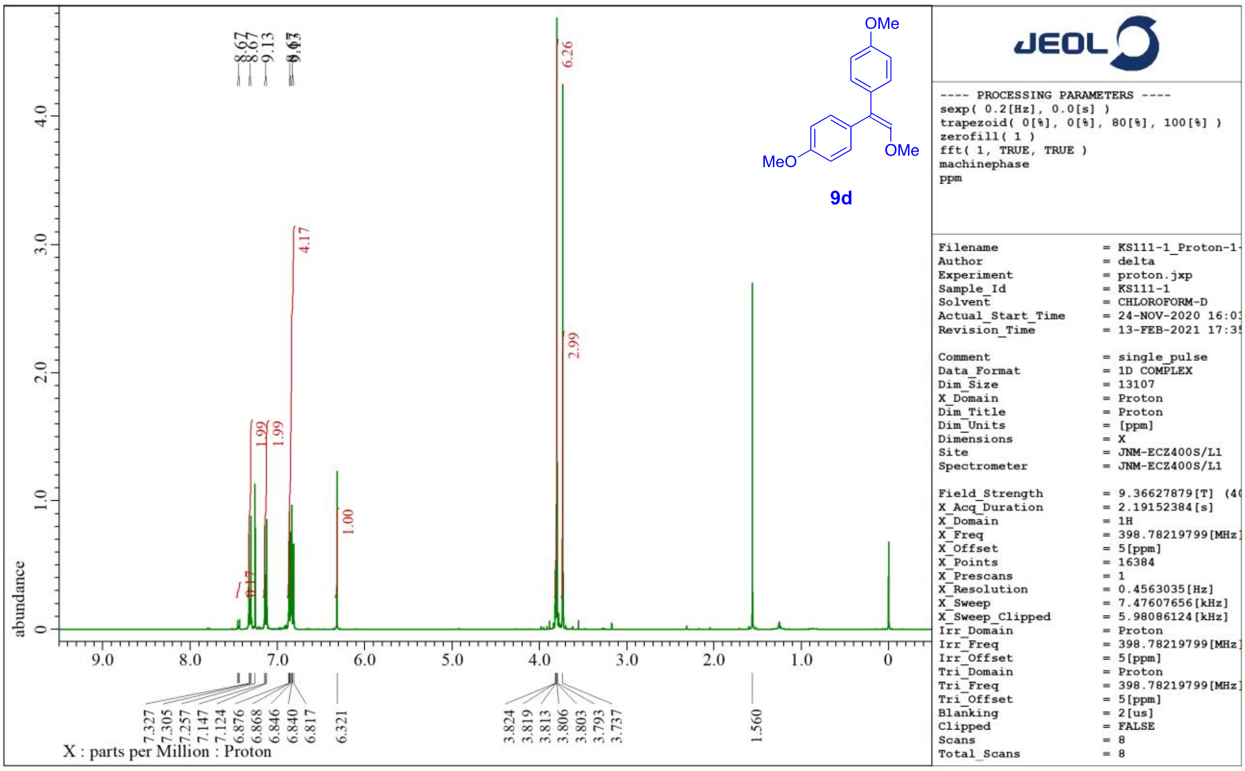

^{1}H NMR of **9e**

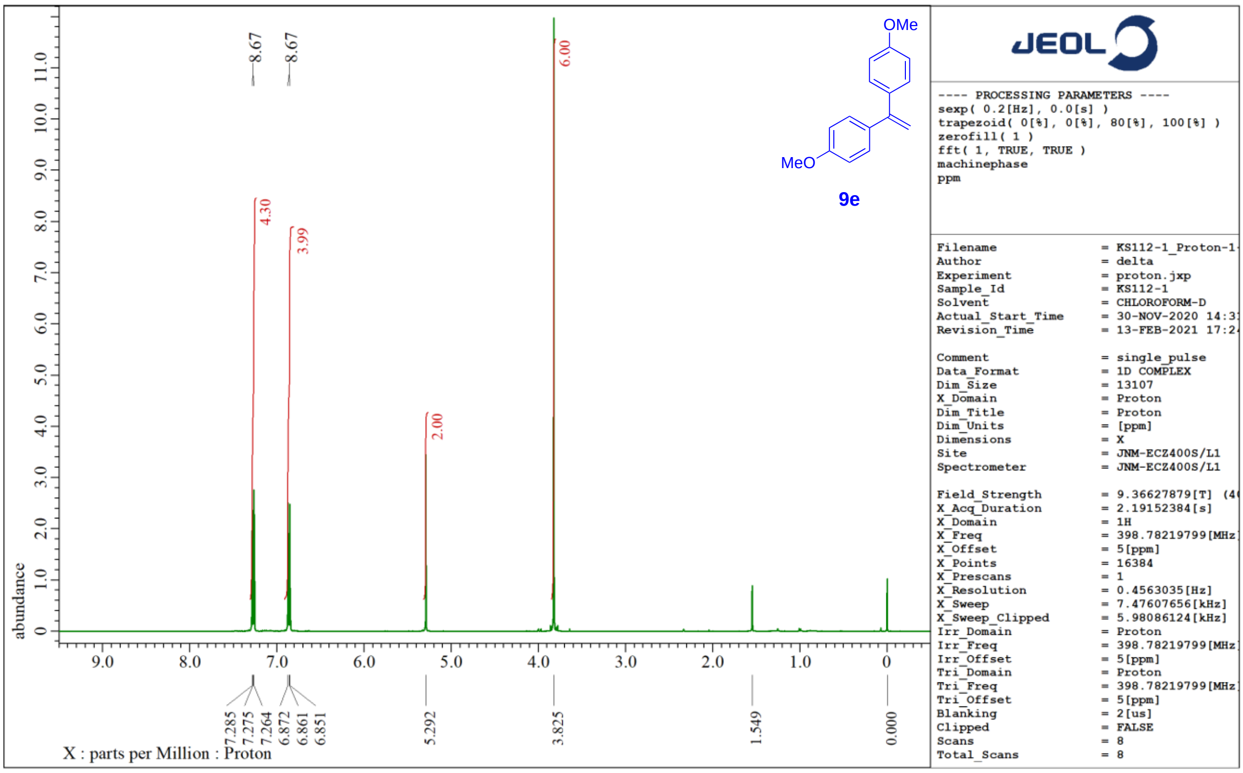

^{1}H NMR of **10**

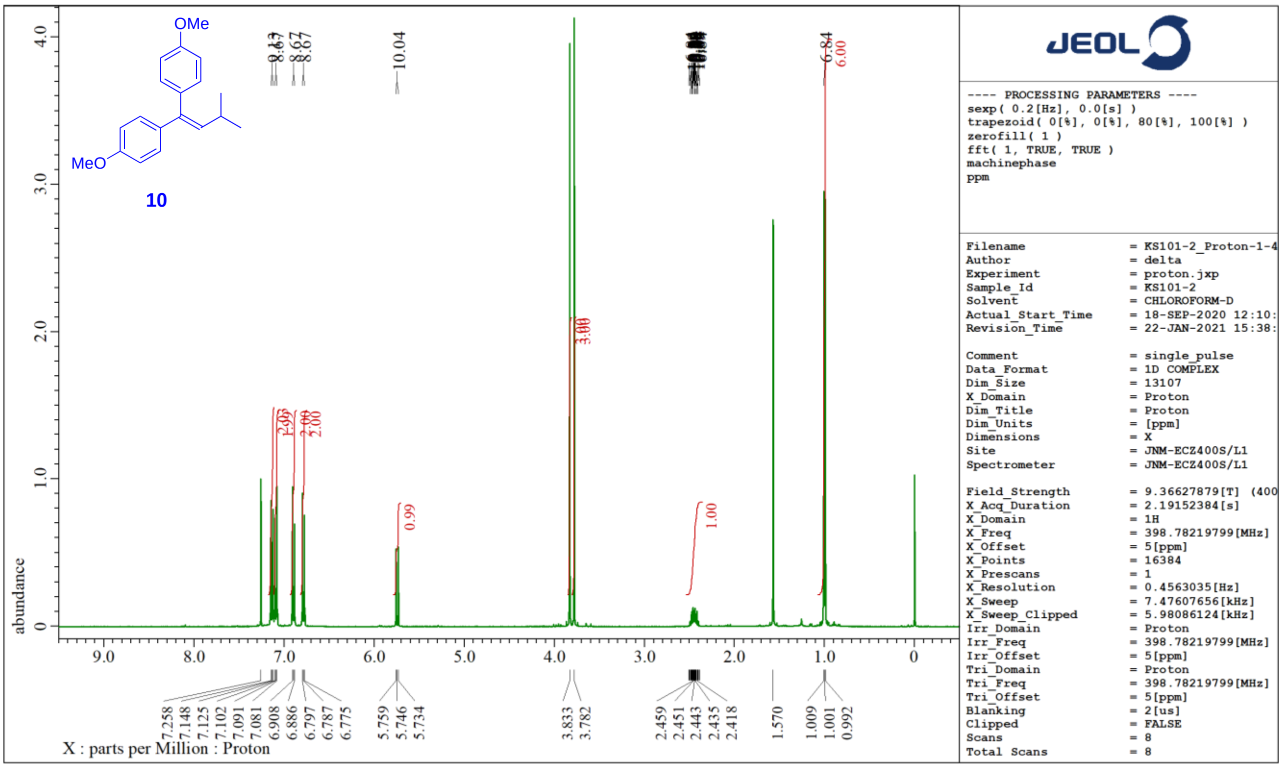

^{13}C NMR of **10**

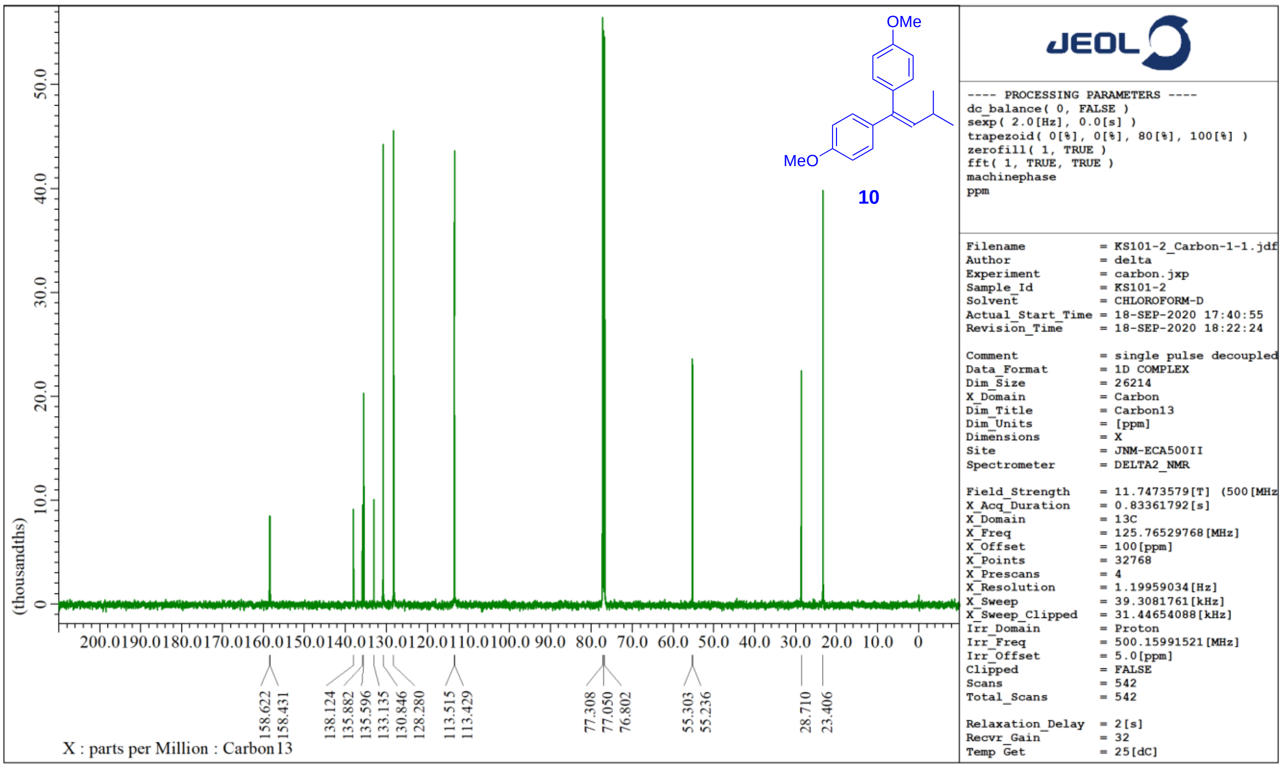

^{1}H NMR of **11**

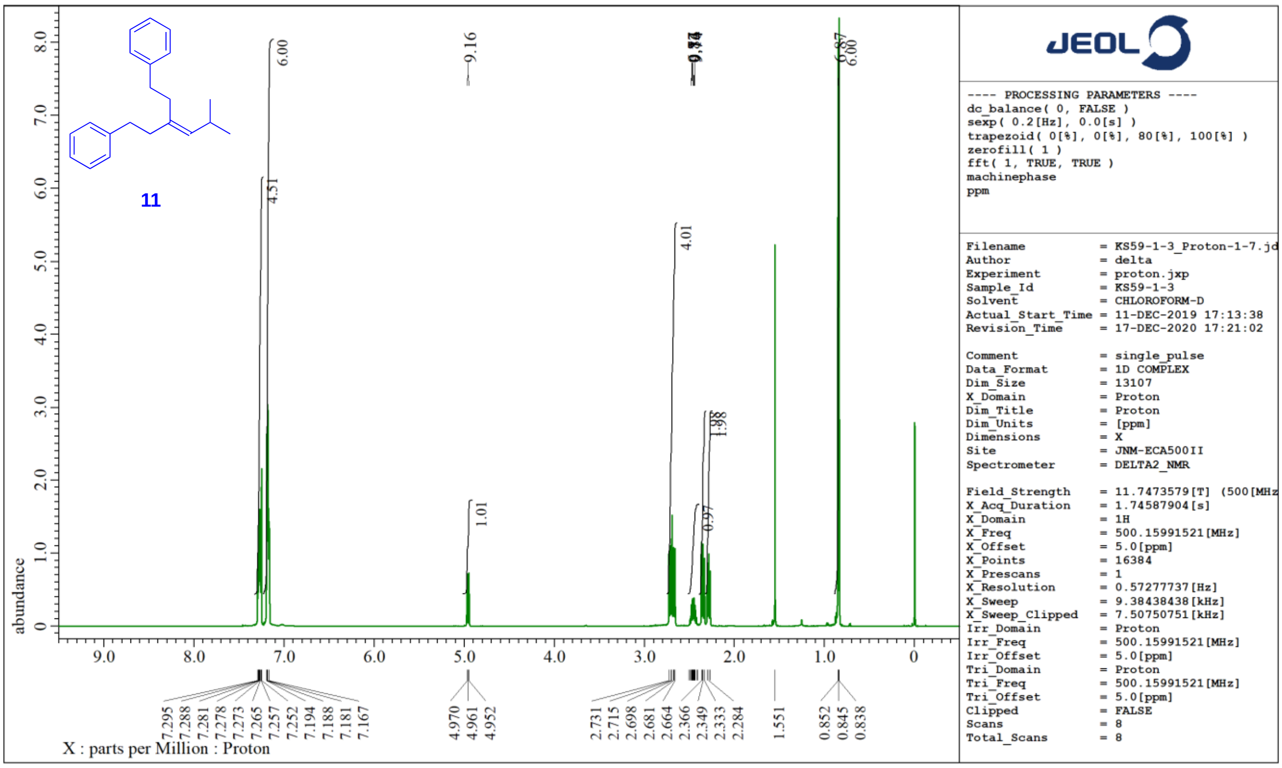

^{13}C NMR of **11**

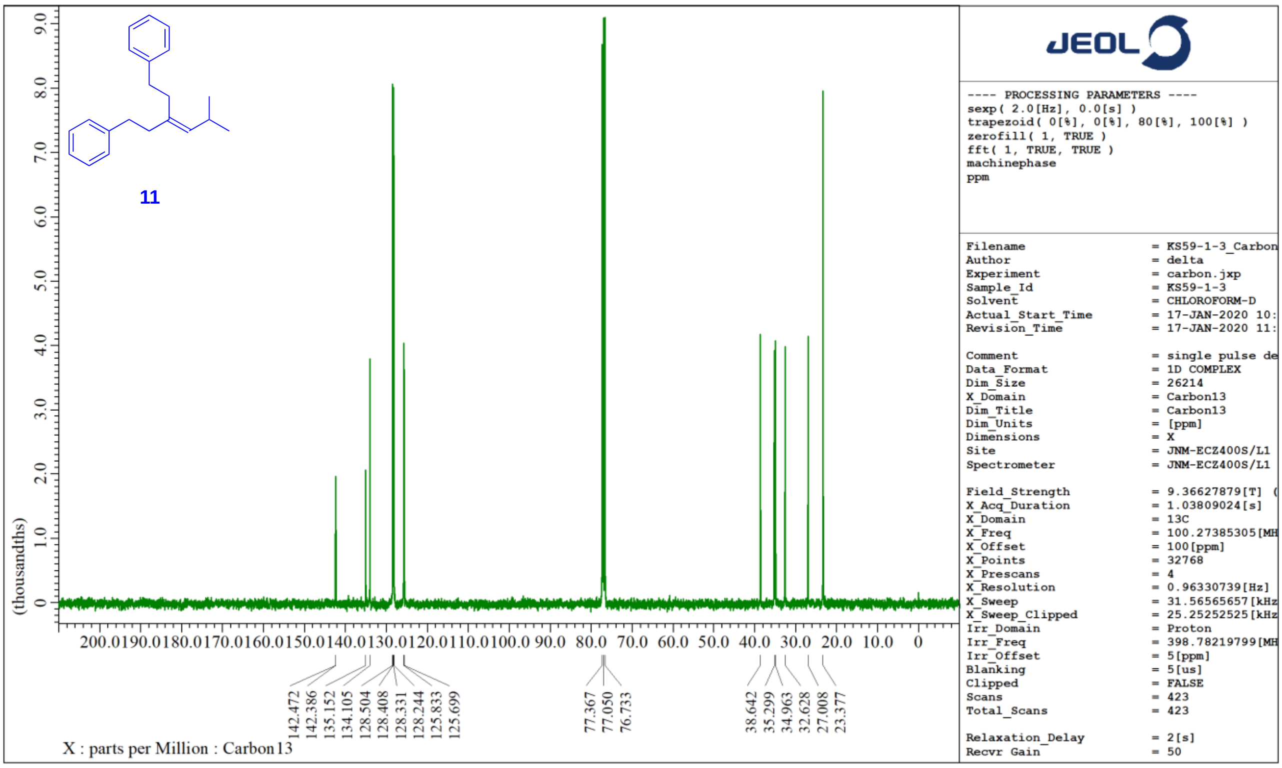

^{1}H NMR of **4b**

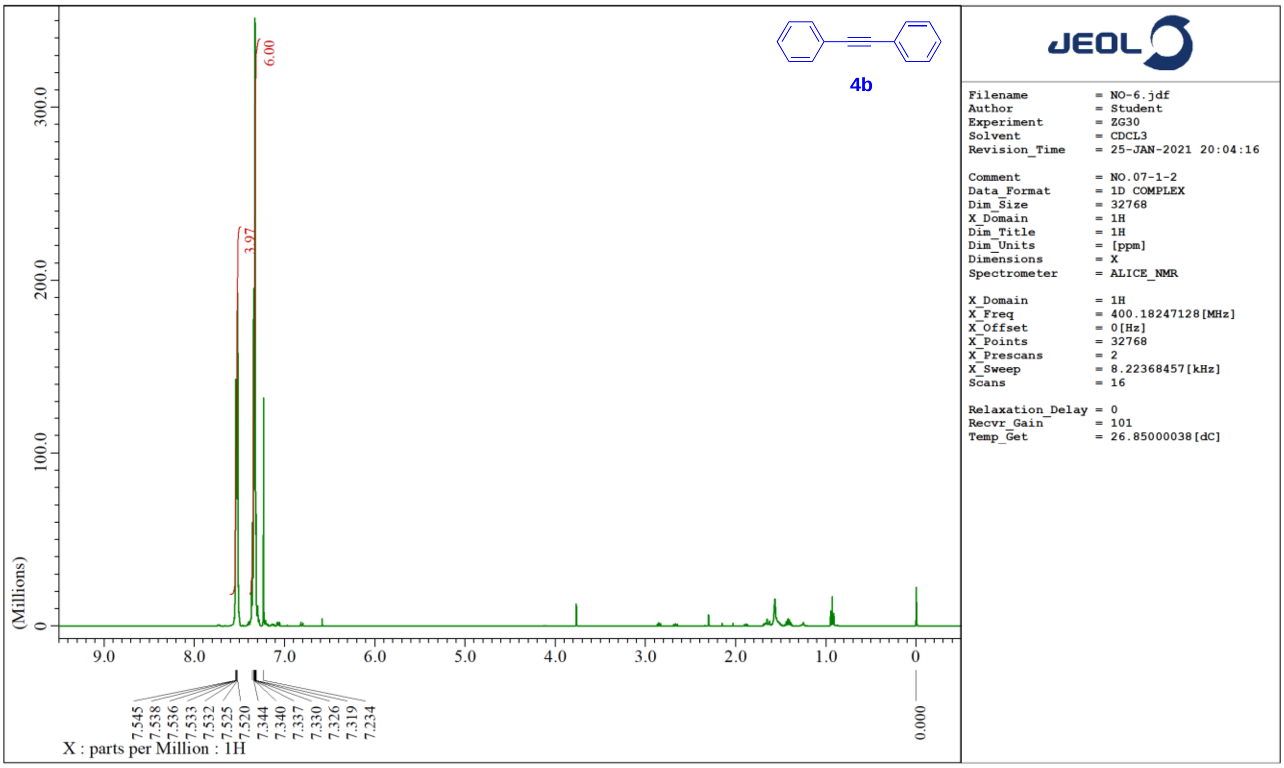

^{13}C NMR of **4b**

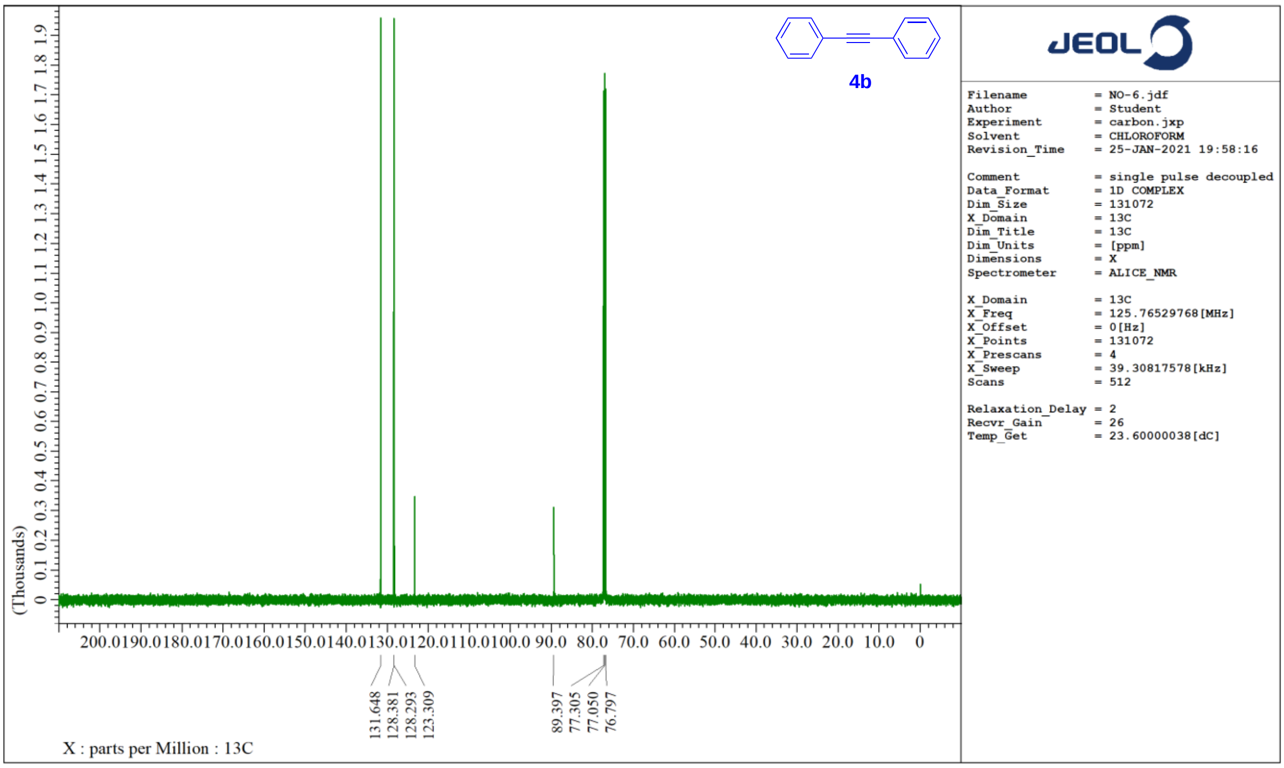

^{1}H NMR of **4d**

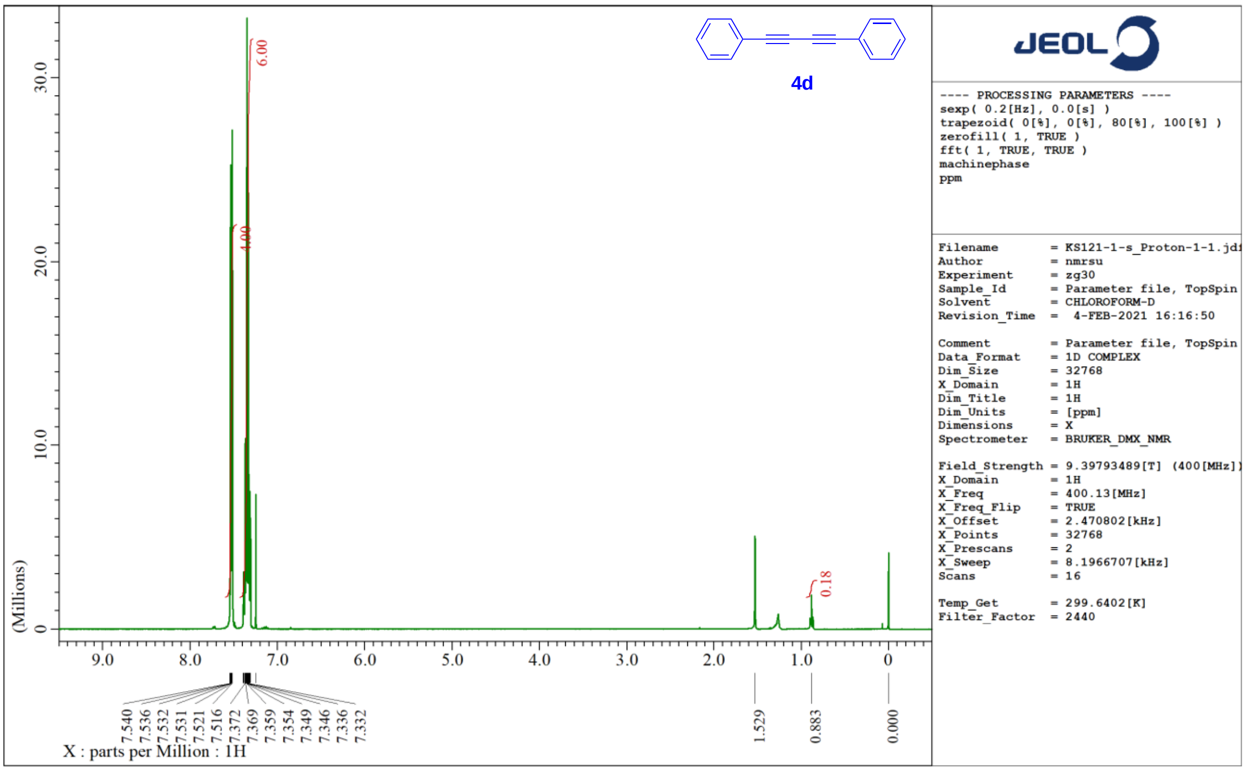

^{13}C NMR of **4d**

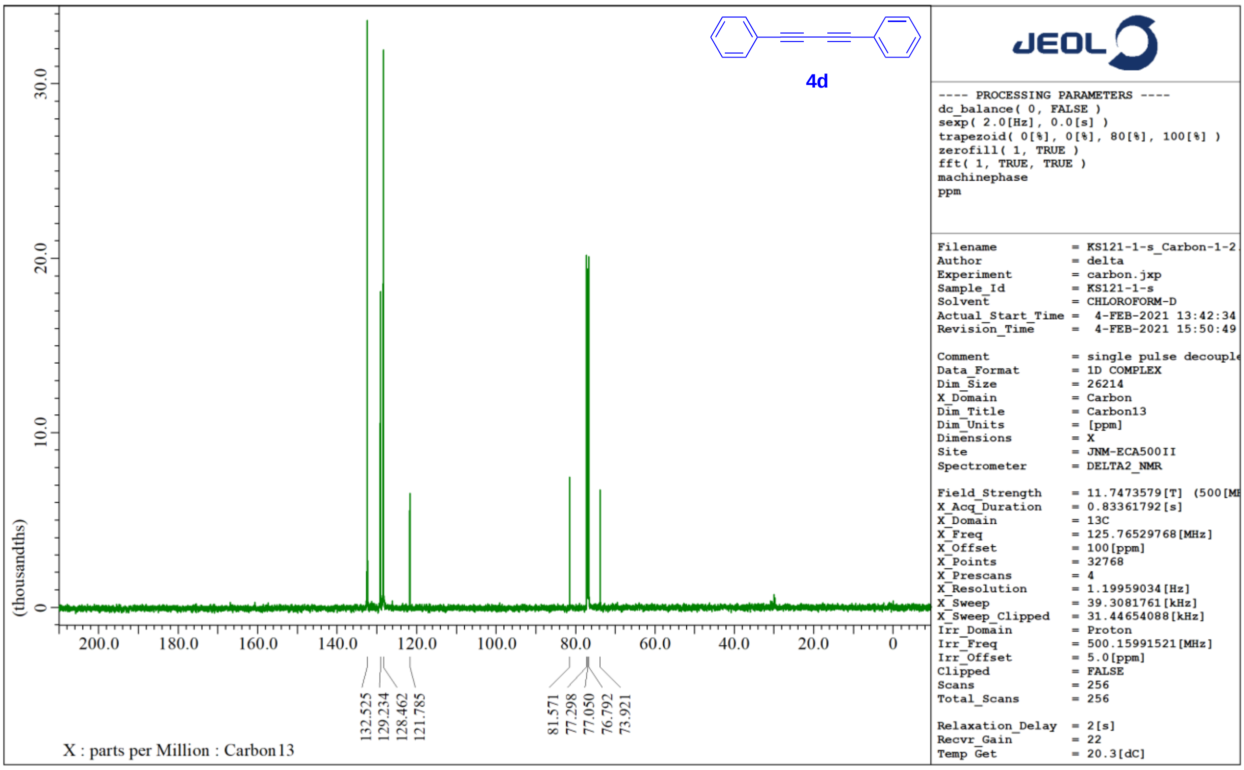

^{1}H NMR of **4e**

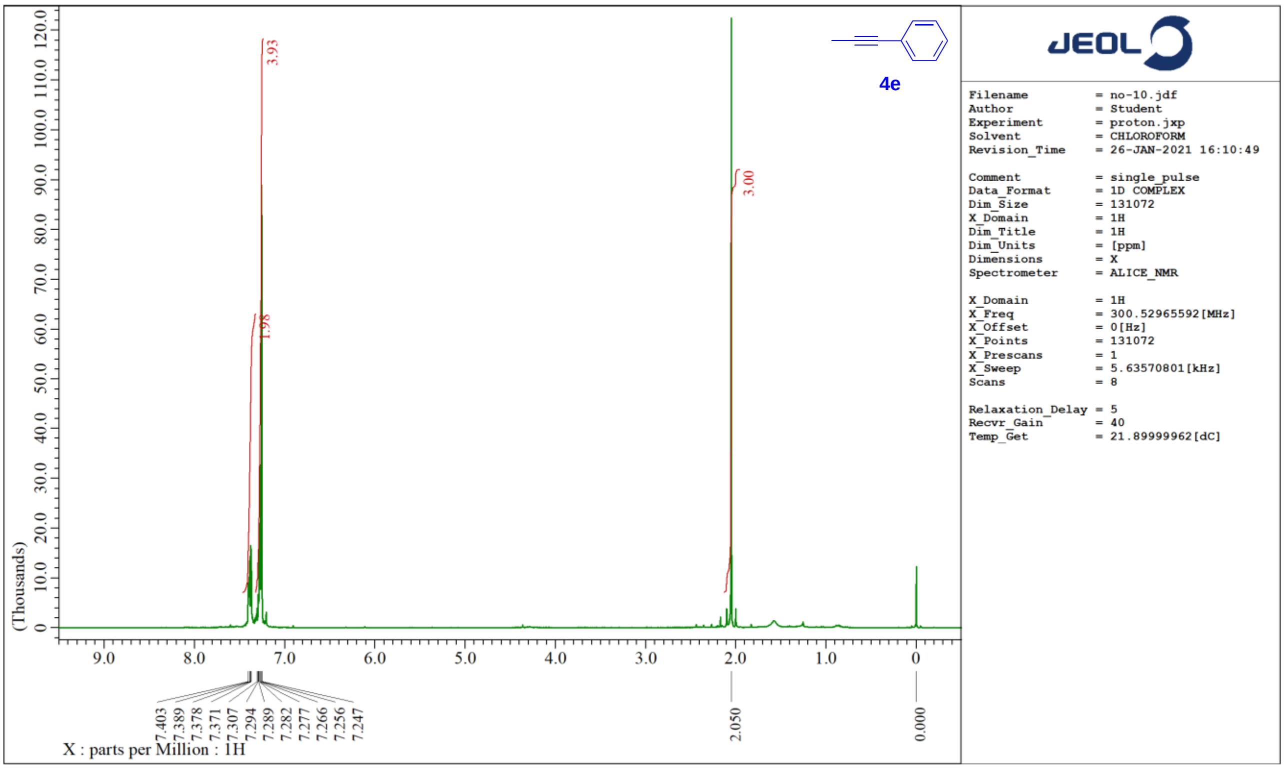

^{13}C NMR of **4e**

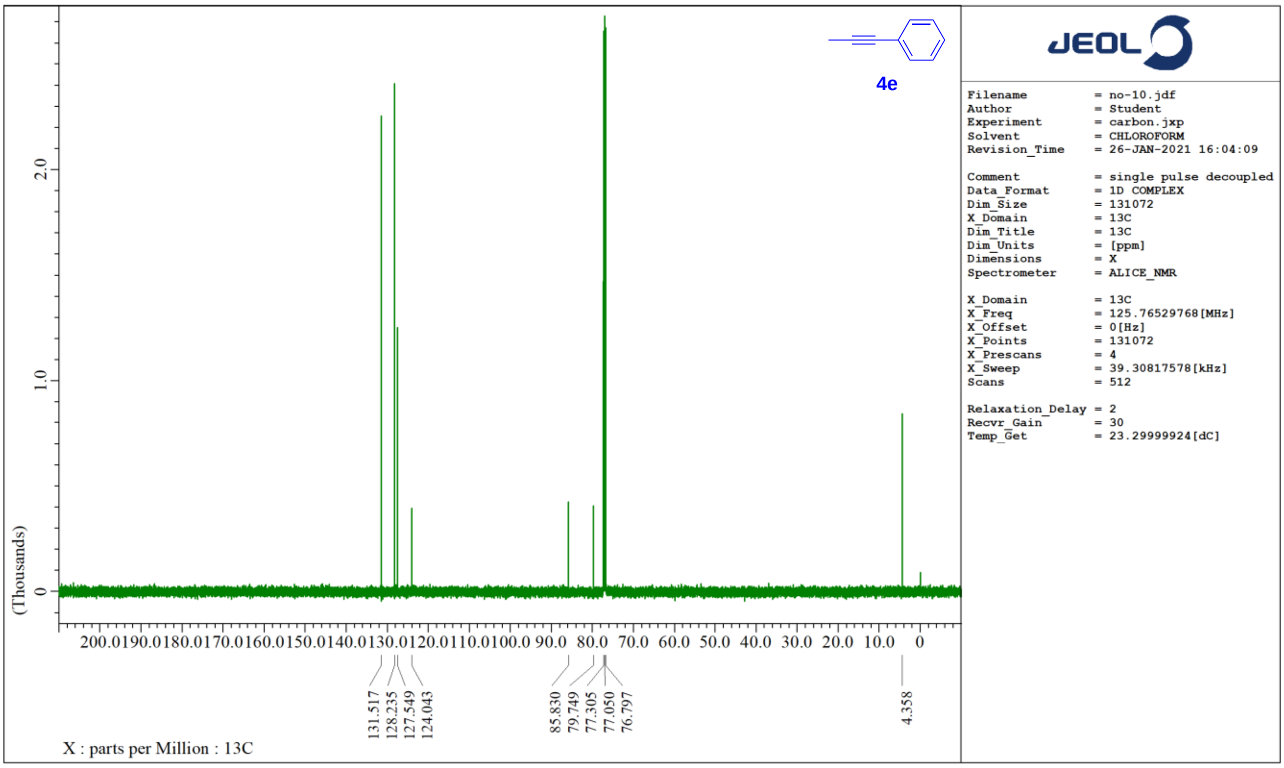

^{1}H NMR of [2-^{13}C]-**4e**

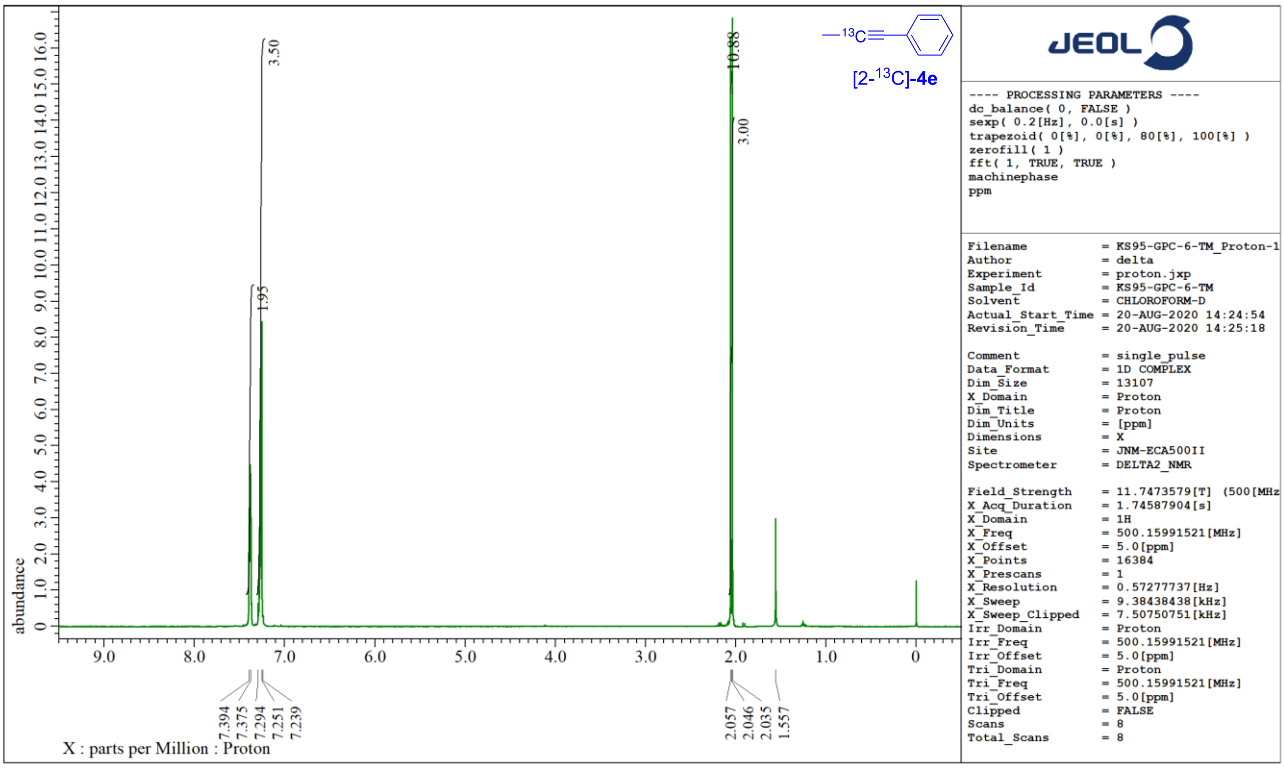

^{13}C NMR of [2-^{13}C]-**4e**

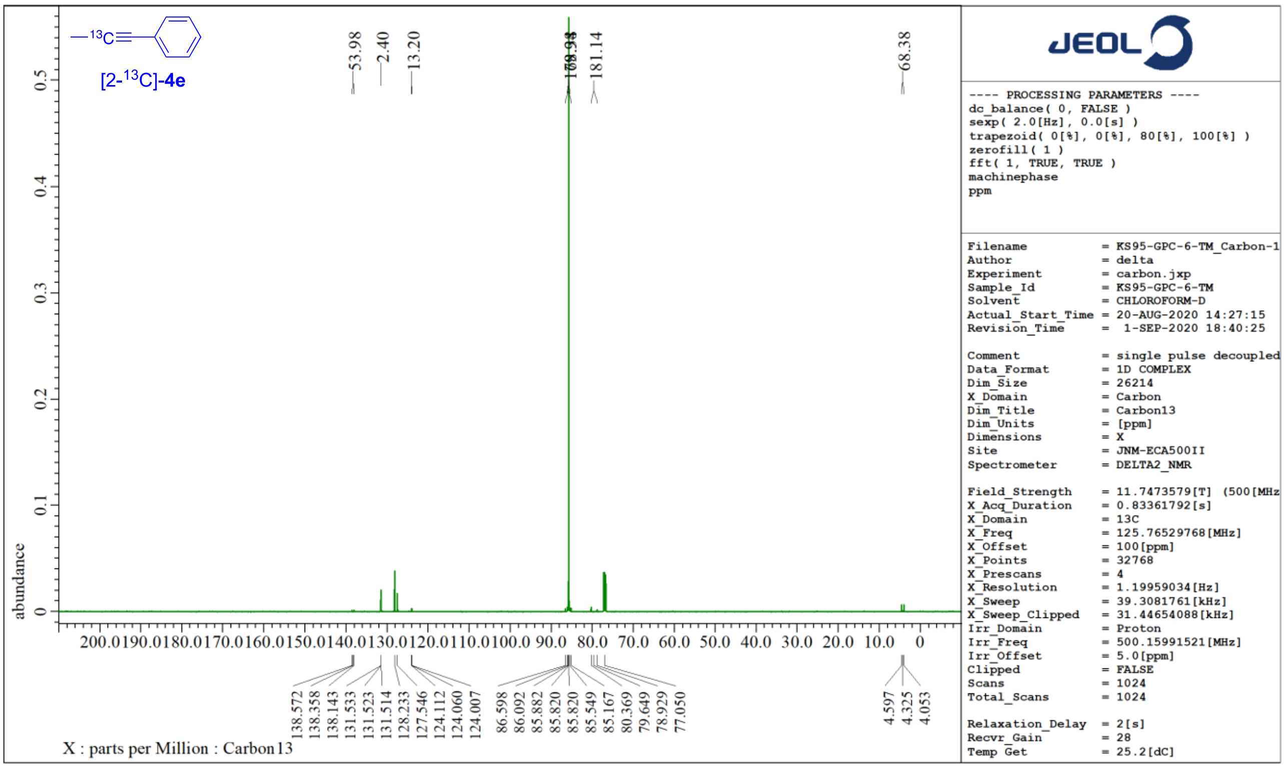

^{1}H NMR of [2-^{13}C]-propylbenzene

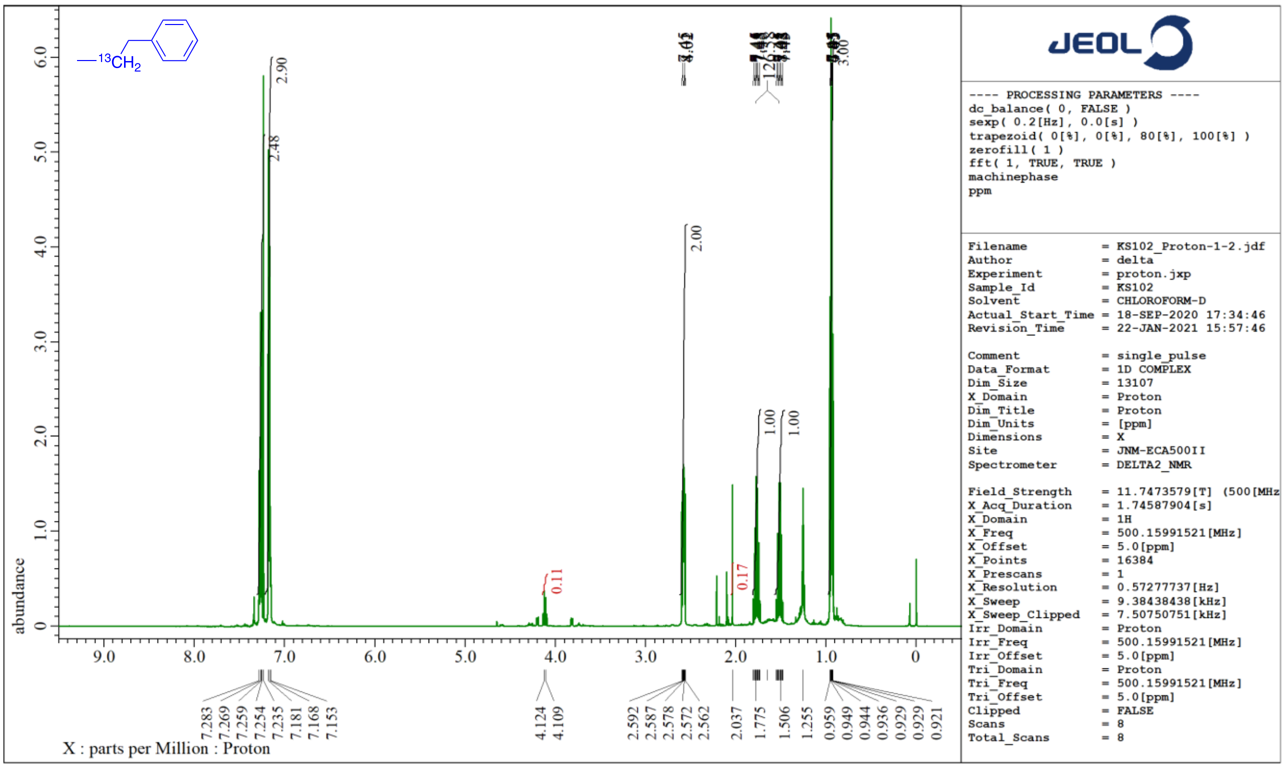

^{13}C NMR of [2-^{13}C]-propylbenzene

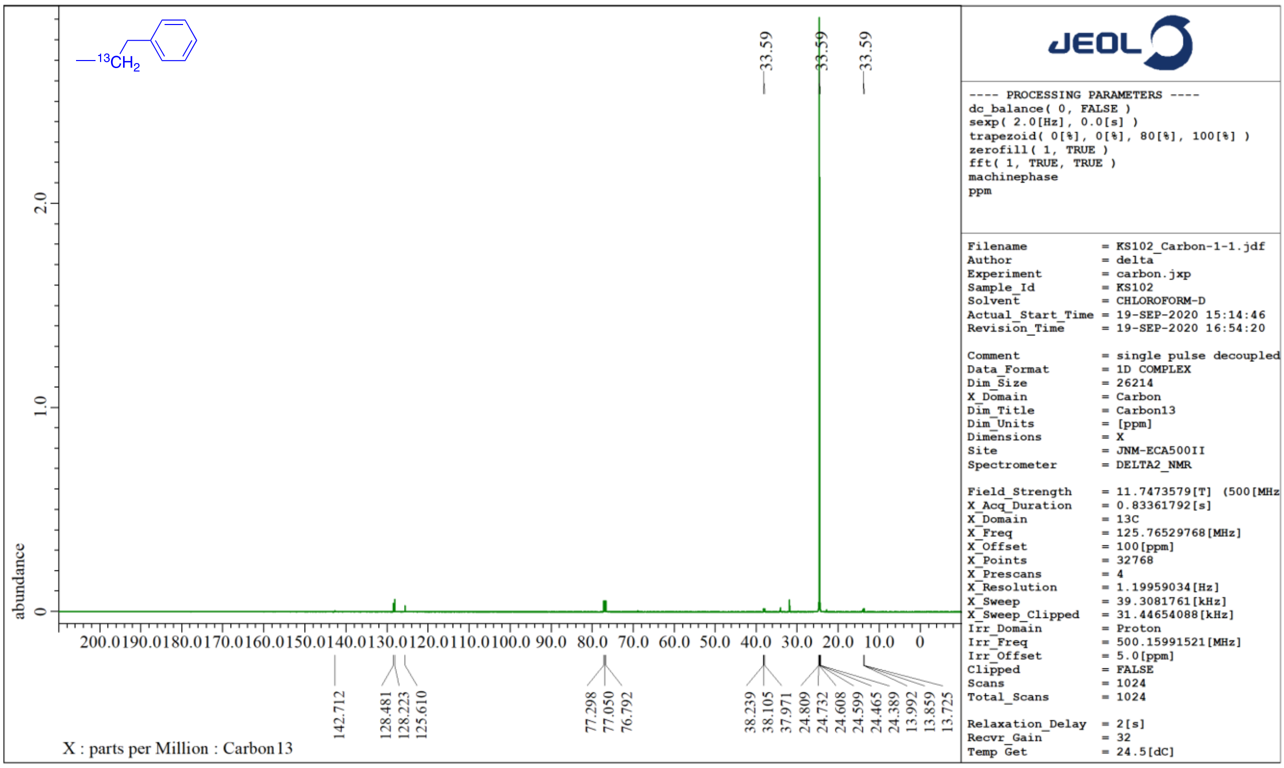

^{1}H NMR of **4f**

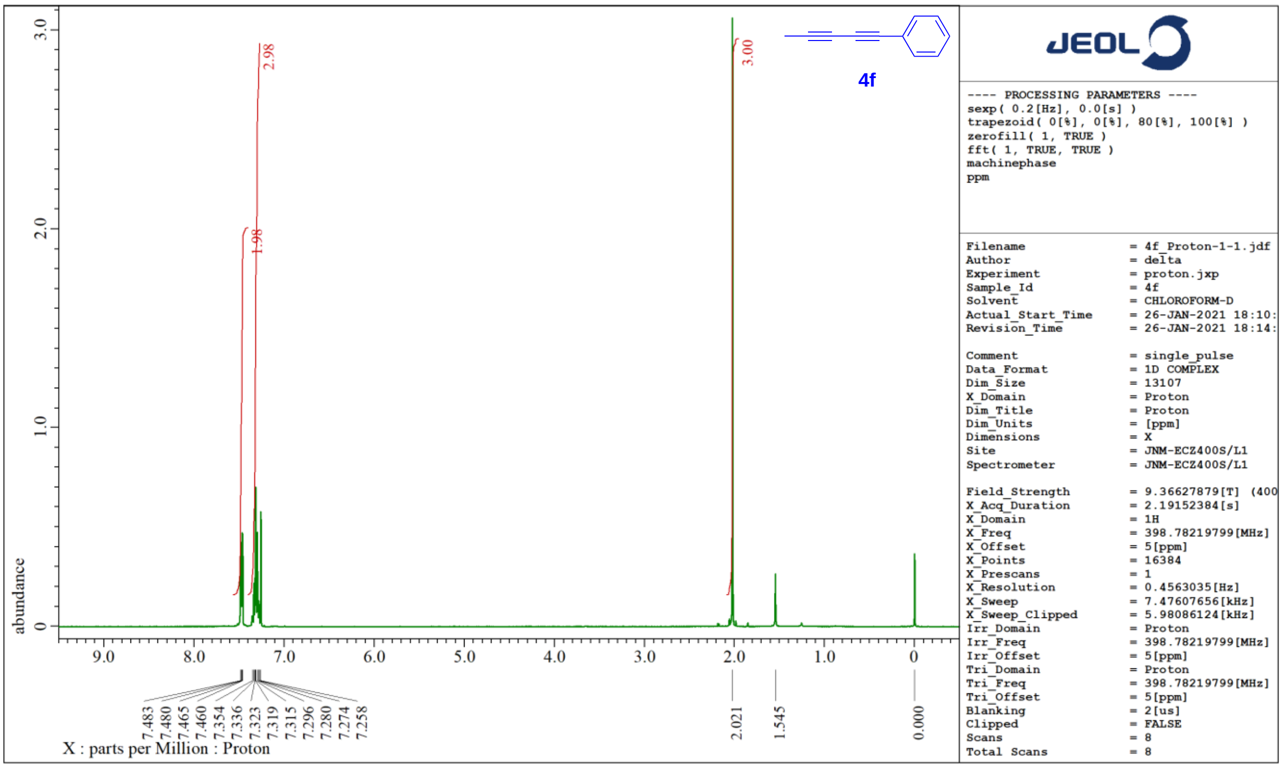

^{13}C NMR of **4f**

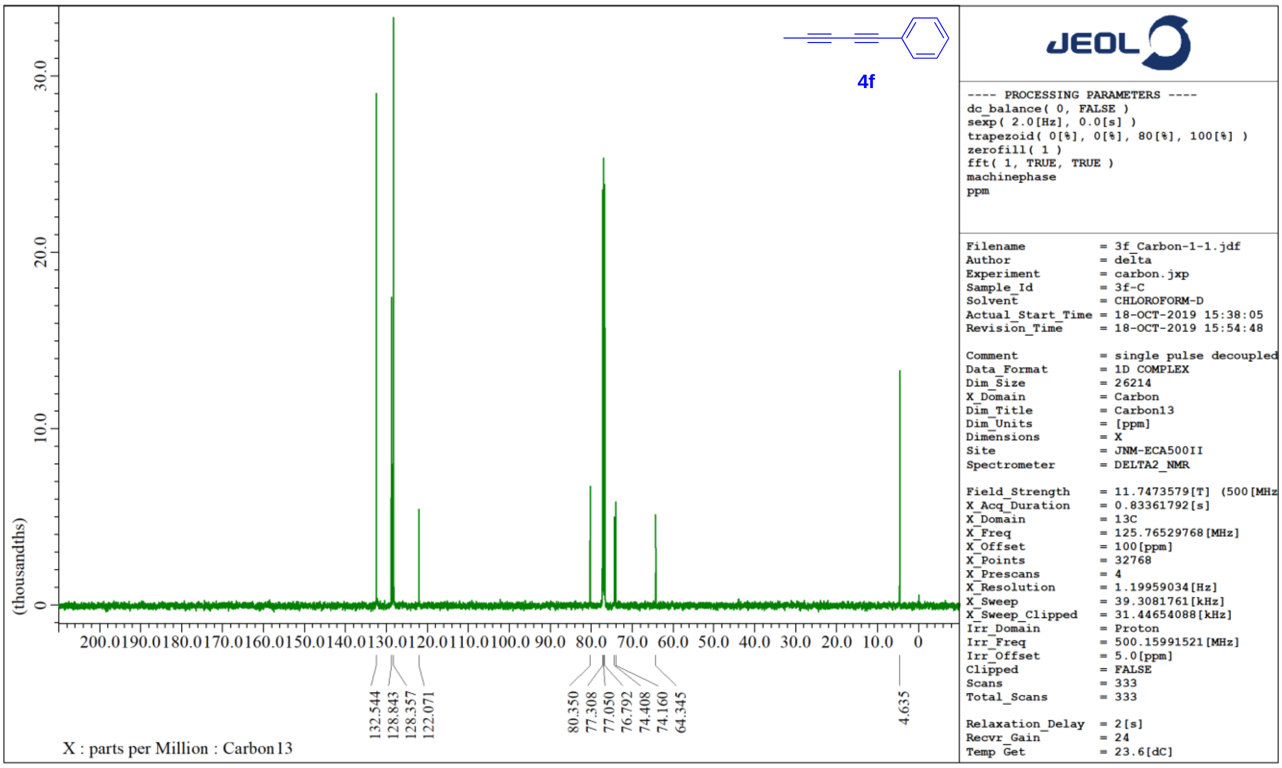

^{1}H NMR of **4g**

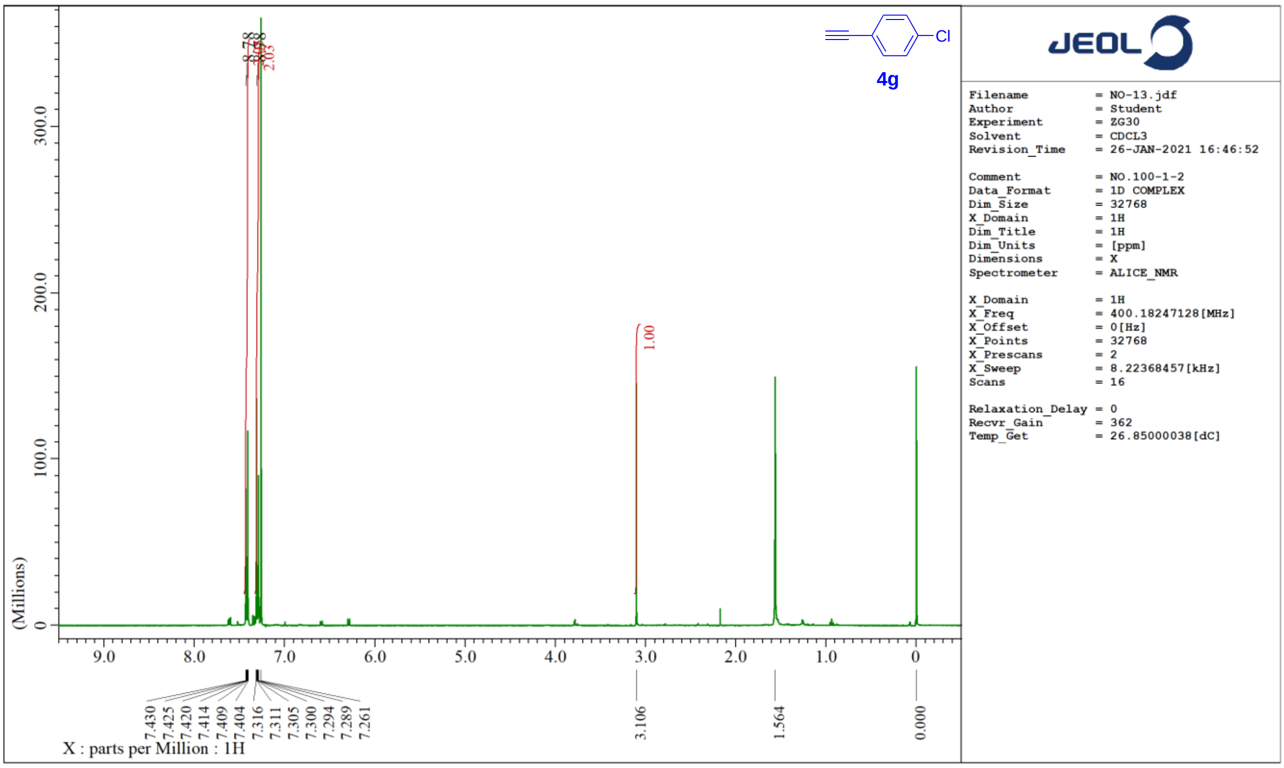

^{13}C NMR of **4g**

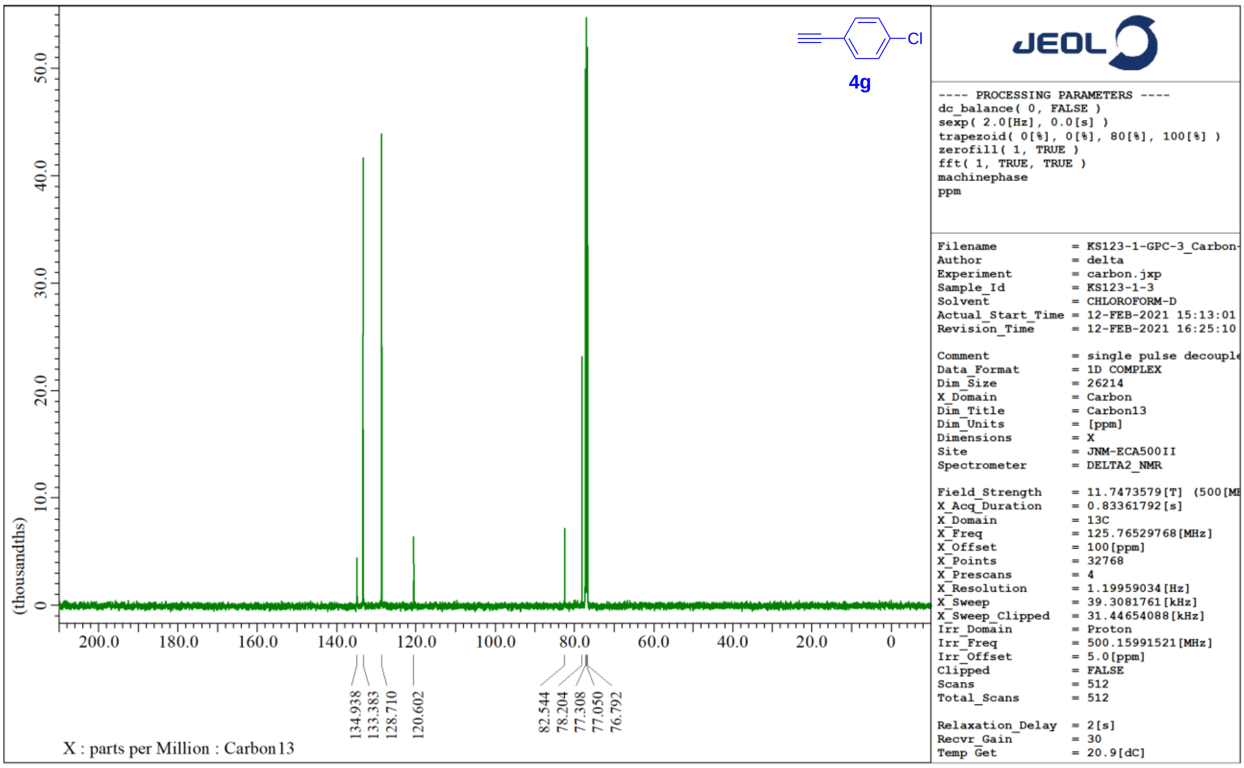

Computational methods

DFT calculations were performed using the Gaussian 09 software [11]. The geometries were fully optimized in the gas phase without symmetry constraints at the B3LYP/6-311++G(d,p) level of theory [12]. The QST3 method was used to locate transition states [13]. The frequency calculations were performed at the same level to confirm that the structures correspond to energy minima or transition states (zero or one imaginary frequencies, respectively). Starting from the transition-state structures, the reaction paths were followed using an intrinsic reaction coordinate (IRC) analysis [14]. The Jmol program was used to visualize the molecular structures [15].

(*E*)-PhMeC=CClMgCl•2(OMe$_2$) [(*E*)-3e]

No. of imaginary frequencies: 0

E_0: -1778.700728 hartree

$E_0 + E_{ZPE}$: -1778.395893 hartree

$E_0 + E_{tot}$: -1778.371287 hartree

$E_0 + H_{corr}$: -1778.370343 hartree

$E_0 + G_{corr}$: -1778.454738 hartree

Mg	0.987611	-0.396165	-0.143062
C	-0.212418	1.375120	-0.194129
C	-1.534895	1.627638	-0.179265
Cl	0.874153	2.881747	-0.045386
O	1.455197	-0.689261	1.890504
O	2.916193	0.143442	-0.827449
Cl	0.958214	-2.551221	-0.940269
C	-2.452730	0.449445	-0.252205
C	-3.545976	0.345330	0.625483
C	-2.263206	-0.578429	-1.186720
C	-4.396082	-0.755899	0.585885
H	-3.725510	1.127726	1.354302
C	-3.110051	-1.684963	-1.223338
H	-1.456041	-0.501389	-1.904883
C	-4.179669	-1.779891	-0.337033
H	-5.228470	-0.816402	1.279007
H	-2.930636	-2.469270	-1.949795
H	-4.842289	-2.637545	-0.367935
C	3.053473	0.878634	-2.055520
H	2.208453	1.560402	-2.119643
H	3.981958	1.456002	-2.033090
H	3.060522	0.189707	-2.905834
C	4.015545	-0.750026	-0.596458
H	3.854109	-1.220856	0.371214
H	4.049309	-1.520118	-1.371297
H	4.949149	-0.179793	-0.580090
C	1.409418	-1.967970	2.549764
H	0.522353	-2.023653	3.187652
H	1.357711	-2.726470	1.771412
H	2.310505	-2.100279	3.156577
C	1.506877	0.414378	2.811515
H	1.518437	1.332483	2.227392
H	0.624323	0.401841	3.457538
H	2.413602	0.342127	3.419813
C	-2.204962	2.978331	-0.048366
H	-3.108418	3.019968	-0.662207
H	-2.507772	3.173255	0.987255
H	-1.535948	3.783682	-0.346614

(*Z*)-PhMeC=CClMgCl•2(OMe$_2$) [(*Z*)-3e]

No. of imaginary frequencies: 0

E_0: -1778.699864 hartree

$E_0 + E_{ZPE}$: -1778.395040 hartree

$E_0 + E_{tot}$: -1778.370499 hartree

$E_0 + H_{corr}$: -1778.369554 hartree

$E_0 + G_{corr}$: -1778.454657 hartree

Mg	-1.687026	-0.238196	-0.308271
C	0.417804	-0.007737	-0.040541
C	1.480852	-0.508109	-0.689993
Cl	0.827268	1.090301	1.411375
O	-2.624184	1.652167	-0.469540
O	-2.507911	-0.902329	1.517747
Cl	-2.903678	-1.456067	-1.832157
C	2.920682	-0.248689	-0.381522
C	3.730985	-1.267870	0.134310
C	3.502617	0.997418	-0.643370
C	5.081236	-1.044010	0.396260
H	3.297388	-2.240011	0.344076
C	4.854524	1.220359	-0.391303
H	2.887246	1.795047	-1.043412
C	5.648948	0.201060	0.131290
H	5.689880	-1.843160	0.805507
H	5.287443	2.192364	-0.601946
H	6.700519	0.375359	0.330433
C	-1.745881	-1.154587	2.711912
H	-0.754842	-0.732314	2.564625
H	-2.231112	-0.672612	3.566082
H	-1.677676	-2.232778	2.884534
C	-3.854566	-1.397512	1.606839

H	-4.320517	-1.254543	0.634457
H	-3.841903	-2.465393	1.842856
H	-4.397395	-0.850754	2.384073
C	-3.712486	1.932348	-1.369544
H	-3.393284	2.669029	-2.112700
H	-3.974396	0.997280	-1.859259
H	-4.564675	2.322890	-0.804596
C	-2.166292	2.828773	0.219449
H	-1.320403	2.542014	0.840695
H	-1.846511	3.581177	-0.507502
H	-2.974637	3.232649	0.837063
C	1.246614	-1.435491	-1.868218
H	1.698558	-1.021238	-2.776285
H	1.721129	-2.407820	-1.695893
H	0.184999	-1.605231	-2.059327

[(*E*)-PhMeC=CClMgCl•2(OMe$_2$)]‡ [(*E*)-3e‡]
No. of imaginary frequencies: 1 (243.2*i* cm^{-1})
E_0: -1778.673338 hartree
$E_0 + E_{ZPE}$: -1778.371790 hartree
$E_0 + E_{tot}$: -1778.346635 hartree
$E_0 + H_{corr}$: -1778.345691 hartree
$E_0 + G_{corr}$: -1778.430985 hartree

Mg	1.242719	0.234796	-0.182190
C	-0.925405	1.022805	0.102293
C	-1.773079	1.913911	-0.165516
Cl	1.972811	2.423855	0.234005
O	1.230729	-0.854597	1.636843
O	3.358677	-0.452793	-0.367278
Cl	0.603365	-1.275566	-1.864311
C	-2.472955	0.349264	-0.052184
C	-3.257811	0.125465	1.093465
C	-2.766248	-0.338900	-1.242126
C	-4.320566	-0.768301	1.047384
H	-3.022379	0.659800	2.006754
C	-3.825454	-1.238731	-1.277328
H	-2.132103	-0.189779	-2.106644
C	-4.603766	-1.450919	-0.138331
H	-4.926784	-0.936211	1.930279
H	-4.039991	-1.780048	-2.191367
H	-5.430683	-2.151479	-0.172320
C	4.288921	0.300780	-1.157748
H	4.032869	1.350190	-1.033636
H	5.307861	0.118374	-0.799631
H	4.210957	0.009411	-2.210883
C	3.639494	-1.854591	-0.410357

H	2.925394	-2.350989	0.242537
H	3.521341	-2.239386	-1.427021
H	4.658791	-2.036274	-0.051259
C	0.294764	-1.912906	1.869310
H	-0.565882	-1.541391	2.434466
H	-0.028477	-2.272788	0.893566
H	0.780021	-2.721606	2.426006
C	1.770195	-0.285934	2.836952
H	2.418185	0.536524	2.539708
H	0.962144	0.096574	3.468842
H	2.340674	-1.044302	3.382980
C	-2.470742	3.161142	-0.485982
H	-2.945225	3.111938	-1.469037
H	-3.231320	3.405060	0.259570
H	-1.722350	3.958524	-0.496937

MeC≡CPh•MgCl$_2$•2(OMe$_2$) (4e•MgCl$_2$)
No. of imaginary frequencies: 0
E_0: -1778.752561 hartree
$E_0 + E_{ZPE}$: -1778.448488 hartree
$E_0 + E_{tot}$: -1778.422622 hartree
$E_0 + H_{corr}$: -1778.421678 hartree
$E_0 + G_{corr}$: -1778.511354 hartree

Mg	1.911076	-0.013126	-0.359925
C	-2.538710	1.598971	-0.372733
C	-1.907650	2.616281	-0.534134
Cl	2.446169	2.208557	-0.382877
O	0.764243	-0.231106	1.369374
O	3.728186	-0.909960	0.186089
Cl	1.027031	-1.538741	-1.808075
C	-3.278636	0.388791	-0.200573
C	-4.417578	0.352792	0.624645
C	-2.881247	-0.790358	-0.857991
C	-5.135767	-0.827680	0.786498
H	-4.732736	1.258656	1.129122
C	-3.606190	-1.966419	-0.690622
H	-2.001307	-0.777888	-1.490518
C	-4.734374	-1.991329	0.129643
H	-6.013566	-0.838285	1.423238
H	-3.287493	-2.865407	-1.206398
H	-5.298218	-2.909031	0.254100
C	4.784309	-0.197168	0.853480
H	4.448141	0.828887	0.986119
H	4.993137	-0.665338	1.820375
H	5.684943	-0.208635	0.233068
C	4.085668	-2.265897	-0.136222

H	3.249446	-2.697533	-0.681813
H	4.979908	-2.269722	-0.766010
H	4.277699	-2.826137	0.784119
C	-0.007827	-1.415709	1.644641
H	-1.073999	-1.184818	1.586223
H	0.241799	-2.150064	0.881876
H	0.244257	-1.791454	2.641019
C	0.451187	0.853806	2.264611
H	1.079867	1.694929	1.981300
H	-0.601672	1.126012	2.156195
H	0.660427	0.547951	3.294168
C	-1.140102	3.834658	-0.753980
H	-1.391885	4.281845	-1.720662
H	-1.347376	4.577272	0.022169
H	-0.066632	3.623499	-0.749686

Table S1. Selected geometric parameters of model compounds

compound	distance (Å)				angle (°)		
	C-Cl	C1-C2	C2-C3	C3-C1	C1-C2-C3	C2-C3-C1	C3-C1-C2
(*E*)-**3e**	1.864	1.346	1.495	2.425	117.0	29.6	33.3
(*E*)-**3e**‡	3.222	1.259	1.718	1.695	67.4	43.3	69.3
(*E*)-**4e**•$MgCl_2$	5.022	1.208	2.637	1.429	0.5	0.4	179.2

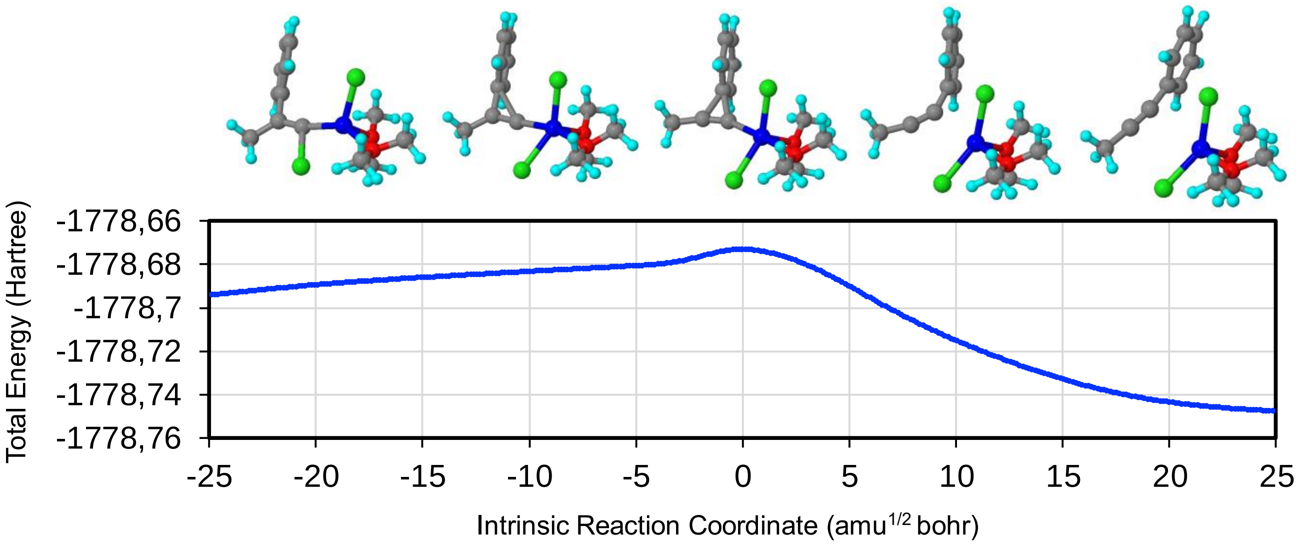

Figure S1. Intrinsic reaction coordinate for the FBW rearrangement of magnesium alkylidene carbenoid (*E*)-**3e**.

References

1. Satoh, T.; Takano, K.; Ota, H.; Someya, H.; Matsuda, K.; Koyama, M. *Tetrahedron* **1998**, *54*, 5557–5574.
2. Ishida, N.; Saitoh, H.; Sugiyama, S.; Satoh, T. *Tetrahedron* **2011**, *67*, 3081–3090.
3. Kimura, T.; Kobayashi, G.; Ijima, S.; Saito, S.; Imafuji, A.; Satoh, T. *Heteroatom Chem.* **2017**, *28*, https://doi.org/10.1002/hc.21395.
4. Novák, Z.; Nemes, P.; Kotschy, A. *Org. Lett.* **2004**, *6*, 4917–4920.
5. Lei, A.; Srivastava, M.; Zhang, X. *J. Org. Chem.* **2002**, *67*, 1969–1971.
6. Ma, S.; Xu, B.; Ni, B. *J. Org. Chem.* **2000**, *65*, 8532–8543.
7. Métay, E.; Hu, Q.; Negishi, E.-I. *Orga. Lett.* **2006**, *8*, 5773–5776.
8. Roth, G. J.; Liepold, B.; Müller, S. G.; Bestmann, H. J. *Synthesis* **2004**, 59–62.
9. Zhao, X.; Jing, J.; Lu, K.; Zhang, Y.; Wang, J. *Chem. Commun.* **2010**, *46*, 1724–1726.
10. Inamoto, K.; Saito, T.; Hiroya, K.; Doi, T. *Synlett* **2008**, 3157–3162.
11. Gaussian 09, Revision D.01, Frisch, M. J.; Trucks, G. W.; Schlegel, H. B.; Scuseria, G. E.; Robb, M. A.; Cheeseman, J. R.; Scalmani, G.; Barone, V.; Mennucci, B.; Petersson, G. A.; Nakatsuji, H.; Caricato, M.; Li, X.; Hratchian, H. P.; Izmaylov, A. F.; Bloino, J.; Zheng, G.; Sonnenberg, J. L.; Hada, M.; Ehara, M.; Toyota, K.; Fukuda, R.; Hasegawa, J.; Ishida, M.; Nakajima, T.; Honda, Y.; Kitao, O.; Nakai, H.; Vreven, T.; Montgomery, J. A., Jr.; Peralta, J. E.; Ogliaro, F.; Bearpark, M.; Heyd, J. J.; Brothers, E.; Kudin, K. N.; Staroverov, V. N.; Keith, T.; Kobayashi, R.; Normand, J.; Raghavachari, K.; Rendell, A.; Burant, J. C.; Iyengar, S. S.; Tomasi, J.; Cossi, M.; Rega, N.; Millam, J. M.; Klene, M.; Knox, J. E.; Cross, J. B.; Bakken, V.; Adamo, C.; Jaramillo, J.; Gomperts, R.; Stratmann, R. E.; Yazyev, O.; Austin, A. J.; Cammi, R.; Pomelli, C.; Ochterski, J. W.; Martin, R. L.; Morokuma, K.; Zakrzewski, V. G.; Voth, G. A.; Salvador, P.; Dannenberg, J. J.; Dapprich, S.; Daniels, A. D.; Farkas, O.; Foresman, J. B.; Ortiz, J. V.; Cioslowski, J.; Fox, D. J. Gaussian, Inc., Wallingford CT, 2013.

12. (a) Becke, A. D. *J. Chem. Phys.* **1993**, *98*, 5648; (b) Lee, C.; Yang, W.; Parr, R. G. *Phys. Rev. B* **1988**, *37*, 785.
13. Peng, C.; Ayala, P. Y.; Schlegel, H. B.; Frisch, M. J. *J. Comput. Chem.* **1996**, *17*, 49.
14. (a) Gonzalez, C.; Schlegel, H. B. *J. Chem. Phys.* **1989**, *90*, 2154. (b) Gonzalez, C.; Schlegel, H. B. *J. Phys. Chem.* **1990**, *94*, 5523.
15. Jmol: an open-source Java viewer for chemical structures in 3D. http://www.jmol.org/.
